# Thermal impacts on transcriptome of *Pectoralis major* muscle collected from commercial broilers, Thai native chickens and its crossbreeds

**DOI:** 10.5713/ab.23.0195

**Published:** 2023-10-31

**Authors:** Yuwares Malila, Tanaporn Uengwetwanit, Pornnicha Sanpinit, Wipakarn Songyou, Yanee Srimarut, Sajee Kunhareang

**Affiliations:** 1 National Center for Genetic Engineering and Biotechnology (BIOTEC), National Science and Technology Development Agency (NSTDA), Khlong Nueng, Khlong Luang, Pathum Thani 12120, Thailand; 2 Department of Animal Science, Faculty of Agriculture, Khon Kaen University, Khon Kaen, 40002, Thailand

**Keywords:** Chickens, Gene Expression, Native Breed, RNA-Seq, Thermal Stress

## Abstract

**Objective:**

The main objective of this study was to define molecular mechanisms associated with thermal stress responses of chickens from commercial broilers (BR, Ross 308), Thai native chickens (NT) and crossbreeds between BR×NT (H75).

**Methods:**

Twenty days before reaching specific market age, chickens from each breed were divided into control and thermal-stressed groups. The stressed groups were exposed to a cyclic thermal challenge (35°C±1°C for 6 h, followed by 26°C±1°C for 18 h) for 20 days. Control group was raised under a constant temperature of 26°C±1°C. *Pectoralis major* (n = 4) from each group was collected for transcriptome analysis using HiSeq Illumina and analysis of glycogen and lactate. Gene expression patterns between control and thermal-stressed groups were compared within the same breeds.

**Results:**

Differentially expressed transcripts of 65, 59, and 246 transcripts for BR, NT, and H75, respectively, were revealed by RNA-Seq and recognized by Kyoto encyclopedia of genes and genomes database. Pathway analysis underlined altered glucose homeostasis and protein metabolisms in all breeds. The signals centered around phosphatidylinositol 3-kinase (PI3K)/Akt signaling, focal adhesion, and MAPK signaling in all breeds with slight differences in molecular signal transduction patterns among the breeds. An extensive apoptosis was underlined for BR. Roles of AMPK, MAPK signaling and regulation of actin cytoskeleton in adaptive response were suggested for H75 and NT chickens. Lower glycogen content was observed in the breast muscles of BR and NT (p<0.01) compared to their control counterparts. Only BR muscle exhibited increased lactate (p<0.01) upon exposure to the stress.

**Conclusion:**

The results provided a better comprehension regarding the associated biological pathways in response to the cyclic thermal stress in each breed and in chickens with different growth rates.

## INTRODUCTION

Climate change has become one of the most critical challenges for the global food system as it impacts every life on earth including livestock. Upon exposure to heat stress, animals alter their behavior and physiology to release heat and sustain body temperature. Chickens, particularly broilers, are among those affected animals as they can tolerate a narrow temperature range for maximum productivity [1]. Housing at high temperature compromises growth performance, modulates immune response and increases mortality among broilers [2,3]. Meat quality is also influenced by thermal stress. In comparison to the control, breast meat of the experimentally stressed chickens contained higher fat but lower protein content with tough texture and reduced water holding capacity [4]. The negative effects of heat stress appear to be more pronounced in high-performing commercial broilers as the birds with higher age and weight showed more thermal sensitivity [5,6]. A rapid temperature elevation exerts an increased severe impact on the chickens [1]. In this regard, the climate crisis not only affects the availability but also the quality of chicken meat.

Molecular mechanisms associated with heat-stress re sponse in chickens has been under investigation to obtain a better insight for development of heat-stress mitigation approach. Upon an exposure to heat challenge, chickens reduce their feed intake to avoid metabolic heat production [4,5]. On the other hand, the stress triggers accelerated metabolic rate, oxidative stress and inflammation in chicken breast muscle [5]. Heat stress tends to delay synthesis of most proteins except for heat shock proteins [7]. Recently, Ma et al [7] reported that commercial broilers subjected to thermal stress (32°C, 7 days during the age of 28 to 35 days old) exhibited increased muscle protein breakdown through a modified expression of muscle atrophy F-box and the 70-kD ribosomal protein S6 kinase. Their findings indicated that under heat stress, muscle protein undergoes degradation in order to provide amino acid substrates to liver gluconeogenesis. Other studies have also documented an influence of thermal stress on altered metabolisms of chicken muscle proteins. Ito et al [8] reported an increase in free amino acid concentrations in the blood, brain and muscle, implying protein breakdown, in chicks exposed to short-term heat stress (35°C, for 15 to 30 min). Zuo et al [9] found that transcript abundance of insulin-like growth factor 1 (IGF1) and phosphatidylinositol 3-kinase (PI3K) was decreased in the breast muscles of Arbor Acres broilers exposed to a constant heat stress (34°C) during the age of 29 to 49 days, linking with reduced protein synthesis within the muscle. Disorganization of actin cytoskeleton was addressed at the earliest events when cells were exposed to heat stress and later contributed to cell integrity [2,3]. Such alteration might be carried over to impact chicken meat quality. In addition, heat stress disturbs the balance between the production of reactive oxygen species (ROS) and the antioxidant systems, leading to oxidative stress. Oxidative stress can further induce protein backbone fragmentation and modification of amino acid side chains. The latter case could decrease proteolytic susceptibility of the modified proteins. Oxidative damages could cause Ca^2+^ leakage that further accelerates the rate of postmortem pH decline within the muscle and impact meats protein functionality [3]. The stress condition has recently been linked with the damage of muscle cell membrane and cell apoptosis that further led to increased drip loss and poor broiler meat quality [10].

Similar to other animals, native chickens can adapt to the ambient temperature fluctuation to a better extent in comparison to modern high-performing broilers. Hence, a molecular deviation in thermal stress response among fast-growing commercial broilers and slow-growing native chickens could be anticipated. In our recent study [4], we investigated the effects of cyclic thermal stress at a later age on production performance and meat quality among fast-growing commercial broilers (BRs), slow-growing Thai native (NT) chickens and medium-growing ones (H75) obtained from crossbreeding between BR and NT. The results indicated that upon an exposure to the thermal stress at later age, the growth performance of H75 and NT chickens were compromised but to a lesser extent compared with BRs. No significant alteration in meat quality indices was found for NT and H75. However, biological responses of those medium-growing and slow-growing chickens to thermal stress remain to be elucidated.

The objective of this study was to define biological path ways associated with the response to the cyclic thermal stress in the breast muscle of BR, NT, and H75 chickens. In this study, transcriptional profiles of *Pectoralis major* (*P. major*) muscle were compared between control and thermal-stressed samples within each chicken breed using RNA-Seq. The findings of this study would provide a better biological insight for the development of medium-growing chickens with high-quality meat and a better capability of thermal stress adaptation.

## MATERIALS AND METHODS

### Samples and sample collection

The study was approved by the Institutional Animal Care and Use Committee at National Center for Genetic Engineering and Biotechnology (IACUC No. BT-Animal 13/2564).

All samples were collected from the same set of animals used in our previous study [4]. In brief, the animal experiment was carried out at the Department of Animal Science, Faculty of Agriculture, Khon Kaen University (Khon Kaen, Thailand). One-day-old commercial broilers (BR, Ross 308) chicks were obtained from a local hatchery (Charoen Pokphand Company, Nakhon Ratchasima, Thailand). The chicks of NT and H75 (crossbred; 75% BR and 25% NT) were obtained from the Department of Animal Science, Faculty of Agriculture, Khon Kaen University (Khon Kaen, Thailand). All chicks were raised under an environmentally controlled poultry facility, according to the standard practice for commercial meat-type chickens. Feed and water were provided *ad libitum*. All birds were tagged for individual identification.

Twenty days before the chickens reached specific market ages, the birds from each strain were randomly divided into two groups of control and thermal-stressed. The control group was raised under a constant temperature of 26°C±1°C while the heat-stressed group was subjected to a cyclic thermal challenge (35°C±1°C for 6 h, followed by 26°C±1°C for 18 h) as described previously [4]. Each group comprised four replications (four pens) with a stocking density of 5 birds/m^2^. Feed and water were provided *ad libitum*. The thermal challenge was conducted for 20 days.

At the end of the challenge, BR, H75, and NT reached 42, 56, and 84 days of age, respectively. Chickens proceeded to slaughtering processing line with 12 h fasting before the slaughter. *Pectoralis major* muscle was collected within 20 min postmortem from four male chickens per group (one bird per pen replicate), hence a total of 24 samples for RNA-Seq. Muscle samples were excised from the cranial portion, about 1 cm deep from the ventral surface of the muscle, and snap frozen in liquid nitrogen. All samples were kept in liquid nitrogen during the transportation to Food Biotechnology Research Team, National Center for Genetic Engineering and Biotechnology (BIOTEC, Pathum Thani, Thailand). The samples were then immediately stored at −80°C upon arrival for further analyses.

### Total RNA isolation

Total RNA was isolated from the frozen muscle using TriReagent (Molecular Research Center, Inc., Cincinnati, OH, USA) following the manufacturer’s instruction. Contaminated DNA was removed by incubating total RNA with RQ1 RNase-free DNase (Promega Corporation, Madison, WI, USA) at 37°C for 30 min. Subsequently, total RNA was purified using a column-based GeneJET RNA Cleanup and Concentration Micro Kit (Thermo Scientific, Inc., Rockford, IL, USA). Concentration of total RNA samples was then quantified using a Nanodrop (Thermo Scientific, Inc., USA). Integrity of total RNA samples was preliminarily determined using a 5200 Fragment Analyzer system (Agilent, Santa Clara, CA, USA). Only the samples with RNA quality number greater than 7.0 (RQN >7.0) were proceeded to transcriptome analysis.

### Transcriptome analysis

Transcriptome analysis was carried out at a laboratory of Vishuo Biomedical (Thailand) Ltd (Bangkok, Thailand). Prior transportation, 2.5 μg of total RNA samples was mixed with nuclease-free water to a final volume of 20 μL, transferred into a GenTegra-RNA tube (Gentegra LLC., Pleasanton, CA, USA), and dried using a SpeedVac for 2 h. Upon arrival, the samples were resuspended using nuclease-free water. RNA integrity and concentration were determined using an Agilent 2100 Bioanalyzer (Agilent Technologies, Palo Alto, CA, USA) and Nanodrop, respectively, to ensure RNA quality and quantity. The samples with RNA integrity number (RIN) >7.0 were then proceeded for RNA library construction following the company’s instruction (NEBNext Ultra RNA Library Prep Kit; New England Biolabs, Inc., Ipswich, MA, USA). All libraries were bar-coded and sequenced using an Illumina HiSeq (Illumina, San Diego, CA, USA) to obtain paired-end 2×150 bp reads. The resulting sequencing reads were analyzed using HiSeq Control Software (HCS)+OLB+GAPipeline-1.6 (Illumina, USA).

### Transcriptome data analysis

**Figure 1:**
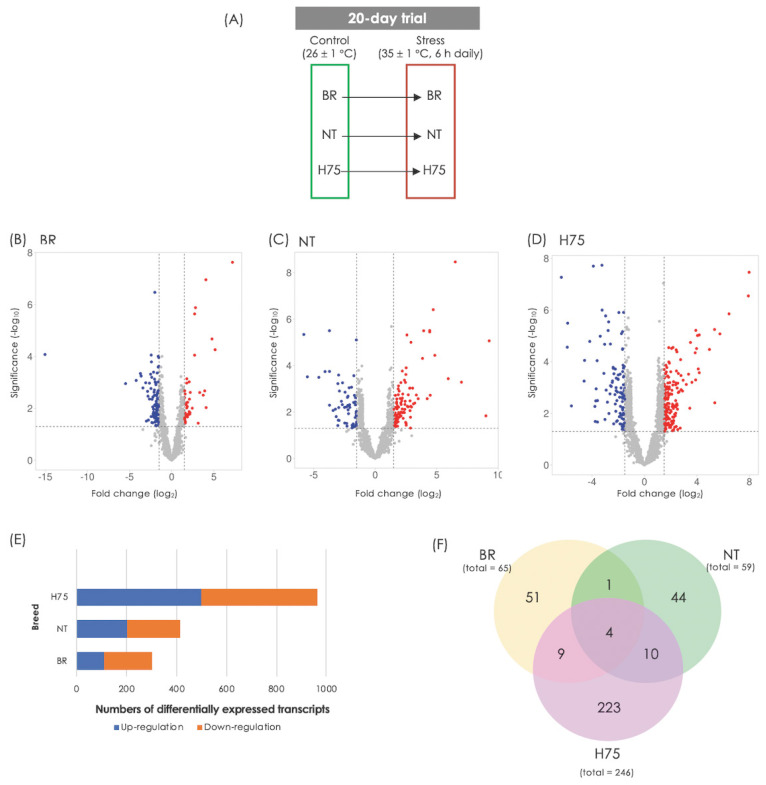
Differences in transcript abundance associated with cyclic thermal stress. (A) Experimental design. Transcriptome profiles of breast muscle collected from stressed broilers (BR), native Thai Chicken (NT) and crossbreeds between BR×NT (H75) were compared with their control counterparts. (B, C, D) Volcano plots of BR, NT and H75, respectively. (E) Numbers of differentially expressed transcripts (DETs) identified from each comparison. The criteria used in identification of DETs is a combination between p≤0.05 and absolute log_2_fold change (|log_2_FC|) ≥1.0. (F) Venn diagram illustrates number of DETs which are recognized by Kyoto encyclopedia of genes and genomes (KEGG) database and mapped into KEGG pathways.

Illumina paired-end reads were processed to remove the adapter and low-quality bases at both ends (Phred quality score <20) using TrimGalore. Trinity genome-guided assembly was employed to build a comprehensive transcriptome. Genome Reference Consortium Chicken Build 6a (*Gallus gallus*) was used as the reference genome. To reduce the number of potential spurious contigs, expressed transcripts with count per million >1 were retained for further analysis. The assembled transcripts were compared to the conserved vertebrate genes using Benchmarking Universal Single-Copy Ortholog (BUSCO, vertebrata odb10) to evaluate their completeness. The preprocessed reads were aligned using Bowtie2. Expression levels were determined using HTSeq and the differential transcript expression were analyzed using DESeq2. Differentially expressed transcripts (DETs) between control and thermal-stressed samples within the same breed (Figure 1A) were assigned when p<0.05 and |log_2_ fold change| values ≥1. Functional and enrichment analyses were analyzed using OmicsBox. Sequences alignments (BLASTX) to the NCBI non-redundant vertebrate proteins, gene ontology (GO) mapping and Kyoto encyclopedia of genes and genomes (KEGG) pathway analysis were employed. The raw RNA-Seq data can be found in BioProject PRJNA815887.

RNA-Seq data was confirmed using a quantitative real-time polymerase chain reaction (qPCR) as shown by Malila et al [11]. A total of 8 genes, resulting for 24 comparison counterparts, was selected for the confirmation. Overall, the qPCR results were consistent with the RNA-Seq data (Supplementary Figure S1).

### Glycogen and lactate assays

Glycogen and lactate in the breast muscles were determined. For glycogen, frozen muscles (100 mg) were heated in 500 μL of 40% KOH at 95°C for 60 min with a constant shaking. After the samples were cooled down to room temperature, 2 mL of 95% ethanol were added. The mixture was centrifuged at 3,000 rpm using an Eppendorf 5810R centrifuge (Eppendorf, Hamburg, Germany) for 10 min. The pellet was collected and resuspended in 900 μL deionized water and acidified to pH 3 using HCl. The extracted glycogen was re-precipitated in 95% ethanol. The final pellet was then air-dried and resuspended in 300 μL of deionized water. The glycogen concentration was subsequently analyzed using a glycogen assay kit (Sigma-Aldrich, St. Louis, MO, USA) following the company’s instruction.

As for lactate, frozen muscles (1 g) were homogenized with 4 mL 1 M perchloric acids for 2 min. Subsequently, pH of the homogenate was adjusted to pH 8.0 using KOH. Distilled water was added to the homogenate to a final volume of 25 mL. The homogenate was then incubated on ice for 20 min, followed by a centrifugation at 13,000×g for 10 min. Supernatant was collected for analysis of lactate concentration using L-lactic acid assay kit (Megazyme, Wicklow, Ireland).

The measurement was performed in triplicate. Lactate and glycogen in the sample were expressed in milligrams per gram muscle sample. Differences in glycogen and lactate between control and thermal-stressed samples within each chicken strain were analyzed using Student’s t-test. Significant level was set at p<0.05.

## RESULTS

### Transcriptome profiling and pathway analysis

Despite the availability of chicken reference genomes (*Gallus gallus*), transcripts in the present study were generated using genome-guided assembly rather than genome reference mapping to reduce the risk of losing unique or species-specific sequences. The total number of reads after cleaning generated for this study was 38,806,816 per sample in average. The assembled transcripts were 53,837 contigs with 2,088 nt in average length (201 to 31,145 nt). BUSCO alignments showed that 83.42% of the BUSCO groups have complete gene representation (single-copy or duplicated), while 3.13% are only partially recovered, and 13.45% are missing. Based on Trinity protocol [30], 80% of read mapping could be considered indicative of a good quality assembly.

Differences in transcript abundance between stressed sam ples and their control counterparts can be observed from volcano plots (Figure 1B–1D). RNA-Seq revealed DETs of 323, 439 and 1,107 for BR, NT and H75, respectively (Figure 1E; Supplementary Table S1–S3). Of those DETs, 65, 59 and 246 transcripts for BR, NT and H75, respectively, were recognized by KEGG database and mapped into KEGG pathways (Figure 1F). Four DETs overlapped among all breeds (Table 1) include L-lactate dehydrogenase A chain isoform X1 (*LDHA*), creatine kinase B-type isoform X1 (*CKB*), collagen alpha-1(XII) chain isoform X1 (*COL12A1*) and troponin T, fast skeletal muscle isoforms (*TNNT3*).

**Figure 2:**
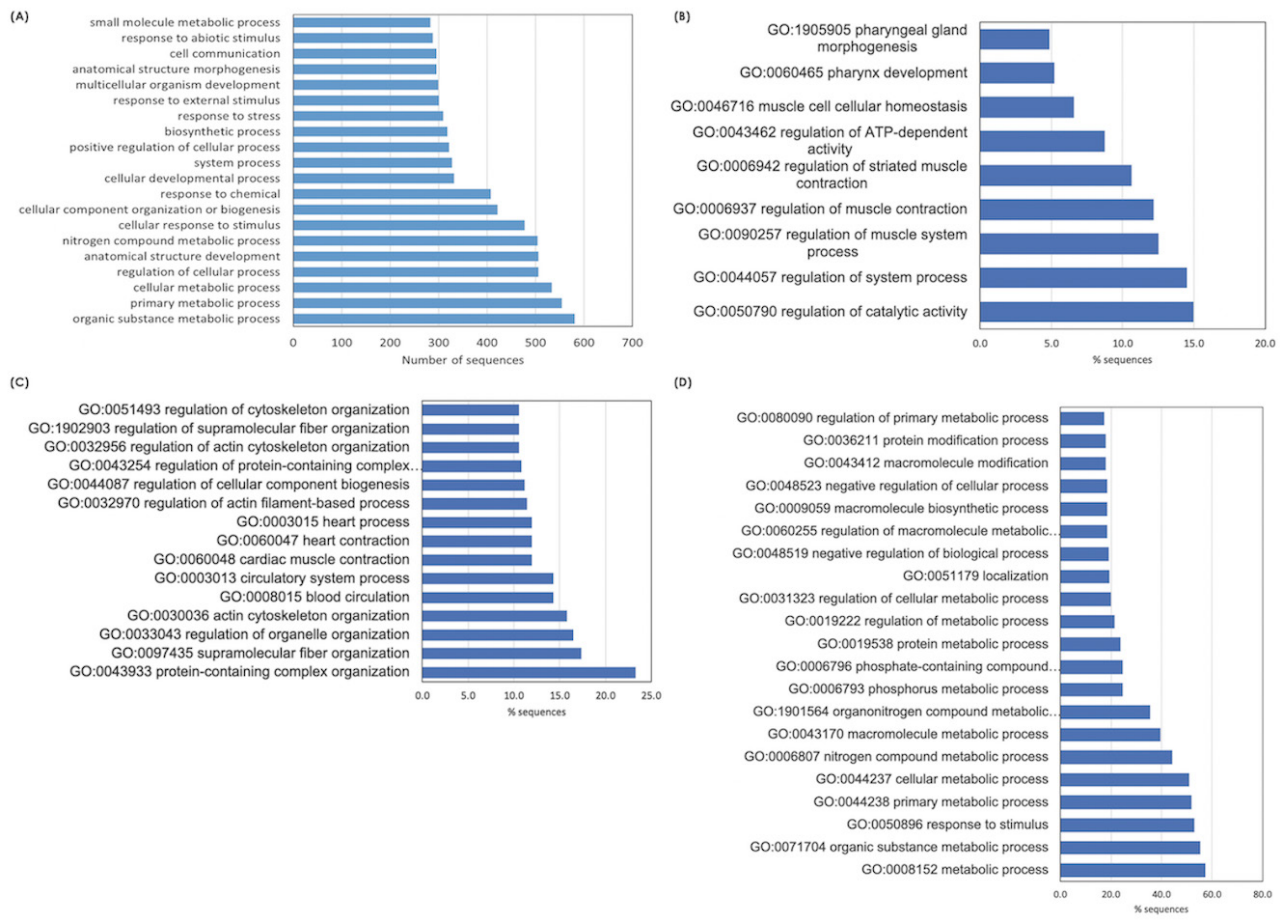
Histogram of the significant differentially expressed transcripts enriched top 20 gene ontology (GO) terms belonging to the category of "Biological Process”. The enriched altered biological pathways identified (A) among all breeds, and within each chicken breeds, i.e., (B) commercial broilers (BR), (C) native Thai Chicken (NT) and (D) crossbreed BR×NT (H75). Enrichment analysis of GO terms calculated by Fisher’s exact test with a p-value threshold of 0.05.

Based on top 20 GO terms of biological process (Figure 2A), The majority of the DETs were associated with organic substance metabolism, primary metabolic processes (i.e., macronutrients and nucleotides) and cellular metabolisms, regulation of cellular process, anatomical structural development, and cellular response to stimuli. Considering each breed, the enriched GO terms of biological process for BR (Figure 2B) involved in regulation of catalytic activity, system process, muscle system process, ATP-dependent activity. In terms of NT (Figure 2C), the DETs identified for this breed appeared to involve structural organization, including protein-containing complex, supramolecular fiber, organelles, actin cytoskeleton, blood and circulatory system. The alteration of metabolic processes of several biomolecules in the stressed H75 is also emphasized by GO results (Figure 2D). On the other hand, the top enriched KEGG pathways identified in BR are metabolic pathways, biosynthesis of amino acids, glycolysis/gluconeogenesis, carbon metabolism, and hypoxia inducible factor-1 (HIF-1) signaling. As for NT, the top altered KEGG pathways include metabolic pathways, regulation of actin cytoskeleton, focal adhesion, glycolysis/gluconeogenesis, biosynthesis of amino acid, and PI3K/Akt signaling. For H75, metabolic pathway, mitogen-activated protein kinase (MAPK) signaling, glycolysis/gluconeogenesis, regulation of actin cytoskeleton, and autophagy, are highlighted as the enriched pathways. Overall, the altered biological processes identified by GO and KEGG within each breed were intersected.

**Figure 3:**
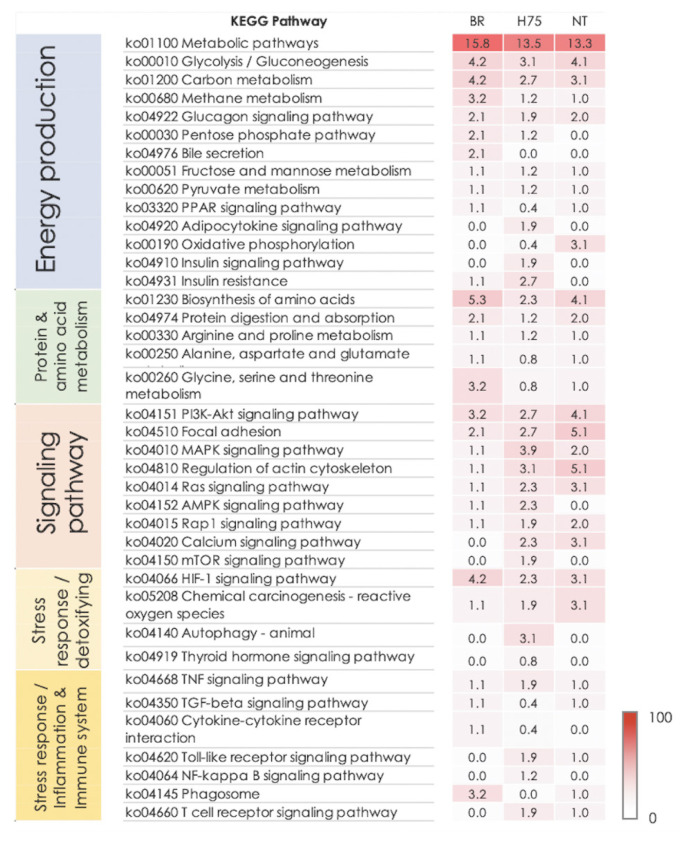
Heatmap shows the enriched altered biological pathways, based on Kyoto encyclopedia of genes and genomes (KEGG), identified in each chicken breeds, i.e., commercial broilers (BRs), native Thai Chicken (NT) and crossbreed BR×NT (H75). The darker color indicated the greater numbers of differentially expressed transcripts mapped into the pathway. The number in heatmap indicates the number of differentially expressed transcripts (DETs) mapped into the pathway by KEGG relative to total DETs.

Considering further, biological pathways associated with glucose homeostasis, metabolism of protein and amino acids, and several cellular signaling modules (i.e., PI3K/Akt signaling, focal adhesion, MAPK signaling, regulation of actin cytoskeleton) were identified in all breeds (Figure 3). On the other hand, some pathways were only found in some breeds. Examples of those are autophagy (identified in H75), adenosine monophosphate-activated protein kinase (AMPK) signaling (identified in BR and H75) and some stress- and immune-response pathways, (e.g., nuclear factor-kappa B [NF-κB] signaling, toll-like receptor signaling, leukocyte transendothelial migration and Ca^2+^-signaling pathways) were commonly identified for H75 and NT (Figure 3).

### Glycogen and lactate content

**Figure 4:**
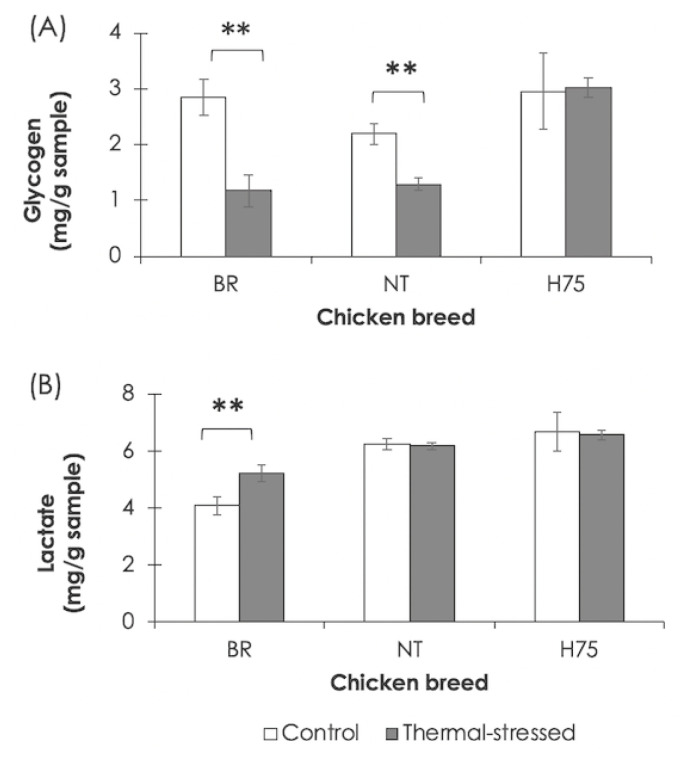
Glycogen and lactate in chicken breast muscles. Bars and error bars show mean and standard errors of (A) glycogen and (B) lactate content detected in breast muscles of each chicken breeds, i.e., commercial broilers (BRs), native Thai Chicken (NT) and crossbreed BR×NT (H75). ** p<0.01.

The effects of the cyclic thermal challenge on glycogen and lactate in the breast muscles are shown in Figure 4. Breast muscles of stressed BR and NT contained lower glycogen content (p<0.01) than that of their control counterparts (Figure 4A). An increase in lactate (p<0.01) was detected for BR breast muscle exposed to the thermal stress (Figure 4B). No significant differences in glycogen and lactate were H75.

## DISCUSSION

One of the proposed solutions for food security is to adopt a local food production that is more sustainable and affordable to meet nutritional requirement. Therefore, a small-scale production of native chickens, e.g., NT chickens, has been suggested as one of the approaches for increasing local food availability and improving food security [12]. However, due to their slow growth rate, the production of native chickens requires a longer rearing period of time and additional resources (e.g., feed, water, and land) for the birds to reach their market weight. Still, the meat yield is lower than that of the fast-growing high-performing commercial broilers. Hence, medium-growing chickens, yielded from a crossbreeding between high-performing and slow-growing strains may overcome such issues of their parents [13].

To define biological pathways associated with thermal stress response in BR, NT, and H75 chickens, transcriptome of *P. major* muscle (breast muscle) of thermal-stressed group was compared with their control counterparts. The findings of each breed will be discussed in detail below.

### Fast-growing commercial broilers

The results highlighted the KEGG pathways associated with glucose homeostasis and energy production, including glycolysis/gluconeogenesis, glucagon signaling and HIF-1 signaling, as the enriched altered pathways in the BR breast muscles (Supplementary Table S4). The findings were agreed with metabolic shifts in the birds exposed to thermal stress [10]. This was not beyond our expectation. Indeed, it is extensively reported that upon an exposure to heat stress, chickens reduce their feed intake to avoid metabolic heat production [13,14] and to reserve resources for cellular adaptation to maintain homeostasis [14]. Glucose is a primary source of energy supply. Increased levels of plasma glucose [3] and glucocorticoids [15] were reported among commercial broilers exposed to heat challenges. Awad et al [3] suggested that the elevated plasma glucose was a consequence of increased plasma glucocorticoids in order to reserve glucose for the brain during stress situations. In fact, upon exposure to heat stress, muscle glycogen utilization increased with no change in glucose uptake by the muscle and decreased carbohydrate oxidation [3,14,15]. This might lead to reduced glycogen and increased lactate in the breast muscle of the stressed BR (Figure 4). The breakdown of glycogen and accumulated lactate could lead to an acidic condition within the poultry breast muscle [16]. Such conditions ultimately impacted meat quality of the stressed BR [10]. In addition, we observed that *LDHA* (log_2_FC = −1.3 to −3.4), fructose-bisphosphate aldolase A (*ALDO*, log_2_FC = 1.2) and alpha-enolase isoform X3 (*ENO1*, log_2_FC = −2.5) were mapped into glycolysis/gluconeogenesis and HIF-1 signaling (Supplementary Table S4). The findings suggested the closed interplay of those pathways in response to the cyclic thermal stress within the breast muscle of BR.

The KEGG biosynthesis of amino acids was among the enriched altered pathways impacted by the cyclic thermal stress in the BR breast muscles (Supplementary Table S4). Differential expressions of genes encoding glycolytic enzymes, including *ALDO*, *ENO1*, and bisphosphoglycerate mutase isoform X1 (*PGAM*, log_2_FC = −1.1), were also mapped into biosynthesis of amino acids. This could be due to the interrelationships between glucose and protein as the precursor pools for energy production. Additionally, the carbon chains for some amino acids are the intermediates of glycolysis. The current results are consistent with previous studies showing an increased muscle protein degradation upon exposure to thermal stress [7–9]. The catabolism of protein and amino acids has been hypothesized to reflect an energy demand for the animals to counter heat stress [7,9].

Changes in gene expression patterns in the stressed BR also suggested the oxidative stress condition within the breast muscle. Cystathionine-β-synthase (*CBS*, log_2_FC = 2.2), glutathione S-transferase (*GSTA4*, log_2_FC = 1.4) and ribose-5-phosphate isomerase (*RPIA*, log_2_FC = 1.3) were up-regulated in the thermal-stressed BR muscles (Supplementary Table S1; Table S4). The CBS enzyme catalyzes metabolisms of sulfur-containing amino acids, i.e., methionine and cysteine, through an irreversible conversion of homocysteine to cystathionine, the upstream process of glutathione formation [17]. Increased *CBS* and *GSTA4* abundance in the stressed BR might imply an autocorrective function of CBS to oxidative condition through glutathione system [17]. As for RPIA, the enzyme catalyzes the process of histidine biosynthesis and formation of nucleotides or glycolytic intermediates depending on body condition [18]. The crucial role of RPIA in mediating ROS level, apoptosis and autophagy was addressed in lung cancer cells [18]. Furthermore, differential expression of gene encoding glutamine-fructose-6-phosphate aminotransferase (isomerizing) 2 isoform X2 (*GFPT2*, log_2_FC = −1.1) suggested the altered hexosamine pathways in the thermal-stressed BR. The pathway is activated in response to an intracellular oxidative stress linked to hyperglycemia and insulin resistance [19]. The end product of hexosamine pathway, uridine diphosphate N-acetyl glucosamine, can further be converted to O-linked N-acetyl glucosamine, a crucial intracellular signal transductor that enhances the cyto-protective effects in stressed cells [19].

PI3K/Akt signaling pathway, phagosome and focal adhesion are also among the top enriched altered pathways for BR samples. Differentially expressed integrin alpha 11 (*ITGA11*, log_2_FC = −1.3), cartilage oligomeric matrix protein (*COMP*, log_2_FC = −2.0) and death domain-containing membrane protein isoform X3 (*NRADD*, log_2_FC = −1.2) were mapped into those pathways. The proteins encoded by those genes facilitate cell communication in different manners. *COMP* encodes a secretory non-collagenous extracellular matrix pentamer glycoprotein, also known as thrombospondin-5, that activates PI3K/Akt signaling pathways when it binds with an integral membrane receptor. Such signal has shown to suppress apoptosis and promote proliferation and migration in cancer cells. Integrins and NRADD are transmembrane receptors activated by the binding of their cognate ligands to the extracellular signals [20]. Down-regulation of *ITGA11* was found in chickens exposed to oxidative stress via heat stress and toxic minerals [4]. NRADD, belonged to nerve growth factor receptor (NGFR) superfamily, allows the cells to receive apoptotic signals, hence activating cell death when the cells are exposed stress [20]. Increased abundance of major histocompatibility complex (MHC) class II beta chain (*BLB1*, log_2_FC = 3.1) in the phagosome pathway may imply the clearance process of cellular debris upon the oxidative damages and apoptosis. The role of nicotinamide adenine dinucleotide (NAD) phosphate (NADPH) oxidase 2 (NOX2), encoded by cytochrome b-245 beta chain (*CYBB*, log_2_FC = −1.2), in regulating apoptotic cell removal alkalization of phagosome has been reported [21].

The current RNA-Seq findings for BR breast muscles un derlined metabolic adaptation as the key altered pathways in response to the cyclic thermal stress. The occurrence of cellular damage, potentially from oxidative stress, and cellular debris clearance was emphasized in the BR breast muscles. Similar molecular alteration was recently underlined and associated with the impact of chronic heat stress on reduced breast meat quality in Arbor Acres broilers [10].

### Slow-growing Thai native chickens

Previous report indicated that indigenous chickens can tolerate and adapt to high ambient temperature to a greater extent than fast-growing commercial broilers [5]. No adverse effects of heat stress on production performance and meat quality are extensively reported. Nevertheless, previous studies showed that thermal challenges stressed the slow-growing native birds as well [4,13]. Deviated plasma triglyceride, plasma glucose and serum total protein were also reported in the 21-day-old slow-growing chicks exposed to heat stress (38°C, 4 h daily) for 3 days [22]. Thermoregulatory capabilities of slow-growing chickens indeed vary among the different strains [23]. In this study, decreased glycogen content in the breast muscle of stressed NT but no change in lactate content was detected (Figure 4).

Based on the current RNA-Seq (Supplementary Table S5), glycolysis/gluconeogenesis, glucagon signaling, metabolisms of proteins and amino acids, and HIF-1 signaling were the top enriched pathways identified for the NT samples. In addition to those pathways, metabolisms of fructose, mannose, pyruvate, and nucleotides were mediated within the NT breast muscles. Besides, as observed in the BR, the signaling pathways (Figure 3), including PI3K/Akt, focal adhesion, MAPK, and Ras signaling, were altered in NT breast muscles. Even though the list of DETs (Supplementary Table S5) mapped into those pathways was slightly different from that of BR samples, the results suggested some similarities in metabolic shifts and the cellular signals between BR and NT birds when they were facing the cyclic thermal challenge.

Interestingly, oxidative phosphorylation is among the en riched altered pathways for NT chickens but not for the BR group (Figure 3). The DETs mapped into this pathway include ATP synthase F0 subunit 6 (*ATPeF0A*, log_2_FC = 5.7), cytochrome c oxidase subunit 2 (*COX2*, log_2_FC = 2.7) and COX subunit 3 (*COX3*, log_2_FC = 1.7). In addition, NADH dehydrogenase subunit 1 (log_2_FC = 1.6), subunit 2 (log_2_FC = 2.8), subunit 3 (log_2_FC ranging from 1.4 to 3.2) and subunit 4 (log_2_FC ranging from 1.4 to 3.5) along with NADH-ubiquinone oxidoreductase chain 5 isoform X2 (log_2_FC = −1.4) were differentially expressed between thermal-stressed and control NT birds. Those genes encode subunits of the Complex I of the mitochondrial electron transport chain (ETC). Oxidative phosphorylation is the final step in cellular respiration occurred in mitochondria. Up-regulation of those genes suggested that the Thai NT chickens modified mitochondrial ATP synthesis in response to the cyclic thermal challenge.

Furthermore, vascular endothelial growth factor (VEGF) D isoform X1 (*VEGFD*, log_2_FC = 1.2) were up-regulated within the thermal-stressed NT. VEGF is a potent angiogenic factor, regulating a wide range of responses including metabolic homeostasis, cell proliferation and migration, as well as formation and maintenance of blood vessel structure. Transcript abundance of fibroblast growth factor (FGF) 16 isoform X1 was also increased in the stressed NT (log_2_FC = 1.2). Together, VEGF and FGF signaling might promote angiogenesis, improving oxygenation in the stressed NT breast muscle [24]. In addition, increased *VEGF* and integrin subunit beta 3 (*ITBG3*) with decreased myosin light chain isoform 9 (*MYL9*) were associated with immune suppression of Jersey cattle under thermal stress [24]. In this study, we found differential expression of myosin light chain 1 skeletal muscle isoform X1 (*MYL1*, log_2_FC ranging between −1.4 to 9.0, recognized as MYL9 by KEGG) and integrin alpha-D isoform X13 (*ITGAD*, log_2_FC = 2.5) in the breast muscle of NT chickens. Additionally, serine/threonine-protein kinase isoform X1, encoding the p21 activated kinase (PAK) 6 was up-regulated (*PAK6*, log_2_FC = 1.4). PAK6, a member of the downstream effectors of Ras-related Rho GTPase Cdc42 and Rac, facilitates cytoskeletal organization, cell motility and apoptosis in response to stress. Such transcriptional modifications of those genes in association with toll-like receptor and T cell receptor signaling pathways might attenuate the adverse effects [25], particularly from oxidative stress, in the NT muscle. Therefore, the properties of NT breast meat did not significantly differ between the control and the thermal-stressed groups [4].

### Medium-growing crossbred H75

The numbers of DETs and the altered pathways identified for H75 were higher than that of BR and NT. Nonetheless, the top altered metabolic pathways and signaling pathways identified for H75 were similar to those found for BR and NT (Supplementary Table S6). In addition to metabolic shifts, mitochondrial *ETC* genes, including, NADH-ubiquinone oxidoreductase (log_2_FC ranging between 1.1 and 1.6), cytochrome b (*CYB*, log_2_FC = 1.1), *COX2* (log_2_FC ranging between 1.1 and 1.3), *COX4* (log_2_FC = 1.5), were differentially expressed between stressed H75 and their control counterparts (Supplementary Table S3; Table S6). Changes in the abundance of those genes might imply adaptive mitochondrial ATP synthesis in the stressed H75 breast muscle in a similar manner as observed in the NT breasts. No differences in either muscle glycogen or lactate were observed in the breast muscles between control and stressed NT (Figure 4).

Interestingly, adipocytokine signaling, insulin signaling, autophagy, mammalian target of rapamycin (mTOR) and NF-κB signaling pathways were specifically listed for the H75 (Figure 3). Alteration of those pathways was identified in broilers exposed to chronic heat stress [7]. Those pathways were shown to involve in fat deposition in broilers [26]. Zhang et al [26] addressed that phosphoenolpyruvate carboxykinase 2 (*PCK2*), acetyl-CoA carboxylase 1 alpha and beta (*ACACA* and *ACACB*), and *AMPK* gene family are the key genes regulating fat deposition in muscle and fat tissues of broilers. In this study, we observed an increased *ACACB* (log_2_FC = 1.6) and decreased 5′-AMP-activated protein kinase catalytic alpha subunit (*PRKAA2*, log_2_FC = −1.1), a catalytic subunit of AMPK, in the stressed H75. However, in our previous study, no significant differences in abdominal fat and fat content were observed in the carcass and breast meat, respectively, between control and H75 chickens [4]. The score for adipose infiltration of stressed H75 breast muscle was not statistically different from that of their control counterparts. The reason for the discrepancy needs further investigation. The results, however, suggested the role of lipid metabolism in metabolic modification in H75 exposed to the cyclic thermal stress. In addition to that, adipocytokines released from adipose tissues are often altered in obese subjects, of which the levels of proinflammatory cytokines and ROS are increased. Signals from adipocytokines might assist inflammatory responses and maintenance of systemic energy metabolism under the oxidative stress condition within the muscle of the stressed H75.

A dynamic interplay between autophagy and mTOR sig naling pathways also regulates a recycle of damaged organelles and macromolecules for energy and building blocks for normal growth. Such action is dictated by nutrient and energy status with the coordination of AMPK signaling. Recently, Tang et al [27] showed that heat stress (35°C±2°C, 8 h daily, 7 and 14 days) decreased autophagic activity in the liver of broilers, corresponding with liver inflammation. In addition, similarly observed in NT, toll-like receptor and T cell receptor signaling pathways were altered in H75 (Figure 2B). In this study, those altered pathways along with NF-κB signaling were linked together by differential expression of phosphatidylinositol 3-kinase regulatory subunit alpha isoform X1 (*PIK3R1*, log_2_FC = −1.4), NF-κB inhibitor alpha (*NFKBIA*, log_2_ FC = 1.5), dual specificity mitogen-activated protein kinase kinase 4 (*MAP2K4*, log_2_FC = −1.2), *MAP2K6* (log_2_FC = −1.1), *PAK2* (log_2_FC = −1.1) and novel protein kinase C theta type (*PRKCQ*, log_2_FC = −1.2) (Supplementary Table S3). PAK2 involves in heat shock-induced apoptotic cell death process in mammalian cells. Incorporated signals of those pathways are essential for precise regulation of the immune system against infections and inflammatory diseases. Hence, those unique altered pathways might be the key system assisting the adaptation and maintenance of the H75 chickens upon exposure to the cyclic thermal challenge, leading to less detrimental effects on breast meat quality compared with BR [4].

### Comparing differential transcriptome profiles among breeds

Although different lists of DETs among the three breeds were observed, the overall data ascertained modification of metabolic homeostasis, potentially under cellular oxidative stress condition, among all breeds [7]. Carbohydrate metabolisms were impacted in all breeds with some differential mechanisms. Glycolysis/gluconeogenesis was enriched in all breeds. It has been reported that, in poultry, thermal stress led to increased mobilization of glucose to supply energy [10]. The muscle glycogen would undergo breakdown at a greater rate in the stressed chickens leading to lower muscle glycogen and higher lactate [10,15,16]. Our results underlying decreased muscle glycogen and increased lactate in the breast muscle of the stressed BR (Figure 4) agreed with previous studies [10,15]. Among NT, reduced muscle glycogen was also detected when the birds were under the cyclic thermal stress (Figure 4A). However, no differences in lactate was observed between the NT control and stressed groups. The results agreed with the current transcriptome analysis suggesting an adaptive mechanism, particularly the promoted angiogenesis, within the NT that attenuated muscle lactate accumulation. On the contrary, no differences in muscle glycogen and lactate were found between control and stressed H75. The explanation required further investigation.

**Figure 5:**
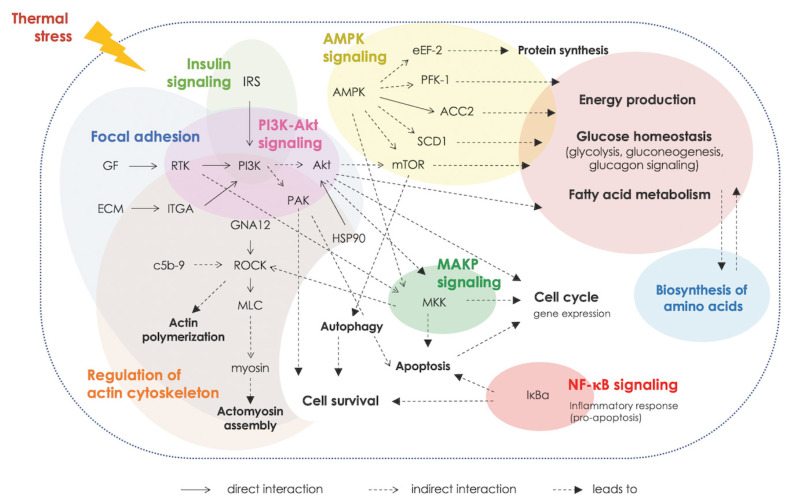
Interaction, based on Kyoto encyclopedia of genes and genomes (KEGG), among enriched biological pathways associated with cyclic thermal stress.

Altered metabolisms of protein and amino acids were highlighted. The analysis of enriched KEGG pathways revealed the association of the DETs in regulation of actin cytoskeleton along with enriched GO term related regulation of muscles. The results suggested the increased muscle protein degradation upon exposure to thermal stress [7–9]. The cyclic thermal stress also affected signal transduction, inflammatory response, immune system, programmed-cell death as well as the process of cellular function (e.g., cell growth, cell differentiation and cell migration). The upstream signals for those biological response appeared to be centered around the PI3K/Akt signaling in coordination with mTOR, AMPK, and MAPK signaling (Figure 5). The PI3K/Akt, MAPK and AMPK pathways are essential for metabolic control and also involved in fundamental cellular processes, e.g. cell growth and cell survival. Crosstalk among those pathways when cells were under stress has been demonstrated [10]. The findings were in line with previous reports [7,10] and suggested the important roles of those pathways in chicken breast muscle in response to the cyclic thermal challenges.

Interestingly, alteration of AMPK signaling was found for BR and H75 but not for NT samples. Only stearoyl-CoA desaturase (*SCD1*, log_2_FC = −2.2) was listed in the AMPK signaling pathway for BR. Decreased *SCD1* expression was found during the onset of cell death due to cytotoxic stress. SCD1-derived lipokine was shown to attenuate cell damage due to cytotoxic stress by limiting cell apoptosis through inhibition of MAPK signaling and rerouting cell fate to autophagy. Since heat stress was shown to induce oxidative stress which further led to MAPK-mediated cell apoptosis, the results suggested extensive apoptosis in the BR muscle which might negatively affect breast meat quality [4,10].

In contrast to BR, more DETs were involved in the AMPK signaling pathways for H75. A similar trend was also found for the regulation of the actin cytoskeleton. AMPK involves in mitochondrial homeostasis by removing the defective mitochondria and stimulating de novo mitochondria synthesis, hence controlling mitochondrial adaptability under stress. Skeletal muscle of meat-type chickens exhibited mitochondrial oxidative phosphorylation of skeletal muscle exhibited a higher efficiency in comparison to that of laying type chickens. However, the rapid metabolic rates in the early life of the fast-growing broilers might exert constraints on oxidative capacity among modern broilers. Therefore, the ability of BR chickens to adapt to the oxidative stress under the cyclic thermal challenge might be to a much lesser extent.

An exposure of skeletal muscle to heat stress has been ex tensively associated with increased expression of heat shock protein (HSP), particularly *HSP70* and *HSP90* [28]. Those proteins function to protect the cells against the stress through inducing a series of cellular defense mechanisms, including antioxidant system, NF-κB and PI3K/Akt signaling. However, in this study, no significant differences in *HSP* abundance were observed in either BR or NT. Rimoldi et al [29] also observed the lack of increased *HSP70* and *HSP90* in hepatic tissues of broilers exposed to 4-week heat stress. They hypothesized that either the expression levels of those genes had already declined upon a long-term heat exposure or *HSP* mRNA was lost due to cell lesion. The speculation of Rimoldi et al [29] appears to agree well with our observation for BR and NT of which the changes in the HSP downstream pathways, particularly PI3K/Akt and antioxidant enzymes, were observed. However, further experiments are required to support this hypothesis. As for H75, changes in transcript abundance of heat shock cognate protein HSP 90-beta isoform X1 (*HSP90AB1*, log_2_FC = −1.1), heat shock factor protein 2 (*HSF2*, log_2_FC = 1.1) and α-crystalline B chain isoform X1 (*CRYAB*, log_2_FC = 4.2), which is a small HSP, were observed between thermal-stress and their control counterparts. Inconsistent with our transcriptome results, overexpression of *CRYAB* was shown to increase survival of myocardial cells subjected to heat stress through the processes of actin cytoskeleton stabilization and prevention of apoptosis.

Comparing blood transcriptomes between chickens ex posed to heat stress and control, Kim et al [14] also found focal adhesion among the enriched biological pathways. They speculated that focal adhesion, together with Ca^2+^ signaling, contributed to an upregulation of heat shock protein genes which further activate MAPK signaling pathways [14]. However, although focal adhesion was underlined in all breeds, no alteration in Ca^2+^ signaling was observed in the BR samples. Taken together, it may be reasonable to speculate that those pathways may contribute to adaptive mechanisms in the H75 breast muscle under the cyclic thermal stress. The BR chickens were able to activate stress-response pathways to a lesser extent in comparison to those of H75 and NT. Further up-close investigation remains to be elucidated.

In this study, the effects of the cyclic thermal stress were focused on the later age (three weeks before market age) of BR, NT, and H75. This is due to the fact that the birds with higher age and weight showed more thermal sensitivity [5,6]. Hence, the loss of chickens at this stage would bring about a significant economic loss. The differences in age of those chickens are, however, worth noting. In addition, transcriptome analysis captures a snapshot of the total transcripts presenting in the cells under a certain circumstance [30]. Either the DETs showed up- or down-regulation in this study might not precisely reflect their immediate responses to the stress. Further investigation remains to be elucidated for such aspects. However, it is certain that the molecular pathways of the DETs involved would be altered by the stress. In addition, inflammatory response is majorly reported in broilers upon heat exposure [30]. Nonetheless, in this study, only NT and H75 showed pathways associated with inflammation. The discrepancies might be due to the different stress intensity between this study and the previous ones.

In conclusion, the current RNA-Seq analysis provides in sights into biological responses in breast muscles of BR, NT, and H75 chickens exposed to the cyclic thermal stress. The stress triggered metabolic shifts in all breeds with the signals centered around PI3K/Akt signaling, focal adhesion, and MAPK signaling. Different molecular signal transduction patterns were observed. The findings underlined the key roles of AMPK, MAPK signaling and regulation of actin cytoskeleton in H75 and NT chickens as an adaptive system against cyclic thermal stress.

## Figures and Tables

**Table 1:** Differentially expressed transcripts found in all breeds

NCBI Accession number	Gene ID	KEGG ID	Sequence description	BR	NT	H75
NP_990615.2	*LDHA*	K00016	L-lactate dehydrogenase A chain	−1.8	−1.2	1.1
NP_990838.1	*CKB*	K00933	creatine kinase B-type isoform X1	2.2	2.2	−2.1
XP_015140228.1	*COL12A1*	K08132	collagen alpha-1(XII) chain isoform X1	−2.3	−2.1	2.8
XP_046797375.1	*TNNT3*	K12046	troponin T, fast skeletal muscle isoforms	2.7	−1.2	−3.9

## SUPPLEMENTARY MATERIAL

**Supplementary Figure S1**. Primers (a) and scatter plots (b) from qPCR analysis for confirmation of RNA-Seq results.

**Supplementary Table S1**. Differentially expressed transcripts (DETs) associated with thermal stress in P. major muscle of commercial broilers. Only annotated transcripts (from 323 DETs) are presented.

**Supplementary Table S2**. Differentially expressed transcripts (DETs) associated with thermal stress in P. major muscle of Thai Native chicken. Only annotated transcripts (from 439 DETs) are presented.

**Supplementary Table S3**. Differentially expressed transcripts (DETs) associated with thermal stress in P. major muscle of crossbred H75. Only annotated transcripts (from 1,107 DETs) are presented.

**Supplementary Table S4**. KEGG pathways associated with thermal stress within commercial broilers

**Supplementary Table S5**. KEGG pathways associated with thermal stress within Thai native chickens

**Supplementary Table S6**. KEGG pathways associated with thermal stress within crossbred H75
