# Supplementary material for: Thermal impacts on transcriptome of Pectoralis major muscle collected from commercial broilers, Thai native chickens and its crossbreeds
Source: Anim Biosci. 2023 Oct 31;37(1):61–73. doi: 10.5713/ab.23.0195 (PMC10766454; doi:10.5713/ab.23.0195)

Supplementary Figure S1 Primers (a) and scatter plots (b) from qPCR analysis for confirmation of RNA-Seq results

(A) Primers

NCBI Accession number	Gene ID	Gene Annotation	Sequence (5' ⟶ 3')	Amplicon length (bp)	Melting temperatu (°C)
AJ298335.1/	*CTGF*	Connective tissue growth	F: GCATGTTTGCTGACACAGGTT	145	62.48
AF463517.1		factor	R: TTGCTGACCAAGCCATGTGA		62.85
NM_204290.1	*FABP4*	Fatty acid binding protein 4	F: TATGAAAGAGCTGGGTGTGG	168	59.87
			R: GCTGTGGTCTCATCAAACTC		59.02
NM_204305.2	*GAPDH*	Glyceraldehyde-3-	F: ACTTTGGCATTGTGGAGGGT	131	62.53
		phosphate dehydrogenase	R: GGACGCTGGGATGATGTTCT		62.47
NM_204177.2	*LDHB*	Lactate dehydrogenase B	F: ACAGCGAGAACTGGAAGGAA	109	61.61
			R: AGCTCAGCAACGCTAAGACC		63.00
NM_204890.1	*SCD*	Stearoyl-CoA desaturase	F: GGCTGACAAAGTGGTGATG	137	59.20
			R: GGATGGCTGGAATGAAGA		58.69
NM_001005431.1	*FKBP5*	Peptidyl-prolyl cis-trans	F: GCTGGTGTACGAGGTCA	157	58.61
		isomerase fkbp5	R: CCCATACTGAATCACTGCCT		59.65
NM_205284.2	*LDHA*	lactate dehydrogenase A	F: TTCTCTGCCAGCTGAATAGCT	199	59.40
			R: CGGGTCATTGTCTTGTTGCAT		59.40
NM_001007831.2	*PHKB*	phosphorylase kinase	F: GCACGGTGTAGTAATTGTTGC	148	59.10
		regulatory subunit beta	R: GGGCACTTTGTGTCTCTAATG		59.40
NM_001109785.1	*HSP90*	Heat shock protein HSP 90-	F: GAGGATTATGAAGGCACAGG	198	58.21
		alpha	R: CTAAAGCCAGAGGACAGGAGR		59.65

(B) Scatter plots illustrates the comparisons between fold changes obtained from RNA-Seq and qPCR. *GAPDH* was used as reference genes.

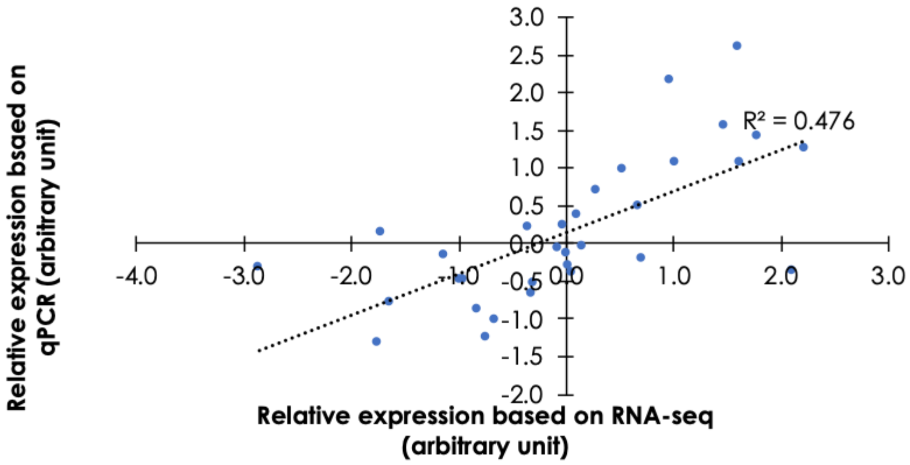

Supplement: Supplementary file 1 [file ab-23-0195-Supplementary-Fig-1.pdf]
