# Supplementary material for: Thermal impacts on transcriptome of Pectoralis major muscle collected from commercial broilers, Thai native chickens and its crossbreeds
Source: Anim Biosci. 2023 Oct 31;37(1):61–73. doi: 10.5713/ab.23.0195 (PMC10766454; doi:10.5713/ab.23.0195)
Supplement: Supplementary file 2 [file ab-23-0195-Supplementary-Table-1.pdf]

Table S1 Differentially expressed transcripts (DETs) associated with thermal stress in *P.major* muscle of commercial broilers
Only annotated transcripts (from 323 DETs) are presented.

NCBI Accession number	Gene description	log2FoldChange	P-value	KEGG Orthology ID
XP_040559305	myosin light chain 1, skeletal muscle isoform isoform X1	-15.03	8.43E-05	K05738
XP_015149240	HLA class II histocompatibility antigen gamma chain isoform X1	-5.48	1.11E-03	K06505
XP_015149240	HLA class II histocompatibility antigen gamma chain isoform X1	-4.21	8.40E-04	N/A
XP_046785444	myosin, heavy chain 1G, skeletal muscle (human myosin, heavy chain 1, skeletal muscle, adult) isoform X1	-3.71	4.53E-04	N/A
NP_990450	alpha-enolase isoform X4	-3.64	5.59E-04	N/A
XP_030904866	troponin T, fast skeletal muscle	-3.61	5.71E-04	N/A
NP_990615	L-lactate dehydrogenase A chain isoform X1	-3.41	1.63E-03	K00016
NP_990615	L-lactate dehydrogenase A chain isoform X1	-3.08	1.03E-03	K00016
NP_990615	L-lactate dehydrogenase A chain isoform X1	-2.86	2.21E-03	N/A
NP_990615	L-lactate dehydrogenase A chain isoform X1	-2.81	3.40E-03	N/A
NP_990615	L-lactate dehydrogenase A chain isoform X1	-2.64	1.07E-03	N/A
NP_990615	L-lactate dehydrogenase A chain isoform X1	-2.64	4.39E-03	N/A
NP_990450	alpha-enolase isoform X3	-2.50	4.84E-04	K01689
XP_046783746	START domain-containing protein 10	-2.46	1.72E-03	N/A
XP_429201	aminopeptidase Q isoform X2	-2.43	8.88E-05	K13724
NP_001385102	midkine isoform 2	-2.40	1.62E-04	N/A
NP_989554	ATP-dependent 6-phosphofructokinase, muscle type isoform X1	-2.36	6.36E-04	N/A
XP_425240	complement C1q tumor necrosis factor-related protein 8	-2.35	3.52E-02	K19470
XP_030904866	troponin T, fast skeletal muscle	-2.33	4.48E-03	N/A
NP_001383608	glial fibrillary acidic protein	-2.25	3.61E-02	K07610
XP_015140228	collagen alpha-1(XII) chain isoform X1	-2.25	8.81E-03	K08132
NP_990615	L-lactate dehydrogenase A chain isoform X1	-2.23	3.65E-03	N/A
XP_429539	uncharacterized protein LOC417973 isoform X1	-2.17	2.06E-02	N/A
NP_001384222	major histocompatibility complex class I antigen BF2 isoform X1	-2.15	1.07E-02	N/A
NP_001289116	sarcolipin isoform X1	-2.05	3.15E-03	N/A
NP_990221	stearoyl-CoA desaturase	-2.04	7.47E-04	K00507
NP_001384254	cartilage oligomeric matrix protein	-2.01	2.39E-02	K04659
XP_416113	40S ribosomal protein S16	-1.98	3.37E-02	N/A
XP_426381	myogenesis-regulating glycosidase isoform X2	-1.95	9.69E-04	N/A
XP_040552767	collagen alpha-1(XII) chain isoform X1	-1.93	3.19E-02	N/A
NP_990615	L-lactate dehydrogenase A chain isoform X1	-1.91	8.17E-03	N/A
XP_015142059	troponin T, fast skeletal muscle isoforms isoform X33	-1.91	1.60E-02	N/A
XP_040524301	scavenger receptor class A member 5 isoform X1	-1.91	1.99E-02	N/A
NP_001013414	myosin, heavy chain 1G, skeletal muscle (human myosin, heavy chain 1, skeletal muscle, adult) isoform X1	-1.89	4.49E-02	N/A
XP_015142059	troponin T, fast skeletal muscle isoforms isoform X33	-1.87	1.49E-02	N/A
XP_429539	uncharacterized protein LOC417973 isoform X1	-1.85	4.72E-02	N/A
XP_003643029	C-C motif chemokine 26	-1.84	2.18E-02	N/A
NP_990615	L-lactate dehydrogenase A chain isoform X1	-1.83	1.56E-02	K00016
NP_990615	L-lactate dehydrogenase A chain isoform X1	-1.82	1.10E-02	K00016
NP_001383050	slit homolog 3 protein isoform X2	-1.81	3.22E-02	K06850
NP_990615	L-lactate dehydrogenase A chain isoform X1	-1.80	1.21E-02	K00016
XP_040551816	transmembrane protein 108 isoform X1	-1.80	1.31E-02	N/A
NP_001305944	myosin, heavy chain 1G, skeletal muscle (human myosin, heavy chain 1, skeletal muscle, adult) isoform X1	-1.79	2.82E-02	N/A
XP_046800474	phosphoglucomutase-1 isoform X2	-1.78	3.08E-03	N/A
XP_046785444	myosin, heavy chain 1G, skeletal muscle (human myosin, heavy chain 1, skeletal muscle, adult) isoform X1	-1.78	3.28E-03	N/A
NP_990615	L-lactate dehydrogenase A chain isoform X1	-1.77	1.42E-02	K00016
NP_001382957	girdin isoform X2	-1.76	7.21E-03	N/A
XP_025005496	AP-1 complex subunit sigma-2 isoform X1	-1.73	2.61E-02	N/A
NP_001382957	neurofilament light polypeptide isoform X2	-1.69	1.22E-02	K07609
XP_015142059	troponin T, fast skeletal muscle isoforms isoform X33	-1.69	1.72E-02	N/A
XP_015142059	troponin T, fast skeletal muscle isoforms isoform X33	-1.67	1.56E-02	N/A
XP_426381	myogenesis-regulating glycosidase isoform X2	-1.65	4.28E-03	N/A
XP_040511818	uncharacterized protein LOC101752072	-1.64	3.00E-02	N/A
XP_015142059	troponin T, fast skeletal muscle isoforms isoform X33	-1.63	2.50E-02	N/A
XP_046797375	troponin T, fast skeletal muscle isoforms isoform X3	-1.60	9.89E-05	N/A
XP_040547722	telethonin	-1.59	9.52E-04	K19879
XP_046797375	troponin T, fast skeletal muscle isoforms isoform X3	-1.57	2.05E-03	N/A
NP_989559	myosin, heavy chain 1E, skeletal muscle isoform X1	-1.57	3.36E-02	N/A
NP_001313425	fibulin-1 isoform X1	-1.55	4.17E-02	K17342
XP_040560931	E-selectin isoform X1	-1.54	3.43E-02	K06494

Table S1 Cont.

NCBI Accession number	Gene description	log2FoldChange	P-value	KEGG Orthology ID
NP_001277130	protein CASP	-1.54	2.40E-04	K09313
NP_001277130	protein CASP	-1.53	1.10E-04	K09313
XP_046797375	troponin T, fast skeletal muscle isoforms isoform X34	-1.53	8.37E-03	N/A
NP_001383608	glial fibrillary acidic protein	-1.52	1.66E-02	N/A
XP_015139877	centriole, cilia and spindle-associated protein	-1.51	9.85E-03	K16454
XP_015135025	ras-related protein Rab-39A	-1.50	4.55E-02	K07924
XP_046797375	troponin T, fast skeletal muscle isoforms isoform X3	-1.49	1.84E-03	N/A
XP_046797375	troponin T, fast skeletal muscle isoforms isoform X34	-1.49	1.34E-03	N/A
XP_046797388	troponin T, fast skeletal muscle isoforms isoform X31	-1.48	5.07E-03	K12046
XP_040559444	tumor necrosis factor-inducible gene 6 protein isoform X3	-1.47	5.57E-03	K19018
NP_001073681	ubiquitin carboxyl-terminal hydrolase isozyme L3 isoform X1	-1.46	2.72E-03	N/A
XP_046785444	myosin, heavy chain 1G, skeletal muscle (human myosin, heavy chain 1, skeletal muscle, adult) isoform X1	-1.46	3.83E-04	N/A
NP_990847	calponin-2 isoform X1	-1.45	2.44E-02	N/A
NP_990648	carbonic anhydrase 2 isoform X1	-1.44	7.64E-04	K18245
XP_040532604	carbonic anhydrase 2 isoform X1	-1.42	1.12E-03	N/A
NP_990615	L-lactate dehydrogenase A chain isoform X1	-1.41	2.91E-02	K00016
NP_990450	alpha-enolase isoform X3	-1.41	5.97E-03	K01689
NP_001156704	glutathione peroxidase 3 precursor	-1.40	4.76E-02	N/A
NP_001185660	TP53-target gene 5 protein isoform X3	-1.39	3.51E-02	N/A
NP_001304756	aquaporin-2 isoform X1	-1.38	2.12E-02	K09866
XP_015154188	troponin T, cardiac muscle isoforms isoform X3	-1.38	1.50E-02	K12045
XP_046797375	troponin T, fast skeletal muscle isoforms isoform X3	-1.38	1.48E-02	N/A
NP_001107181	myosin, heavy chain 1G, skeletal muscle (human myosin, heavy chain 1, skeletal muscle, adult) isoform X1	-1.37	4.61E-02	N/A
XP_046773807	troponin T, fast skeletal muscle isoforms isoform X34	-1.36	3.19E-03	K12046
NP_990615	L-lactate dehydrogenase A chain isoform X1	-1.35	3.93E-02	K00016
XP_015147703	integrin alpha-11 isoform X4	-1.33	1.82E-02	K06587
NP_990615	L-lactate dehydrogenase A chain isoform X1	-1.33	4.94E-03	K00016
NP_001264664	signal transducer CD24 precursor	-1.33	2.25E-02	N/A
XP_046773807	troponin T, fast skeletal muscle isoforms isoform X34	-1.33	1.12E-03	N/A
NP_001382957	nestin	-1.32	3.15E-02	N/A
XP_046797375	troponin T, fast skeletal muscle isoforms isoform X34	-1.32	1.16E-02	N/A
XP_046797375	troponin T, fast skeletal muscle isoforms isoform X34	-1.32	1.83E-03	N/A
XP_015154188	troponin T, cardiac muscle isoforms isoform X3	-1.32	1.96E-02	K12045
XP_046797375	troponin T, fast skeletal muscle isoforms isoform X3	-1.31	1.18E-03	N/A
NP_001001604	sia-alpha-2,3-Gal-beta-1,4-GlcNAc-R:alpha 2,8-sialyltransferase	-1.31	3.19E-02	K06612
NP_001305915	fibronectin type III domain-containing protein 5 precursor	-1.30	1.16E-02	K24486
XP_046782844	HLA class II histocompatibility antigen gamma chain isoform X1	-1.29	2.47E-02	K06505
NP_001004412	membrane metallo-endopeptidase-like 1 isoform X3	-1.29	9.69E-03	K01389
XP_046797375	troponin T, fast skeletal muscle isoforms isoform X3	-1.29	9.52E-03	N/A
NP_001107181	myosin, heavy chain 1E, skeletal muscle isoform X1	-1.29	3.46E-02	N/A
XP_040556620	troponin T, fast skeletal muscle isoforms isoform X31	-1.27	2.58E-04	N/A
XP_001232083	protein FAM102A isoform X1	-1.25	1.69E-02	N/A
XP_017694930	synaptophysin-like protein 2	-1.24	5.04E-03	N/A
NP_001034694	5'-AMP-activated protein kinase catalytic subunit alpha-2	-1.24	2.29E-03	N/A
NP_990450	alpha-enolase isoform X3	-1.23	4.07E-02	N/A
XP_040544196	puratrophin-1 isoform X4	-1.23	1.11E-02	N/A
NP_001383353	insulin-induced gene 1 protein	-1.20	3.04E-02	N/A
XP_001232083	protein FAM102A isoform X1	-1.20	2.28E-02	N/A
XP_046773807	troponin T, fast skeletal muscle isoforms isoform X34	-1.20	5.34E-04	N/A
XP_046797375	troponin T, fast skeletal muscle isoforms isoform X3	-1.19	4.57E-03	N/A
NP_990615	L-lactate dehydrogenase A chain isoform X1	-1.16	4.92E-02	N/A
XP_046797375	troponin T, fast skeletal muscle isoforms isoform X3	-1.15	1.62E-03	N/A
NP_001384704	cytochrome b-245 beta chain isoform 3	-1.15	2.90E-02	K21421
NP_001385076	CMRF35-like molecule 8 isoform X1	-1.15	2.41E-02	N/A
XP_040522142	death domain-containing membrane protein NRADD-like isoform X3	-1.15	1.02E-02	K02583
NP_001384338	myosin, heavy chain 1E, skeletal muscle isoform X1	-1.15	1.89E-02	N/A
XP_001232083	protein FAM102A isoform X1	-1.14	1.89E-02	N/A
XP_015142062	troponin T, fast skeletal muscle isoforms isoform X34	-1.14	5.42E-03	N/A
NP_001244215	transcription elongation factor SPT4	-1.14	3.58E-03	N/A
XP_024998676	phosphatidylethanolamine-binding protein 4	-1.14	7.09E-03	N/A
XP_015142062	troponin T, fast skeletal muscle isoforms isoform X34	-1.13	5.89E-03	N/A
NP_990512	stathmin-3 isoform X1	-1.13	4.54E-02	N/A

Table S1 Cont.

NCBI Accession number	Gene description	log2FoldChange	P-value	KEGG Orthology ID
XP_015142062	troponin T, fast skeletal muscle isoforms isoform X34	-1.13	5.57E-04	K12046
XP_046797375	troponin T, fast skeletal muscle isoforms isoform X34	-1.13	4.35E-03	N/A
XP_040550182	NADH-ubiquinone oxidoreductase chain 5 isoform X2	-1.13	4.11E-03	N/A
NP_001376368	serine-rich and transmembrane domain-containing protein 1	-1.13	1.90E-02	N/A
XP_420639	arylsulfatase J isoform X1	-1.13	6.53E-03	K12375
XP_046797375	troponin T, fast skeletal muscle isoforms isoform X3	-1.12	6.09E-04	K12046
XP_413930	integrin alpha-11 isoform X4	-1.12	3.40E-02	K06587
XP_424573	glutamine--fructose-6-phosphate aminotransferase [isomerizing] 2 isoform X2	-1.12	3.92E-02	K00820
NP_990450	alpha-enolase isoform X3	-1.11	3.27E-02	N/A
NP_990450	alpha-enolase isoform X3	-1.10	3.54E-02	N/A
XP_046797375	troponin T, fast skeletal muscle isoforms isoform X3	-1.10	2.56E-03	K12046
XP_015146763	mucin-17	-1.09	7.77E-03	N/A
NP_990615	L-lactate dehydrogenase A chain isoform X1	-1.08	3.30E-02	N/A
NP_001306233	myosin, heavy chain 1G, skeletal muscle (human myosin, heavy chain 1, skeletal muscle, adult) isoform X1	-1.08	2.76E-02	N/A
NP_001026727	bisphosphoglycerate mutase isoform X1	-1.07	2.96E-02	K01834
NP_001007081	extracellular matrix organizing protein FRAS1 isoform X3	-1.06	1.95E-02	K24517
XP_009635596	insulin-like growth factor II	-1.06	4.30E-02	N/A
XP_046780429	repulsive guidance molecule A isoform X1	-1.05	3.71E-02	K23096
XP_046797388	troponin T, fast skeletal muscle isoforms isoform X31	-1.05	1.80E-02	N/A
XP_025001366	putative nuclease HARBI1	1.05	2.98E-02	N/A
XP_046755994	Krueppel-like factor 15 isoform X1	1.05	4.96E-02	K09210
NP_001034690	aldehyde oxidase isoform X2	1.06	7.93E-03	K00157
XP_015132285	period circadian protein homolog 2 isoform X1	1.07	8.12E-03	K02633
XP_419887	succinate dehydrogenase assembly factor 4, mitochondrial isoform X1	1.08	5.63E-03	N/A
XP_046759061	serine/threonine-protein kinase SIK2 isoform X1	1.08	1.24E-02	N/A
XP_040537315	dual specificity protein phosphatase 22-A isoform X1	1.09	1.94E-03	N/A
XP_416732	SH3 domain-binding glutamic acid-rich-like protein isoform X2	1.09	3.74E-02	K23740
XP_009577091	cAMP-specific 3',5'-cyclic phosphodiesterase 4D-like	1.09	2.99E-02	N/A
XP_001231519	phospholipase A2 group XV	1.10	5.98E-04	K06129
XP_004936089	leucine-rich repeat-containing protein 66 isoform X1	1.10	4.79E-02	N/A
XP_419796	sestrin-1 isoform X2	1.11	1.10E-02	K10141
XP_015149053	teneurin-2 isoform X1	1.13	1.32E-03	K24473
XP_015155923	uncharacterized protein LOC107055438	1.14	7.31E-03	N/A
XP_046757901	translation initiation factor IF-2-like isoform X2	1.15	3.35E-02	N/A
XP_003641087	ectonucleotide pyrophosphatase/phosphodiesterase family member 3	1.15	1.29E-02	K01513
XP_003641087	ectonucleotide pyrophosphatase/phosphodiesterase family member 3	1.15	6.12E-03	K01513
NP_990850	sarcoplasmic/endoplasmic reticulum calcium ATPase 2 isoform X1	1.16	4.81E-02	N/A
XP_040537311	uncharacterized protein DUSP22AL isoform X7	1.19	1.67E-02	N/A
XP_041272923	uncharacterized protein LOC121342498	1.19	3.69E-02	N/A
XP_040504783	leukocyte receptor cluster member 9-like	1.22	1.05E-02	N/A
NP_001193425	fructose-bisphosphate aldolase A	1.22	3.37E-02	K01623
XP_004940389	sestrin-1 isoform X9	1.24	7.55E-03	K10141
NP_001264183	carboxypeptidase N catalytic chain isoform X1	1.24	4.73E-02	K01292
NP_001026341	ribose-5-phosphate isomerase	1.25	1.86E-03	K01807
NP_990850	sarcoplasmic/endoplasmic reticulum calcium ATPase 3 isoform X1	1.27	2.97E-02	N/A
NP_001072961	60S ribosomal protein L37a	1.28	4.35E-02	N/A
XP_040537314	dual specificity protein phosphatase 22-A isoform X1	1.29	2.25E-03	N/A
XP_040557740	uncharacterized protein LOC121113269	1.30	4.20E-02	N/A
XP_015128495	RNA-binding protein 4B-like	1.31	2.23E-03	N/A
XP_009577091	cAMP-specific 3',5'-cyclic phosphodiesterase 4D-like	1.31	1.86E-03	N/A
XP_025001427	protein ZNRD2 isoform X1	1.32	1.55E-02	N/A
NP_001185673	myosin regulatory light chain 2B, cardiac muscle isoform isoform X1	1.32	5.81E-03	N/A
NP_990511	fibroblast growth factor 1 isoform X1	1.33	1.04E-02	N/A
XP_015138133	DNA repair-scaffolding protein isoform X1	1.33	1.19E-02	K22806
NP_001072949	actin, aortic smooth muscle isoform X1	1.33	4.03E-02	K12313
XP_423026	GTP-binding protein Di-Ras2	1.34	2.38E-02	K07841
NP_001026400	actin, aortic smooth muscle isoform X1	1.34	4.25E-02	N/A
XP_423026	GTP-binding protein Di-Ras2	1.35	5.23E-03	K07841
NP_990450	alpha-enolase isoform X3	1.35	2.36E-02	N/A

NP_001193425	fructose-bisphosphate aldolase A	1.36	1.97E-02	N/A
XP_040537316	uncharacterized protein DUSP22AL isoform X11	1.40	1.60E-03	N/A

Table S1 Cont.

NCBI Accession number	Gene description	log2FoldChange	P-value	KEGG Orthology ID
NP_990149	glutathione S-transferase alpha 4 isoform X1	1.41	7.09E-03	K00799
XP_040551279	DNA polymerase delta catalytic subunit	1.42	2.14E-02	N/A
XP_423026	GTP-binding protein Di-Ras2	1.45	6.41E-03	N/A
XP_015142059	troponin T, fast skeletal muscle isoforms isoform X33	1.48	3.97E-02	N/A
NP_001264279	CUE domain-containing protein 2 isoform X4	1.48	4.14E-03	N/A
NP_001072961	60S ribosomal protein L37a	1.53	2.34E-02	N/A
XP_040539581	mapk-regulated corepressor-interacting protein 1 isoform X1	1.55	2.11E-03	N/A
NP_990838	creatine kinase B-type isoform X1	1.58	3.82E-02	N/A
XP_040502958	nascent polypeptide-associated complex subunit alpha, muscle-specific form-like	1.59	2.80E-03	N/A
NP_989908	aldehyde dehydrogenase 1A1 isoform X1	1.61	1.52E-02	K07249
XP_041272923	uncharacterized protein LOC121342498	1.62	3.20E-02	N/A
NP_990838	creatine kinase B-type isoform X1	1.62	1.74E-02	N/A
NP_990503	actin, aortic smooth muscle isoform X1	1.62	3.57E-02	N/A
XP_040524394	L-threonine 3-dehydrogenase, mitochondrial	1.67	1.86E-02	K15789
NP_001186387	synaptophysin-like protein 2 isoform X2	1.69	6.15E-03	N/A
NP_001005431	peptidyl-prolyl cis-trans isomerase FKBP5 isoform X1	1.71	7.38E-03	K09571
NP_990450	alpha-enolase isoform X3	1.73	6.12E-03	N/A
NP_001005431	peptidyl-prolyl cis-trans isomerase FKBP5 isoform X1	1.79	9.64E-03	K09571
NP_001005431	peptidyl-prolyl cis-trans isomerase FKBP5 isoform X1	1.85	8.51E-03	N/A
XP_010722398	peptidyl-prolyl cis-trans isomerase FKBP5 isoform X1	1.87	2.14E-02	N/A
XP_025002525	RNA-binding protein 33 isoform X6	1.90	5.81E-03	N/A
XP_010722398	peptidyl-prolyl cis-trans isomerase FKBP5 isoform X1	1.94	1.68E-02	N/A
XP_015134822	adenylate kinase isoenzyme 1 isoform X2	2.07	9.53E-04	N/A
XP_040524394	L-threonine 3-dehydrogenase, mitochondrial	2.10	4.43E-03	K15789
XP_015156382	cystathionine beta-synthase isoform X1	2.15	1.66E-02	K01697
NP_001161216	creatine kinase B-type isoform X1	2.16	1.46E-02	K00933
XP_040524394	L-threonine 3-dehydrogenase, mitochondrial	2.20	2.43E-03	K15789
XP_046797375	troponin T, fast skeletal muscle isoforms isoform X3	2.72	8.95E-05	K12046
XP_046797375	troponin T, fast skeletal muscle isoforms isoform X34	2.72	2.30E-06	K12046
XP_046797375	troponin T, fast skeletal muscle isoforms isoform X34	2.82	1.32E-06	K12046
XP_015150480	major histocompatibility complex class II beta chain BLB1, (HLA class II, D beta chain) isoform X1	3.14	3.73E-02	K06752
XP_046771482	phosphoglycerate kinase isoform X1	3.32	2.46E-03	N/A
NP_990748	troponin I, slow skeletal muscle isoform X1	3.74	3.11E-03	K12043
XP_030904866	troponin T, fast skeletal muscle	3.92	2.12E-03	N/A
XP_015142062	troponin T, fast skeletal muscle isoforms isoform X34	4.06	1.11E-07	K12046
XP_040512781	fructose-bisphosphate aldolase A	4.08	9.37E-03	K01623
XP_046797375	troponin T, fast skeletal muscle isoforms isoform X3	4.78	2.13E-05	N/A
XP_046797375	troponin T, fast skeletal muscle isoforms isoform X34	7.21	2.36E-08	N/A

N/A = not applicable
Color code presented in colume E

- DETs found only in BR
- DETs found in all breeds
- DETs found in BR and NT
- DETs found in BR and H75
