# Supplementary material for: Thermal impacts on transcriptome of Pectoralis major muscle collected from commercial broilers, Thai native chickens and its crossbreeds
Source: Anim Biosci. 2023 Oct 31;37(1):61–73. doi: 10.5713/ab.23.0195 (PMC10766454; doi:10.5713/ab.23.0195)
Supplement: Supplementary file 3 [file ab-23-0195-Supplementary-Table-2.pdf]

Table S2 Differentially expressed transcripts (DETs) associated with thermal stress in *P. major* muscle of Thai Native chicken
Only annotated transcripts (from 439 DETs) are presented.

NCBI Accession number	Gene description	log2FoldChange	P-value	KEGG Orthology ID
XP_015148734	leiomodin-3	-5.79	4.60E-06	N/A
XP_025011798	adenylate kinase isoenzyme 1 isoform X2	-5.50	3.05E-04	K00939
NP_990748	troponin I, slow skeletal muscle isoform X1	-4.58	3.22E-04	K12043
NP_990615	L-lactate dehydrogenase A chain isoform X1	-4.07	1.82E-04	K00016
NP_001038097	myosin light chain 1, skeletal muscle isoform isoform X1	-3.72	3.14E-06	N/A
XP_025011798	adenylate kinase isoenzyme 1 isoform X2	-3.71	5.11E-03	K00939
NP_990748	troponin I, slow skeletal muscle isoform X1	-3.42	8.57E-03	K12043
XP_024997750	inactive serine/threonine-protein kinase TEX14 isoform X1	-3.23	7.56E-03	N/A
NP_990782	triosephosphate isomerase	-3.03	6.01E-04	N/A
NP_001032920	tyrosine-protein phosphatase non-receptor type substrate 1 isoform X1	-2.96	6.45E-03	N/A
NP_990748	troponin I, slow skeletal muscle isoform X1	-2.91	1.25E-03	N/A
XP_046784130	Ig lambda chain V-1 region isoform X34	-2.74	3.45E-03	N/A
NP_990298	keratocan isoform X1	-2.67	4.83E-03	K08121
NP_001313425	fibulin-1 isoform X1	-2.65	5.52E-04	K17342
NP_990615	L-lactate dehydrogenase A chain isoform X1	-2.48	6.36E-03	K00016
NP_996868	leukocyte cell-derived chemotaxin 1	-2.44	1.40E-03	N/A
NP_990615	L-lactate dehydrogenase A chain isoform X1	-2.33	6.07E-03	K00016
XP_425240	complement C1q tumor necrosis factor-related protein 8	-2.29	4.08E-02	K19470
NP_990748	troponin I, slow skeletal muscle isoform X1	-2.26	2.35E-03	K12043
NP_001026160	SH3 domain-binding protein 5	-2.22	5.24E-03	K23739
NP_990748	troponin I, slow skeletal muscle isoform X1	-2.21	2.09E-03	N/A
XP_015140228	collagen alpha-1(XII) chain isoform X1	-2.17	1.18E-02	K08132
NP_990729	translationally-controlled tumor protein homolog	-2.12	9.05E-04	N/A
NP_990729	translationally-controlled tumor protein homolog	-2.08	4.89E-03	N/A
NP_990729	translationally-controlled tumor protein homolog	-2.06	6.31E-03	N/A
NP_990063	melanoma inhibitory activity protein 2 isoform X1	-1.92	8.57E-04	N/A
XP_040552767	collagen alpha-1(XII) chain isoform X1	-1.82	4.39E-02	N/A
YP_009555267	cytochrome c oxidase subunit III (mitochondrion)	-1.81	4.53E-02	N/A
XP_025011798	adenylate kinase isoenzyme 1 isoform X2	-1.79	3.74E-02	K00939
NP_001280103	polyubiquitin-B isoform X1	-1.78	3.26E-02	K04551
NP_990782	triosephosphate isomerase	-1.75	3.88E-03	K01803
XP_001231316	podoplanin isoform X2	-1.67	3.92E-03	K16778
YP_009555263	cytochrome c oxidase subunit I (mitochondrion)	-1.66	4.18E-02	N/A
YP_009555261	NADH dehydrogenase subunit 1 (mitochondrion)	-1.62	1.74E-02	N/A
XP_424816	very-long-chain (3R)-3-hydroxyacyl-CoA dehydratase 4	-1.62	3.67E-03	K10703
XP_414400	mitochondrial carnitine/acylcarnitine carrier protein isoform X1	-1.61	4.87E-03	N/A
NP_990482	glial fibrillary acidic protein	-1.60	2.42E-03	K10378
XP_015131875	protocadherin Fat 4	-1.54	1.13E-02	K16669
XP_040550182	NADH-ubiquinone oxidoreductase chain 5 isoform X2	-1.54	9.66E-03	N/A
YP_009555264	cytochrome c oxidase subunit II (mitochondrion)	-1.53	1.46E-02	N/A
NP_990748	troponin I, slow skeletal muscle isoform X1	-1.53	1.34E-02	K12043
XP_040559305	myosin light chain 1, skeletal muscle isoform isoform X1	-1.53	7.91E-06	N/A
NP_001264664	signal transducer CD24 precursor	-1.51	9.41E-03	N/A
NP_990450	alpha-enolase isoform X3	-1.51	2.73E-02	N/A
NP_001280103	polyubiquitin-B isoform X1	-1.50	4.74E-02	N/A
NP_001038097	myosin light chain 1, skeletal muscle isoform isoform X1	-1.49	1.33E-02	N/A
NP_001038097	myosin light chain 1, skeletal muscle isoform isoform X1	-1.47	4.18E-03	N/A
NP_990450	alpha-enolase isoform X3	-1.47	4.68E-02	N/A
NP_990748	troponin I, slow skeletal muscle isoform X1	-1.43	1.23E-02	N/A
NP_001280103	polyubiquitin-B isoform X1	-1.43	2.16E-02	N/A
NP_001385203	actin, aortic smooth muscle isoform X1	-1.43	2.34E-02	K10354
XP_417405	40S ribosomal protein S21	-1.42	4.51E-02	K02971
XP_015142059	troponin T, fast skeletal muscle isoforms isoform X33	-1.42	2.01E-02	N/A
YP_009555267	cytochrome c oxidase subunit III (mitochondrion)	-1.42	4.40E-02	N/A
XP_040559305	myosin light chain 1, skeletal muscle isoform isoform X1	-1.40	1.68E-03	K05738
NP_001038097	myosin light chain 1, skeletal muscle isoform isoform X1	-1.40	3.88E-04	N/A
NP_001280103	polyubiquitin-B isoform X1	-1.40	1.74E-03	N/A
XP_015142059	troponin T, fast skeletal muscle isoforms isoform X33	-1.39	1.51E-02	N/A
XP_015142059	troponin T, fast skeletal muscle isoforms isoform X33	-1.38	2.77E-02	N/A
XP_015142059	troponin T, fast skeletal muscle isoforms isoform X33	-1.37	2.45E-02	N/A
NP_001038097	myosin light chain 1, skeletal muscle isoform isoform X1	-1.36	1.49E-03	K05738
XP_040550182	NADH-ubiquinone oxidoreductase chain 5 isoform X2	-1.36	2.07E-02	N/A
NP_990450	alpha-enolase isoform X3	-1.35	1.34E-02	N/A
XP_046799681	titin isoform X1	-1.35	1.73E-02	K12567
XP_040559305	myosin light chain 1, skeletal muscle isoform isoform X1	-1.35	1.61E-04	K05738

Table S2 Cont.

NCBI Accession number	Gene description	log2FoldChange	P-value	KEGG Orthology ID
NP_001274134	polyubiquitin-B isoform X1	-1.34	4.81E-02	N/A
YP_009555270	NADH dehydrogenase subunit 4 (mitochondrion)	-1.32	4.55E-02	N/A
NP_990729	translationally-controlled tumor protein homolog	-1.32	3.20E-03	N/A
XP_046793727	collagen alpha-1(I) chain-like	-1.31	1.59E-03	N/A
YP_009555264	cytochrome c oxidase subunit II (mitochondrion)	-1.31	4.71E-02	N/A
XP_046800279	glutamine synthetase isoform X2	-1.30	1.68E-02	K01915
NP_990464	troponin C, slow skeletal and cardiac muscles	-1.30	4.71E-02	N/A
NP_001038097	myosin light chain 1, skeletal muscle isoform isoform X1	-1.29	1.13E-02	K05738
XP_046777675	immunoglobulin-like and fibronectin type III domain-containing protein 1	-1.29	2.10E-02	K12567
XP_046757526	translation initiation factor IF-2-like	-1.29	1.42E-03	N/A
NP_001038097	myosin light chain 1, skeletal muscle isoform isoform X1	-1.28	4.71E-04	N/A
XP_040559305	myosin light chain 1, skeletal muscle isoform isoform X1	-1.27	4.50E-03	K05738
XP_046793727	collagen alpha-1(I) chain-like	-1.25	2.00E-03	N/A
XP_046793727	collagen alpha-1(I) chain-like	-1.25	3.34E-03	N/A
XP_046793727	collagen alpha-1(I) chain-like	-1.25	1.10E-02	N/A
NP_990450	alpha-enolase isoform X3	-1.25	3.63E-02	N/A
XP_040559305	myosin light chain 1, skeletal muscle isoform isoform X1	-1.24	4.79E-03	K05738
XP_046797375	troponin T, fast skeletal muscle isoforms isoform X3	-1.22	3.64E-02	K12046
NP_990482	glial fibrillary acidic protein	-1.21	3.55E-02	K10378
XP_025003973	elongation factor 1-delta isoform X8	-1.20	2.87E-02	N/A
NP_001385203	actin, aortic smooth muscle isoform X1	-1.19	3.74E-02	K10354
XP_032563740	uncharacterized protein LOC116796865	-1.18	3.71E-03	N/A
NP_001038097	myosin light chain 1, skeletal muscle isoform isoform X1	-1.18	4.30E-03	N/A
XP_046793727	collagen alpha-1(I) chain-like	-1.17	2.79E-03	N/A
YP_009555264	cytochrome c oxidase subunit II (mitochondrion)	-1.17	4.81E-02	N/A
NP_001280103	polyubiquitin-B isoform X1	-1.17	2.56E-02	N/A
XP_025011798	adenylate kinase isoenzyme 1 isoform X2	-1.17	4.74E-02	N/A
NP_990748	troponin I, slow skeletal muscle isoform X1	-1.17	2.66E-02	K12043
XP_040519214	carboxymethylenebutenolidase homolog	-1.17	3.45E-02	K01061
XP_041272923	ankyrin repeat domain-containing protein 7-like	-1.17	1.23E-03	N/A
NP_001038097	myosin light chain 1, skeletal muscle isoform isoform X1	-1.16	1.91E-03	K05738
XP_032563740	uncharacterized protein LOC116796865	-1.16	6.68E-03	N/A
XP_040556984	serine/threonine-protein kinase VRK1 isoform X1	-1.15	4.60E-02	N/A
NP_990464	calcium-binding mitochondrial carrier protein SCaMC-1	-1.14	1.82E-02	N/A
XP_004944672	mitochondrial potassium channel	-1.14	1.10E-03	N/A
NP_990450	alpha-enolase isoform X3	-1.13	2.11E-02	N/A
XP_040559305	myosin light chain 1, skeletal muscle isoform isoform X1	-1.12	4.63E-02	N/A
NP_001026727	bisphosphoglycerate mutase isoform X1	-1.12	2.06E-02	K01834
XP_040559305	myosin light chain 1, skeletal muscle isoform isoform X1	-1.12	7.47E-03	K05738
NP_001385203	actin, aortic smooth muscle isoform X1	-1.12	4.47E-02	K10354
XP_015142059	troponin T, fast skeletal muscle isoforms isoform X33	-1.12	4.51E-02	N/A
XP_032563740	uncharacterized protein LOC116796865	-1.11	1.10E-02	N/A
NP_990729	translationally-controlled tumor protein homolog	-1.11	3.65E-02	N/A
NP_001038097	myosin light chain 1, skeletal muscle isoform isoform X1	-1.11	2.05E-03	N/A
XP_015142059	troponin T, fast skeletal muscle isoforms isoform X33	-1.10	3.65E-02	N/A
NP_990729	translationally-controlled tumor protein homolog	-1.10	2.62E-02	N/A
XP_041272923	ankyrin repeat domain-containing protein 7-like	-1.10	5.27E-03	N/A
NP_990464	troponin C, slow skeletal and cardiac muscles	-1.10	4.84E-02	N/A
XP_032563740	uncharacterized protein LOC116796865	-1.10	1.25E-02	N/A
XP_015142059	troponin T, fast skeletal muscle isoforms isoform X33	-1.09	2.07E-02	N/A
XP_039246074	collagen alpha-1(I) chain-like	-1.09	6.92E-03	N/A
XP_041272922	translation initiation factor IF-2-like	-1.09	3.77E-04	N/A
XP_040541056	SEC14-like protein 2 isoform X2	-1.09	2.06E-02	K17981
NP_990450	alpha-enolase isoform X3	-1.08	3.99E-02	N/A
XP_046793727	collagen alpha-1(I) chain-like	-1.08	3.91E-03	N/A
XP_046777675	obscurin isoform X10	-1.08	3.81E-02	K12567
NP_001038097	myosin light chain 1, skeletal muscle isoform isoform X1	-1.08	1.04E-04	N/A
XP_041272923	guanylate cyclase soluble subunit beta-1-like	-1.08	9.23E-03	N/A
XP_040559305	myosin light chain 1, skeletal muscle isoform isoform X1	-1.08	1.45E-02	N/A
NP_990748	troponin I, slow skeletal muscle isoform X1	-1.07	2.82E-02	N/A
XP_046793727	collagen alpha-1(I) chain-like	-1.07	2.77E-03	N/A
NP_001038097	myosin light chain 1, skeletal muscle isoform isoform X1	-1.06	1.94E-03	N/A
XP_041272923	ankyrin repeat domain-containing protein 7-like	-1.05	3.73E-03	N/A
XP_046793727	collagen alpha-1(I) chain-like	-1.02	9.84E-03	N/A
NP_990609	myosin regulatory light chain 2, smooth muscle major isoform isoform X1	1.05	2.13E-02	K12755
NP_990615	L-lactate dehydrogenase A chain isoform X1	1.05	4.21E-03	K00016

Table S2 Cont.

NCBI Accession number	Gene description	log2FoldChange	P-value	KEGG Orthology ID
XP_046800084	thrombospondin type-1 domain-containing protein 7B isoform X1	1.06	1.33E-02	N/A
XP_426398	olfactomedin-like protein 1	1.06	1.12E-02	K25448
NP_990615	L-lactate dehydrogenase A chain isoform X1	1.06	1.93E-02	N/A
XP_046799475	collagen alpha-1(III) chain isoform X1	1.07	2.29E-02	N/A
XP_042732183	cellular retinoic acid-binding protein 1	1.07	1.43E-02	N/A
NP_990615	L-lactate dehydrogenase A chain isoform X1	1.08	1.97E-03	N/A
XP_416518	mannan-binding lectin serine protease 2 isoform X3	1.08	4.04E-02	K01330
XP_046800897	uncharacterized protein LOC124416949	1.09	3.73E-03	N/A
XP_015709241	homeobox protein Hox-A6 isoform X1	1.09	5.39E-03	N/A
NP_990615	L-lactate dehydrogenase A chain isoform X1	1.09	1.01E-02	N/A
NP_001034687	collectin-11 isoform X2	1.09	1.04E-02	K10065
XP_040558250	wings apart-like protein homolog isoform X1	1.10	2.21E-03	N/A
XP_046799475	collagen alpha-1(III) chain isoform X1	1.10	1.97E-02	N/A
XP_046800474	phosphoglucomutase-1 isoform X2	1.10	3.35E-02	N/A
XP_001231676	dermatopontin	1.10	1.09E-03	K25512
NP_990615	L-lactate dehydrogenase A chain isoform X1	1.10	2.60E-03	N/A
NP_990615	L-lactate dehydrogenase A chain isoform X1	1.11	9.90E-03	K00016
NP_001274134	polyubiquitin-B isoform X1	1.11	1.38E-02	N/A
YP_009555264	cytochrome c oxidase subunit II (mitochondrion)	1.11	3.43E-02	N/A
NP_990615	L-lactate dehydrogenase A chain isoform X1	1.11	1.53E-02	N/A
NP_990615	L-lactate dehydrogenase A chain isoform X1	1.12	1.64E-03	K00016
XP_040559305	myosin light chain 1, skeletal muscle isoform isoform X1	1.12	3.83E-03	N/A
NP_990615	L-lactate dehydrogenase A chain isoform X1	1.13	1.58E-02	N/A
XP_040554781	fibroblast growth factor 16 isoform X1	1.15	4.23E-02	K04358
NP_990615	L-lactate dehydrogenase A chain isoform X1	1.15	2.42E-03	K00016
NP_001008674	dynein axonemal assembly factor 4 isoform X1	1.15	1.29E-02	K19758
NP_990635	melanotransferrin isoform X1	1.15	1.94E-02	K14736
NP_990016	coagulation factor XIII A chain isoform X2	1.15	3.75E-02	K03917
XP_046765712	uncharacterized protein LOC124417654	1.16	1.38E-02	N/A
XP_418741	polypeptide N-acetylgalactosaminyltransferase 15 isoform X1	1.16	1.65E-02	K00710
NP_990690	insulin-like growth factor-binding protein 2 isoform X1	1.16	1.53E-02	K23575
XP_046757901	uncharacterized protein LOC124417285	1.18	1.15E-02	N/A
NP_989899	vascular endothelial growth factor D isoform X1	1.18	9.91E-04	K05449
XP_015133466	laminin subunit alpha-3 isoform X1	1.18	1.47E-03	K06240
XP_040530604	protein NDNF isoform X3	1.18	5.07E-03	K25687
NP_990729	translationally-controlled tumor protein homolog	1.19	1.27E-02	N/A
NP_990615	L-lactate dehydrogenase A chain isoform X1	1.19	3.62E-02	N/A
NP_001006169	nuclear distribution protein nudE homolog 1 isoform X1	1.19	3.58E-02	K16738
XP_040527467	serine/arginine repetitive matrix protein 3-like isoform X1	1.20	4.16E-02	N/A
XP_040512768	basic proline-rich protein-like isoform X2	1.20	1.17E-03	N/A
XP_040559305	myosin light chain 1, skeletal muscle isoform isoform X1	1.21	1.52E-04	K05738
NP_990615	L-lactate dehydrogenase A chain isoform X1	1.21	1.30E-02	K00016
NP_001263286	keratocan isoform X1	1.21	1.90E-03	K08122
NP_001264713	15-hydroxyprostaglandin dehydrogenase [NAD(+)] isoform X2	1.21	4.61E-03	K00069
NP_001232911	G-protein coupled receptor 39 isoform X1	1.22	7.34E-03	K04211
NP_990615	L-lactate dehydrogenase A chain isoform X1	1.25	1.92E-02	N/A
XP_027661403	uncharacterized protein LOC114015891	1.27	2.74E-02	N/A
NP_001274134	polyubiquitin-B isoform X1	1.27	7.49E-03	N/A
XP_010720134	uncharacterized protein LOC104909266 isoform X2	1.30	3.30E-02	N/A
NP_990615	L-lactate dehydrogenase A chain isoform X1	1.30	1.34E-02	N/A
XP_046768539	uncharacterized protein LOC124417788	1.31	1.26E-04	N/A
XP_015135433	extracellular serine/threonine protein kinase FAM20C	1.31	4.74E-03	K21957
NP_990615	L-lactate dehydrogenase A chain isoform X1	1.31	3.00E-02	N/A
XP_003642058	netrin-4 isoform X3	1.32	1.23E-02	K06845
NP_001038097	myosin light chain 1, skeletal muscle isoform isoform X1	1.32	2.09E-06	K05738
XP_040559305	myosin light chain 1, skeletal muscle isoform isoform X1	1.33	4.61E-02	N/A
NP_001274134	polyubiquitin-B isoform X1	1.34	3.11E-03	N/A
XP_046788681	protein S100-A4	1.34	9.46E-03	N/A
NP_989621	retinoid-binding protein 7	1.36	3.87E-02	K08753
XP_010720134	uncharacterized protein LOC104909266 isoform X2	1.36	2.53E-02	N/A
XP_004941346	serine/threonine-protein kinase PAK 6 isoform X1	1.37	5.31E-04	K05735
XP_040559305	myosin light chain 1, skeletal muscle isoform isoform X1	1.38	1.35E-02	N/A
YP_009555267	cytochrome c oxidase subunit III (mitochondrion)	1.38	3.85E-02	N/A
XP_040509146	gametocyte-specific factor 1 isoform X3	1.39	1.14E-02	N/A
NP_001038097	myosin light chain 1, skeletal muscle isoform isoform X1	1.39	6.26E-03	N/A
YP_009555270	NADH dehydrogenase subunit 4 (mitochondrion)	1.40	4.84E-02	N/A
NP_990605	myosin-11 isoform X1	1.41	1.55E-02	K10352
NP_001274134	polyubiquitin-B isoform X1	1.41	7.19E-04	N/A

Table S2 Cont.

NCBI Accession number	Gene description	log2FoldChange	P-value	KEGG Orthology ID
NP_990729	translationally-controlled tumor protein homolog	1.41	2.47E-02	N/A
XP_010720134	uncharacterized protein LOC104909266 isoform X2	1.42	2.10E-02	N/A
NP_990729	translationally-controlled tumor protein homolog	1.42	3.42E-02	N/A
YP_009555270	NADH dehydrogenase subunit 4 (mitochondrion)	1.42	4.82E-02	N/A
NP_001264467	protein stum homolog isoform X1	1.43	3.45E-02	N/A
YP_009555268	NADH dehydrogenase subunit 3 (mitochondrion)	1.43	4.82E-02	N/A
YP_009555272	cytochrome b (mitochondrion)	1.43	3.13E-02	N/A
XP_040503254	myosin-11 isoform X1	1.44	1.57E-02	K10352
YP_009555264	cytochrome c oxidase subunit II (mitochondrion)	1.44	3.70E-02	N/A
XP_010720134	uncharacterized protein LOC104909266 isoform X2	1.45	1.72E-02	K14211
XP_040504783	leukocyte receptor cluster member 9-like	1.46	2.70E-03	N/A
XP_040524270	interferon-induced very large GTPase 1	1.47	2.23E-02	N/A
XP_004934925	leiomodin-1	1.47	6.28E-03	K22030
XP_040512833	atherin-like isoform X1	1.47	1.05E-02	N/A
XP_010720134	uncharacterized protein LOC104909266 isoform X2	1.48	1.67E-02	N/A
XP_010720134	uncharacterized protein LOC104909266 isoform X2	1.49	1.28E-02	N/A
NP_001305331	complement component C6 isoform X1	1.50	3.16E-02	K03996
XP_010720134	uncharacterized protein LOC104909266 isoform X2	1.50	2.15E-02	N/A
YP_009555270	NADH dehydrogenase subunit 4 (mitochondrion)	1.51	4.05E-02	N/A
NP_001038141	polyamine-modulated factor 1	1.54	1.30E-02	N/A
NP_001025528	transgelin isoform X1	1.55	1.11E-02	K08673
NP_001161233	vitelline membrane outer layer protein 1 precursor	1.56	2.12E-02	N/A
XP_040531169	AMSH-like protease	1.58	1.37E-02	K11867
XP_010720134	uncharacterized protein LOC104909266 isoform X2	1.59	6.35E-03	K14211
YP_009555267	cytochrome c oxidase subunit III (mitochondrion)	1.61	2.59E-02	K02262
NP_990729	translationally-controlled tumor protein homolog	1.63	1.10E-02	N/A
YP_009555263	cytochrome c oxidase subunit I (mitochondrion)	1.64	3.35E-02	N/A
XP_040551279	uncharacterized protein LOC121113040	1.64	1.41E-02	N/A
YP_009555261	NADH dehydrogenase subunit 1 (mitochondrion)	1.64	2.89E-02	N/A
YP_009555267	cytochrome c oxidase subunit III (mitochondrion)	1.65	1.93E-02	K02262
XP_046800897	uncharacterized protein LOC124416949	1.66	2.85E-02	N/A
YP_009555264	cytochrome c oxidase subunit II (mitochondrion)	1.67	2.68E-02	N/A
NP_990729	translationally-controlled tumor protein homolog	1.74	4.31E-03	N/A
XP_428540	40S ribosomal protein S20	1.74	3.69E-03	K02969
YP_009555267	cytochrome c oxidase subunit III (mitochondrion)	1.74	2.90E-02	K02262
YP_009555266	ATP synthase F0 subunit 6 (mitochondrion)	1.74	2.24E-02	N/A
XP_025008312	nebulin isoform X33	1.75	2.74E-03	N/A
YP_009555264	cytochrome c oxidase subunit II (mitochondrion)	1.76	1.38E-02	N/A
YP_009555267	cytochrome c oxidase subunit III (mitochondrion)	1.78	1.68E-02	K02262
XP_030330632	elastin isoform X4	1.78	5.92E-03	N/A
NP_990753	extracellular fatty acid-binding protein precursor	1.81	4.06E-02	N/A
YP_009555269	NADH dehydrogenase subunit 4L (mitochondrion)	1.83	1.97E-02	N/A
YP_009555263	cytochrome c oxidase subunit I (mitochondrion)	1.86	2.62E-02	N/A
XP_042736445	cardiac phospholamban isoform X1	1.87	1.01E-03	N/A
NP_990729	translationally-controlled tumor protein homolog	1.87	1.80E-03	N/A
YP_009555267	cytochrome c oxidase subunit III (mitochondrion)	1.89	1.22E-02	N/A
XP_021249708	ras association domain-containing protein 6 isoform X1	1.90	6.17E-03	N/A
YP_009555264	cytochrome c oxidase subunit II (mitochondrion)	1.90	1.05E-02	N/A
YP_009555263	cytochrome c oxidase subunit I (mitochondrion)	1.96	1.65E-02	N/A
NP_990503	actin, aortic smooth muscle isoform X1	1.99	1.12E-02	K12315
YP_009555263	cytochrome c oxidase subunit I (mitochondrion)	2.03	1.39E-02	N/A
NP_990729	translationally-controlled tumor protein homolog	2.09	5.43E-04	N/A
YP_009555267	cytochrome c oxidase subunit III (mitochondrion)	2.09	1.48E-02	N/A
XP_040551279	serine/threonine-protein kinase TAO2 isoform X5	2.10	1.01E-02	N/A
YP_009555264	cytochrome c oxidase subunit II (mitochondrion)	2.11	4.76E-03	N/A
NP_990729	translationally-controlled tumor protein homolog	2.16	3.30E-03	N/A
XP_040559305	myosin light chain 1, skeletal muscle isoform isoform X1	2.20	7.42E-03	N/A
XP_419377	secretogranin-1 isoform X2	2.20	1.72E-04	K19991
YP_009555266	ATP synthase F0 subunit 6 (mitochondrion)	2.21	1.08E-02	N/A
NP_990838	creatine kinase B-type isoform X1	2.24	1.68E-03	K00933
XP_046760291	myosin light chain 1, skeletal muscle isoform isoform X1	2.27	1.87E-02	N/A
YP_009555263	cytochrome c oxidase subunit I (mitochondrion)	2.30	9.99E-03	N/A
NP_990316	phosphoglycerate kinase isoform X1	2.37	1.06E-02	K00927
NP_001188315	translation initiation factor IF-2-like isoform X2	2.37	4.27E-04	N/A
YP_009555266	ATP synthase F0 subunit 6 (mitochondrion)	2.41	7.65E-03	N/A
YP_009555263	cytochrome c oxidase subunit I (mitochondrion)	2.41	4.72E-03	N/A
YP_009555263	cytochrome c oxidase subunit I (mitochondrion)	2.44	3.45E-03	N/A

Table S2 Cont.

NCBI Accession number	Gene description	log2FoldChange	P-value	KEGG Orthology ID
XP_040504491	major histocompatibility complex class II beta chain BLB1, (HLA class II, D beta chain) isoform X1	2.46	5.33E-04	N/A
XP_046760291	myosin light chain 1, cardiac muscle isoform X1	2.48	2.78E-02	N/A
XP_046783746	integrin alpha-D isoform X13	2.49	2.43E-04	N/A
NP_001026400	actin, aortic smooth muscle isoform X1	2.51	3.11E-02	N/A
YP_009555263	cytochrome c oxidase subunit I (mitochondrion)	2.61	5.63E-03	N/A
NP_001269206	60S ribosomal protein L17 isoform X2	2.67	6.03E-03	K02880
YP_009555264	cytochrome c oxidase subunit II (mitochondrion)	2.69	1.82E-03	K02261
NP_001384222	major histocompatibility complex class I antigen BF2 isoform X1	2.80	9.47E-04	N/A
NP_990729	translationally-controlled tumor protein homolog	2.82	9.33E-04	N/A
YP_009555268	NADH dehydrogenase subunit 3 (mitochondrion)	2.83	8.74E-03	N/A
YP_009555262	NADH dehydrogenase subunit 2 (mitochondrion)	2.83	3.31E-03	N/A
NP_989866	osteopontin precursor	2.86	1.68E-02	K06250
YP_009555266	ATP synthase F0 subunit 6 (mitochondrion)	2.88	5.68E-03	K02126
NP_990847	calponin-2 isoform X1	2.90	9.97E-06	N/A
YP_009555263	cytochrome c oxidase subunit I (mitochondrion)	2.95	6.11E-03	N/A
YP_009555268	NADH dehydrogenase subunit 3 (mitochondrion)	3.10	4.46E-03	N/A
YP_009555268	NADH dehydrogenase subunit 3 (mitochondrion)	3.22	4.13E-03	N/A
NP_990316	phosphoglycerate kinase isoform X1	3.34	1.86E-03	K00927
YP_009555270	NADH dehydrogenase subunit 4 (mitochondrion)	3.49	2.68E-03	N/A
XP_015144626	myosin light chain 1, skeletal muscle isoform isoform X1	3.84	4.94E-05	N/A
NP_990729	translationally-controlled tumor protein homolog	3.94	3.17E-06	N/A
NP_001026400	actin, aortic smooth muscle isoform X1	4.22	2.67E-03	N/A
NP_990729	translationally-controlled tumor protein homolog	4.42	3.10E-06	N/A
XP_040559305	myosin light chain 1, skeletal muscle isoform isoform X1	4.42	3.56E-06	K05738
XP_025004078	RNA-binding protein 33 isoform X6	4.47	1.90E-03	N/A
XP_015144626	myosin light chain 1, skeletal muscle isoform isoform X1	4.71	3.91E-07	K05738
XP_015144626	myosin light chain 1, skeletal muscle isoform isoform X1	5.93	3.67E-04	N/A
XP_040559305	myosin light chain 1, skeletal muscle isoform isoform X1	6.50	3.44E-09	K05738
XP_040559305	myosin light chain 1, skeletal muscle isoform isoform X1	7.00	5.18E-04	N/A
XP_040559305	myosin light chain 1, skeletal muscle isoform isoform X1	8.99	1.47E-02	K05738
XP_015144626	myosin light chain 1, skeletal muscle isoform isoform X1	9.25	8.63E-06	N/A

N/A = not applicable

Color code presented in colume E

- DETs found only in NT
- DETs found in all breeds
- DETs found in BR and NT
- DETs found in NT and H75
