# Supplementary material for: Thermal impacts on transcriptome of Pectoralis major muscle collected from commercial broilers, Thai native chickens and its crossbreeds
Source: Anim Biosci. 2023 Oct 31;37(1):61–73. doi: 10.5713/ab.23.0195 (PMC10766454; doi:10.5713/ab.23.0195)
Supplement: Supplementary file 4 [file ab-23-0195-Supplementary-Table-3.pdf]

**Table S3** Differentially expressed transcripts (DETs) associated with thermal stress in P.major muscle of crossbred H75
Only annotated transcripts (from 1,107 DETs) are presented.

| NCBI Accession number | Gene description | log2FoldChange | P-value | KEGG Orthology ID |
|---|---|---|---|---|
| XP_046797375 | troponin T, fast skeletal muscle isoforms isoform X3 | -6.34 | 5.4E-08 | N/A |
| NP_001026400 | actin, aortic smooth muscle isoform X1 | -5.89 | 2.7E-05 | N/A |
| XP_046797375 | troponin T, fast skeletal muscle isoforms isoform X34 | -5.86 | 3.2E-06 | N/A |
| XP_025004078 | RNA-binding protein 33 isoform X6 | -4.60 | 5.6E-04 | N/A |
| NP_001026400 | actin, aortic smooth muscle isoform X1 | -4.57 | 8.9E-05 | N/A |
| XP_046797375 | troponin T, fast skeletal muscle isoforms isoform X3 | -3.90 | 2.0E-08 | K12046 |
| XP_015144626 | myosin light chain 1, skeletal muscle isoform isoform X1 | -3.78 | 5.4E-03 | N/A |
| XP_015144626 | myosin light chain 1, skeletal muscle isoform isoform X1 | -3.75 | 2.1E-02 | N/A |
| XP_015144626 | myosin light chain 1, skeletal muscle isoform isoform X1 | -3.71 | 3.3E-03 | N/A |
| NP_001072949 | actin, aortic smooth muscle isoform X1 | -3.68 | 1.1E-03 | K12314 |
| NP_001072949 | actin, aortic smooth muscle isoform X1 | -3.61 | 9.1E-05 | K12314 |
| XP_015142062 | troponin T, fast skeletal muscle isoforms isoform X34 | -3.37 | 1.0E-05 | K12046 |
| XP_046797375 | troponin T, fast skeletal muscle isoforms isoform X34 | -3.24 | 1.8E-08 | K12046 |
| NP_989554 | ATP-dependent 6-phosphofructokinase, platelet type isoform X4 | -3.09 | 2.8E-04 | N/A |
| XP_046794327 | methionine aminopeptidase 2 isoform X2 | -3.04 | 2.1E-05 | K01265 |
| XP_040551528 | uncharacterized protein LOC112531790 | -2.96 | 1.7E-06 | N/A |
| XP_015144626 | myosin light chain 1, skeletal muscle isoform isoform X1 | -2.92 | 1.3E-03 | N/A |
| NP_001269206 | 60S ribosomal protein L17 isoform X2 | -2.82 | 3.7E-03 | K02880 |
| XP_042669724 | troponin T, fast skeletal muscle isoform X5 | -2.77 | 6.2E-06 | N/A |
| XP_046797375 | troponin T, fast skeletal muscle isoforms isoform X34 | -2.73 | 2.9E-06 | K12046 |
| NP_990450 | alpha-enolase isoform X4 | -2.71 | 1.0E-02 | N/A |
| XP_015144626 | myosin light chain 1, skeletal muscle isoform isoform X1 | -2.63 | 1.8E-03 | N/A |
| NP_989931 | suppressor of cytokine signaling 1 isoform X1 | -2.59 | 8.5E-04 | N/A |
| XP_040550190 | EH domain-binding protein 1-like protein 1 isoform X9 | -2.38 | 1.6E-04 | N/A |
| XP_030904866 | troponin T, fast skeletal muscle | -2.34 | 2.5E-02 | N/A |
| XP_040550191 | EH domain-binding protein 1-like protein 1 isoform X10 | -2.33 | 2.9E-04 | N/A |
| XP_040560931 | E-selectin isoform X1 | -2.25 | 1.9E-03 | K06494 |
| XP_015144626 | myosin light chain 1, skeletal muscle isoform isoform X1 | -2.22 | 4.4E-03 | N/A |
| NP_001026205 | glycogen phosphorylase, liver form isoform X1 | -2.18 | 8.3E-03 | N/A |
| XP_015139877 | centriole, cilia and spindle-associated protein | -2.15 | 4.3E-04 | K16454 |
| NP_001161216 | creatine kinase B-type isoform X1 | -2.07 | 1.9E-02 | K00933 |
| XP_025004078 | RNA-binding protein 33 isoform X6 | -2.06 | 1.5E-03 | N/A |
| NP_001076829 | tumor necrosis factor receptor superfamily member 19 isoform X1 | -2.01 | 4.7E-04 | K05163 |
| NP_001384736 | NAD-dependent protein deacetylase sirtuin-1 isoform X3 | -1.91 | 1.4E-02 | K11412 |
| NP_990450 | alpha-enolase isoform X3 | -1.85 | 9.9E-03 | K01689 |
| NP_990838 | creatine kinase B-type isoform X1 | -1.80 | 1.2E-02 | K00933 |
| NP_001384361 | glycine amidinotransferase, mitochondrial | -1.80 | 2.6E-02 | K00613 |
| NP_001072949 | actin, aortic smooth muscle isoform X1 | -1.78 | 1.0E-03 | K12314 |
| XP_040550187 | EH domain-binding protein 1-like protein 1 isoform X7 | -1.75 | 8.2E-06 | N/A |
| XP_040503887 | RIMS-binding protein 2 isoform X10 | -1.74 | 1.6E-03 | K17591 |
| XP_025002525 | RNA-binding protein 33 isoform X6 | -1.72 | 1.2E-02 | N/A |
| XP_040551528 | protein THEMIS isoform X1 | -1.72 | 1.5E-02 | N/A |
| XP_040554202 | nidogen-1 isoform X2 | -1.71 | 1.8E-03 | K06826 |
| NP_990417 | ferritin heavy chain | -1.70 | 3.2E-02 | N/A |
| NP_001264667 | nephronectin isoform X8 | -1.70 | 2.4E-03 | K25527 |
| NP_001039301 | jun dimerization protein 2 isoform X1 | -1.69 | 3.5E-03 | K09030 |
| XP_046757901 | translation initiation factor IF-2-like isoform X2 | -1.68 | 2.6E-03 | N/A |
| XP_027641740 | uncharacterized protein LOC114011996 isoform X3 | -1.67 | 6.8E-06 | N/A |
| XP_046785444 | myosin, heavy chain 1G, skeletal muscle ( human myosin, heavy chain 1, skeletal muscle, adult) isoform X1 | -1.67 | 2.8E-02 | N/A |
| XP_046760898 | ATP-dependent 6-phosphofructokinase, platelet type isoform X3 | -1.66 | 7.3E-03 | N/A |
| XP_046797375 | troponin T, fast skeletal muscle isoforms isoform X31 | -1.66 | 3.1E-05 | N/A |
| XP_004938569 | four and a half LIM domains protein 2 isoform X1 | -1.66 | 2.4E-04 | K14380 |
| NP_001185673 | myosin regulatory light chain 2, smooth muscle minor isoform | -1.63 | 5.9E-03 | N/A |
| XP_040538312 | SRSF protein kinase 3 isoform X1 | -1.62 | 1.6E-03 | N/A |
| XP_046797375 | troponin T, fast skeletal muscle isoforms isoform X31 | -1.61 | 1.8E-04 | N/A |
| XP_046766168 | xin actin-binding repeat-containing protein 1 isoform X1 | -1.61 | 9.8E-03 | N/A |
| NP_001264535 | GTP-binding protein Rit2 isoform X1 | -1.61 | 2.7E-02 | K07845 |
| XP_046754177 | centrosome-associated protein 350 isoform X1 | -1.60 | 3.9E-04 | K16768 |
| NP_990729 | translationally-controlled tumor protein homolog | -1.59 | 3.2E-02 | N/A |
| XP_042669724 | troponin T, fast skeletal muscle isoform X5 | -1.59 | 1.2E-06 | N/A |
| XP_046785444 | myosin, heavy chain 1G, skeletal muscle ( human myosin, heavy chain 1, skeletal muscle, adult) isoform X1 | -1.59 | 1.7E-02 | N/A |
| NP_990406 | polyubiquitin-B isoform X1 | -1.57 | 1.1E-02 | N/A |
| XP_042669724 | troponin T, fast skeletal muscle isoform X5 | -1.55 | 3.6E-04 | N/A |
| NP_001292368 | sodium-coupled neutral amino acid transporter 4 isoform X1 | -1.54 | 1.3E-03 | K14207 |
| NP_990729 | translationally-controlled tumor protein homolog | -1.52 | 4.3E-02 | N/A |

**Table S3** Cont.

| NCBI Accession number | Gene description | log2FoldChange | P-value | KEGG Orthology ID |
|---|---|---|---|---|
| XP_046785444 | myosin, heavy chain 1G, skeletal muscle ( human myosin, heavy chain 1, skeletal muscle, adult) isoform X1 | -1.51 | 1.1E-03 | N/A |
| XP_042669724 | troponin T, fast skeletal muscle isoform X5 | -1.49 | 1.6E-03 | N/A |
| XP_046800897 | uncharacterized protein LOC124416949 | -1.49 | 4.8E-02 | N/A |
| XP_046754177 | centrosome-associated protein 350 isoform X1 | -1.48 | 2.0E-03 | K16768 |
| XP_040553268 | serine/threonine-protein kinase 38-like isoform X2 | -1.47 | 3.3E-03 | K08790 |
| XP_040538621 | WD repeat-containing protein 17 isoform X1 | -1.45 | 8.3E-04 | K13133 |
| XP_015134272 | tropomyosin alpha-1 chain isoform X11 | -1.45 | 1.2E-03 | N/A |
| XP_015139189 | tetratricopeptide repeat protein 7A isoform X1 | -1.45 | 1.3E-02 | K21843 |
| NP_001384338 | myosin, heavy chain 1G, skeletal muscle ( human myosin, heavy chain 1, skeletal muscle, adult) isoform X1 | -1.45 | 2.4E-03 | N/A |
| XP_415832 | protein phosphatase Slingshot homolog 2 isoform X1 | -1.44 | 4.5E-03 | K05766 |
| XP_046787128 | agrin isoform X3 | -1.44 | 6.6E-04 | K06254 |
| XP_025010479 | SRSF protein kinase 3 isoform X1 | -1.44 | 4.8E-03 | K08832 |
| NP_989908 | aldehyde dehydrogenase 1A1 isoform X1 | -1.43 | 3.0E-02 | K07249 |
| NP_990615 | L-lactate dehydrogenase A chain isoform X1 | -1.43 | 3.2E-02 | N/A |
| NP_001264599 | ankyrin repeat and SOCS box protein 1 | -1.43 | 2.3E-03 | N/A |
| NP_990615 | L-lactate dehydrogenase A chain isoform X1 | -1.43 | 4.7E-02 | K00016 |
| XP_424759 | phosphatidylinositol 3-kinase regulatory subunit alpha isoform X1 | -1.43 | 8.0E-03 | K02649 |
| NP_001383353 | insulin-induced gene 1 protein | -1.42 | 1.1E-02 | N/A |
| XP_040502661 | SRSF protein kinase 3 isoform X1 | -1.42 | 1.5E-03 | K08832 |
| XP_046790317 | nucleolin 1-like isoform X1 | -1.41 | 1.9E-02 | N/A |
| XP_042669724 | troponin T, fast skeletal muscle isoform X5 | -1.41 | 1.1E-04 | N/A |
| NP_990615 | L-lactate dehydrogenase A chain isoform X1 | -1.41 | 4.4E-02 | N/A |
| NP_001186431 | DEP domain-containing mTOR-interacting protein isoform X1 | -1.41 | 3.0E-03 | K20402 |
| XP_046797388 | troponin T, fast skeletal muscle isoforms isoform X31 | -1.41 | 1.6E-03 | N/A |
| XP_040520055 | uncharacterized protein LOC121109764 isoform X1 | -1.40 | 1.2E-02 | N/A |
| XP_003641574 | nebulette isoform X1 | -1.40 | 1.2E-02 | N/A |
| NP_001185673 | myosin regulatory light chain 2B, cardiac muscle isoform isoform X1 | -1.39 | 2.4E-02 | N/A |
| XP_015145980 | centrosome-associated protein 350 isoform X1 | -1.39 | 3.5E-03 | K16768 |
| NP_989554 | ATP-dependent 6-phosphofructokinase, platelet type isoform X3 | -1.39 | 6.1E-03 | K00850 |
| XP_042669724 | troponin T, fast skeletal muscle isoform X5 | -1.39 | 2.3E-05 | N/A |
| XP_046760763 | zinc finger protein 628 isoform X1 | -1.39 | 1.4E-03 | N/A |
| XP_046760898 | ATP-dependent 6-phosphofructokinase, platelet type isoform X3 | -1.38 | 4.3E-03 | K00850 |
| XP_015148144 | junctophilin-3 isoform X1 | -1.37 | 5.8E-04 | K19530 |
| XP_040547097 | transcription factor 24 isoform X2 | -1.37 | 3.3E-02 | N/A |
| XP_046797388 | troponin T, fast skeletal muscle isoforms isoform X31 | -1.37 | 1.3E-03 | N/A |
| XP_046783863 | guanine nucleotide-binding protein G(q) subunit alpha isoform X1 | -1.37 | 1.5E-03 | K04346 |
| XP_025010809 | platelet-derived growth factor receptor beta | -1.37 | 9.9E-03 | K05089 |
| NP_001186432 | phosphatidylserine synthase 2 isoform X4 | -1.36 | 3.1E-03 | K08730 |
| XP_027641738 | uncharacterized protein LOC114011996 isoform X2 | -1.36 | 4.5E-06 | N/A |
| XP_040550189 | EH domain-binding protein 1-like protein 1 isoform X8 | -1.35 | 9.0E-05 | N/A |
| NP_001384338 | myosin, heavy chain 1E, skeletal muscle isoform X1 | -1.35 | 5.2E-03 | N/A |
| XP_039585488 | uncharacterized protein LOC120511911 | -1.35 | 1.6E-03 | N/A |
| XP_040519922 | la-related protein 4B isoform X3 | -1.35 | 4.7E-03 | K18763 |
| XP_042669724 | troponin T, fast skeletal muscle isoform X5 | -1.35 | 5.5E-06 | N/A |
| NP_001384338 | myosin, heavy chain 1E, skeletal muscle isoform X1 | -1.35 | 2.9E-03 | N/A |
| NP_001026205 | glycogen phosphorylase, liver form isoform X1 | -1.34 | 2.7E-02 | N/A |
| NP_990699 | translation factor GUF1, mitochondrial isoform X1 | -1.34 | 2.7E-03 | N/A |
| XP_416534 | tyrosine-protein kinase STYK1 | -1.34 | 4.2E-04 | K17510 |
| XP_046797375 | troponin T, fast skeletal muscle isoforms isoform X31 | -1.34 | 4.9E-06 | N/A |
| XP_013029212 | unconventional myosin-XVIIIb | -1.33 | 5.1E-03 | N/A |
| XP_040525615 | AF4/FMR2 family member 1 isoform X1 | -1.32 | 3.7E-03 | K15184 |
| XP_046785444 | myosin, heavy chain 1G, skeletal muscle ( human myosin, heavy chain 1, skeletal muscle, adult) isoform X1 | -1.32 | 2.0E-02 | N/A |
| XP_046757901 | junctional adhesion molecule B isoform X1 | -1.32 | 5.0E-02 | N/A |
| XP_046785444 | myosin, heavy chain 1G, skeletal muscle ( human myosin, heavy chain 1, skeletal muscle, adult) isoform X1 | -1.32 | 3.6E-03 | N/A |
| NP_001026205 | glycogen phosphorylase, liver form isoform X1 | -1.31 | 1.5E-03 | N/A |
| NP_001006128 | glucose-6-phosphate isomerase | -1.31 | 9.2E-03 | N/A |
| XP_029890816 | alpha-actinin-2 isoform X2 | -1.31 | 1.0E-02 | N/A |
| XP_031469779 | troponin T, fast skeletal muscle | -1.30 | 1.9E-03 | N/A |
| XP_046785444 | myosin, heavy chain 1E, skeletal muscle isoform X1 | -1.30 | 5.7E-03 | N/A |
| NP_990615 | L-lactate dehydrogenase A chain isoform X1 | -1.30 | 2.9E-02 | K00016 |
| NP_001384338 | myosin, heavy chain 1E, skeletal muscle isoform X1 | -1.30 | 1.5E-03 | N/A |
| NP_001292368 | sodium-coupled neutral amino acid transporter 4 isoform X1 | -1.29 | 1.0E-02 | K14207 |
| XP_040517778 | centrosomal protein of 295 kDa isoform X1 | -1.29 | 6.8E-03 | N/A |
| NP_990615 | L-lactate dehydrogenase A chain isoform X1 | -1.29 | 2.1E-04 | N/A |
| XP_042669724 | troponin T, fast skeletal muscle isoform X5 | -1.29 | 2.3E-04 | N/A |
| XP_040511082 | EH domain-binding protein 1-like protein 1 isoform X2 | -1.29 | 2.1E-03 | N/A |
| XP_046796846 | amyloid beta precursor protein binding family B member 2 isoform X6 | -1.28 | 5.2E-04 | K04530 |

**Table S3** Cont.

| NCBI Accession number | Gene description | log2FoldChange | P-value | KEGG Orthology ID |
|---|---|---|---|---|
| NP_001384338 | myosin, heavy chain 1E, skeletal muscle isoform X1 | -1.28 | 1.1E-02 | N/A |
| XP_015132607 | D-2-hydroxyglutarate dehydrogenase, mitochondrial | -1.28 | 3.8E-04 | K18204 |
| XP_040512833 | atherin-like isoform X1 | -1.28 | 2.5E-02 | N/A |
| NP_001185673 | myosin regulatory light chain 2B, cardiac muscle isoform isoform X1 | -1.28 | 3.5E-02 | K12758 |
| NP_001384338 | myosin, heavy chain 1E, skeletal muscle isoform X1 | -1.28 | 3.1E-03 | N/A |
| NP_001383497 | alpha-2,8-sialyltransferase 8F precursor | -1.28 | 1.2E-02 | K06615 |
| NP_001384338 | myosin, heavy chain 1G, skeletal muscle ( human myosin, heavy chain 1, skeletal muscle, adult) isoform X1 | -1.27 | 1.2E-02 | N/A |
| NP_001171603 | fascin | -1.27 | 6.7E-03 | K23551 |
| NP_001026400 | actin, aortic smooth muscle isoform X1 | -1.27 | 1.1E-02 | N/A |
| XP_004936170 | rho-related GTP-binding protein RhoH | -1.27 | 6.0E-03 | K07873 |
| XP_040551528 | translation initiation factor IF-2-like isoform X2 | -1.26 | 1.6E-02 | N/A |
| NP_001013414 | myosin, heavy chain 1G, skeletal muscle ( human myosin, heavy chain 1, skeletal muscle, adult) isoform X1 | -1.26 | 1.3E-02 | N/A |
| NP_989575 | cryptochrome-1 isoform X1 | -1.26 | 6.4E-03 | K02295 |
| NP_001292368 | sodium-coupled neutral amino acid transporter 4 isoform X1 | -1.26 | 1.4E-02 | K14207 |
| XP_015147876 | phosphorylase b kinase regulatory subunit beta isoform X4 | -1.26 | 7.0E-03 | N/A |
| NP_990629 | protein C-ets-1 isoform X1 | -1.26 | 4.4E-03 | K02678 |
| NP_989559 | myosin, heavy chain 1G, skeletal muscle ( human myosin, heavy chain 1, skeletal muscle, adult) isoform X1 | -1.26 | 1.7E-02 | N/A |
| XP_015134272 | tropomyosin alpha-1 chain isoform X11 | -1.25 | 4.4E-03 | N/A |
| XP_040512595 | zinc finger protein 345 isoform X3 | -1.25 | 9.8E-04 | N/A |
| XP_046785444 | myosin, heavy chain 1G, skeletal muscle ( human myosin, heavy chain 1, skeletal muscle, adult) isoform X1 | -1.25 | 3.9E-03 | N/A |
| NP_001026205 | glycogen phosphorylase, liver form isoform X1 | -1.25 | 1.6E-02 | N/A |
| XP_046797375 | troponin T, fast skeletal muscle isoforms isoform X31 | -1.24 | 2.6E-04 | N/A |
| NP_001384338 | myosin, heavy chain 1G, skeletal muscle ( human myosin, heavy chain 1, skeletal muscle, adult) isoform X1 | -1.24 | 3.6E-03 | N/A |
| NP_001384338 | myosin, heavy chain 1G, skeletal muscle ( human myosin, heavy chain 1, skeletal muscle, adult) isoform X1 | -1.24 | 3.3E-03 | N/A |
| XP_001234010 | zinc finger SWIM domain-containing protein 6 isoform X1 | -1.24 | 9.7E-03 | N/A |
| NP_001108489 | protein adenylyltransferase SelO isoform X1 | -1.24 | 1.2E-02 | K08997 |
| NP_990615 | L-lactate dehydrogenase A chain isoform X1 | -1.24 | 3.3E-02 | K00016 |
| NP_001007848 | protein yippee-like 2 isoform X1 | -1.24 | 2.8E-02 | N/A |
| NP_001384338 | myosin, heavy chain 1G, skeletal muscle ( human myosin, heavy chain 1, skeletal muscle, adult) isoform X1 | -1.23 | 1.9E-02 | N/A |
| XP_040510275 | protein kinase C theta type | -1.23 | 1.8E-03 | K18052 |
| XP_015157021 | ATP-dependent RNA helicase DDX3X isoform X1 | -1.23 | 4.1E-03 | K11594 |
| XP_042669724 | troponin T, fast skeletal muscle isoform X5 | -1.23 | 2.0E-06 | N/A |
| NP_001384338 | myosin, heavy chain 1E, skeletal muscle isoform X1 | -1.23 | 3.3E-03 | N/A |
| XP_015139189 | tetratricopeptide repeat protein 7A isoform X1 | -1.23 | 1.2E-02 | K21843 |
| XP_015152693 | uncharacterized protein KIAA2013 homolog | -1.23 | 1.8E-03 | N/A |
| XP_015145045 | podocalyxin isoform X3 | -1.23 | 1.3E-03 | K06817 |
| XP_046793625 | neuroblast differentiation-associated protein AHNAK-like | -1.23 | 1.0E-02 | N/A |
| XP_046797375 | troponin T, fast skeletal muscle isoforms isoform X31 | -1.23 | 1.2E-05 | N/A |
| XP_040512782 | fructose-bisphosphate aldolase A | -1.22 | 1.3E-02 | K01623 |
| XP_042669724 | troponin T, fast skeletal muscle isoform X5 | -1.22 | 6.8E-05 | N/A |
| NP_001026160 | SH3 domain-binding protein 5 | -1.22 | 3.0E-03 | K23739 |
| XP_040502661 | SRSF protein kinase 3 isoform X1 | -1.22 | 6.2E-03 | K08832 |
| XP_025010809 | platelet-derived growth factor receptor beta | -1.22 | 2.2E-02 | K05089 |
| NP_001384338 | myosin, heavy chain 1E, skeletal muscle isoform X1 | -1.21 | 2.4E-03 | N/A |
| XP_015144626 | myosin light chain 1, skeletal muscle isoform isoform X1 | -1.21 | 1.3E-02 | N/A |
| XP_015137468 | dual specificity tyrosine-phosphorylation-regulated kinase 2 isoform X2 | -1.21 | 4.2E-03 | K18669 |
| XP_015141358 | bisphosphoglycerate mutase isoform X1 | -1.21 | 5.4E-03 | K01837 |
| XP_001235680 | adenylosuccinate synthetase isozyme 1 isoform X1 | -1.21 | 3.5E-03 | K01939 |
| XP_425039 | fructose-1,6-bisphosphatase isozyme 2 | -1.21 | 4.9E-03 | N/A |
| XP_414470 | ubiquitin-conjugating enzyme E2 D2 isoform X1 | -1.21 | 1.1E-03 | N/A |
| XP_040532043 | coiled-coil domain-containing protein 141 isoform X1 | -1.21 | 6.5E-03 | N/A |
| XP_040556677 | UV radiation resistance-associated gene protein isoform X9 | -1.21 | 2.6E-03 | K21249 |
| XP_046785444 | myosin, heavy chain 1E, skeletal muscle isoform X1 | -1.21 | 5.3E-03 | N/A |
| XP_046797375 | troponin T, fast skeletal muscle isoforms isoform X31 | -1.21 | 5.8E-06 | N/A |
| NP_001185673 | myosin regulatory light chain 2B, cardiac muscle isoform isoform X1 | -1.20 | 4.1E-02 | K12758 |
| XP_046794490 | alpha-actinin-2 isoform X1 | -1.20 | 1.3E-02 | N/A |
| XP_046755093 | semaphorin-6D isoform X2 | -1.20 | 2.1E-03 | K06842 |
| XP_046785444 | myosin, heavy chain 1E, skeletal muscle isoform X1 | -1.20 | 1.8E-03 | N/A |
| NP_001384338 | myosin, heavy chain 1G, skeletal muscle ( human myosin, heavy chain 1, skeletal muscle, adult) isoform X1 | -1.20 | 4.8E-03 | N/A |
| XP_015134281 | pyruvate kinase PKM isoform X1 | -1.20 | 2.5E-03 | K00873 |
| XP_015144517 | transcription factor COE3 isoform X19 | -1.20 | 3.5E-03 | K09103 |
| XP_046797317 | glycogen phosphorylase, liver form isoform X1 | -1.20 | 7.9E-03 | N/A |
| XP_025011798 | adenylate kinase isoenzyme 1 isoform X2 | -1.20 | 2.5E-03 | N/A |

**Table S3** Cont.

| NCBI Accession number | Gene description | log2FoldChange | P-value | KEGG Orthology ID |
|---|---|---|---|---|
| XP_046757901 | uncharacterized protein LOC124417285 | -1.20 | 1.4E-02 | N/A |
| XP_015154545 | dual specificity tyrosine-phosphorylation-regulated kinase 3 isoform X3 | -1.20 | 4.8E-03 | K18669 |
| XP_040511818 | uncharacterized protein LOC101752072 | -1.19 | 4.7E-02 | N/A |
| NP_001384338 | myosin, heavy chain 1E, skeletal muscle isoform X1 | -1.19 | 5.7E-03 | N/A |
| XP_040535878 | semaphorin-6D isoform X1 | -1.19 | 3.8E-03 | K06842 |
| XP_001233649 | essential MCU regulator, mitochondrial | -1.19 | 1.2E-02 | N/A |
| XP_046785444 | myosin, heavy chain 1E, skeletal muscle isoform X1 | -1.19 | 4.8E-03 | N/A |
| XP_015144626 | myosin light chain 1, skeletal muscle isoform isoform X1 | -1.19 | 1.0E-02 | K05738 |
| NP_001384338 | myosin, heavy chain 1E, skeletal muscle isoform X1 | -1.18 | 3.0E-03 | N/A |
| XP_040560734 | P-selectin isoform X1 | -1.18 | 2.8E-02 | K06496 |
| NP_990615 | L-lactate dehydrogenase A chain isoform X1 | -1.18 | 4.1E-02 | K00016 |
| NP_001384338 | myosin, heavy chain 1E, skeletal muscle isoform X1 | -1.18 | 8.2E-03 | N/A |
| XP_046770701 | pleckstrin homology domain-containing family G member 1 isoform X1 | -1.18 | 2.9E-02 | K23859 |
| XP_046785444 | myosin, heavy chain 1E, skeletal muscle isoform X1 | -1.17 | 4.8E-03 | N/A |
| NP_001026290 | rab GDP dissociation inhibitor beta isoform X1 | -1.17 | 4.0E-03 | K23460 |
| XP_046797119 | dynein axonemal assembly factor 9 isoform X4 | -1.17 | 1.3E-02 | K25424 |
| XP_040503750 | uncharacterized protein LOC121106827 isoform X1 | -1.17 | 6.8E-03 | N/A |
| XP_025009576 | pyruvate kinase PKM isoform X1 | -1.17 | 8.0E-04 | N/A |
| XP_015141358 | bisphosphoglycerate mutase isoform X1 | -1.16 | 6.3E-03 | K01837 |
| XP_040551273 | dual specificity mitogen-activated protein kinase kinase 4 isoform X7 | -1.16 | 1.7E-02 | K04431 |
| XP_040538392 | TATA element modulatory factor isoform X2 | -1.16 | 2.4E-02 | K20286 |
| XP_040538309 | SRSF protein kinase 3 isoform X1 | -1.16 | 4.6E-03 | K08832 |
| XP_040547258 | neuron navigator 1 isoform X1 | -1.16 | 4.4E-03 | K16776 |
| NP_001383860 | ectonucleoside triphosphate diphosphohydrolase 4 isoform X4 | -1.16 | 7.3E-03 | K01511 |
| XP_046797388 | troponin T, fast skeletal muscle isoforms isoform X31 | -1.16 | 5.1E-04 | N/A |
| XP_040549828 | nuclear receptor subfamily 4 group A member 1 | -1.16 | 2.2E-03 | N/A |
| NP_001306233 | myosin, heavy chain 1E, skeletal muscle isoform X1 | -1.16 | 2.0E-02 | N/A |
| NP_001264581 | exportin-5 isoform X3 | -1.16 | 9.0E-03 | K14289 |
| XP_425039 | fructose-1,6-bisphosphatase isozyme 2 | -1.16 | 6.6E-03 | N/A |
| XP_046756343 | glucocorticoid receptor isoform X1 | -1.16 | 4.5E-03 | K05771 |
| XP_421308 | tyrosine-protein phosphatase non-receptor type 21 isoform X1 | -1.16 | 1.3E-02 | K18025 |
| NP_001006128 | glucose-6-phosphate isomerase | -1.15 | 7.0E-03 | N/A |
| XP_004936089 | leucine-rich repeat-containing protein 66 isoform X1 | -1.15 | 4.0E-02 | N/A |
| XP_046771286 | copper-transporting ATPase 1 isoform X1 | -1.15 | 1.2E-02 | K17686 |
| NP_001025749 | kelch domain-containing protein 4 isoform X1 | -1.15 | 6.7E-04 | N/A |
| NP_990450 | alpha-enolase isoform X4 | -1.15 | 5.8E-03 | N/A |
| NP_001007973 | terminal nucleotidyltransferase 5A isoform X1 | -1.15 | 2.9E-02 | K23034 |
| NP_001185673 | myosin regulatory light chain 2B, cardiac muscle isoform isoform X1 | -1.15 | 4.8E-02 | K12758 |
| XP_040522255 | nesprin-1 isoform X6 | -1.15 | 4.9E-03 | K19326 |
| NP_001012712 | transmembrane protein 131-like isoform X6 | -1.15 | 3.1E-02 | N/A |
| NP_001384338 | myosin, heavy chain 1E, skeletal muscle isoform X1 | -1.15 | 3.8E-03 | N/A |
| NP_990482 | glial fibrillary acidic protein | -1.15 | 4.6E-02 | K10378 |
| NP_001384338 | myosin, heavy chain 1G, skeletal muscle ( human myosin, heavy chain 1, skeletal muscle, adult) isoform X1 | -1.14 | 1.3E-02 | N/A |
| XP_426030 | leucine-rich repeat-containing protein 14B | -1.14 | 8.6E-04 | N/A |
| NP_001103255 | heat shock cognate protein HSP 90-beta isoform X1 | -1.14 | 2.2E-02 | K04079 |
| XP_040529665 | E3 ubiquitin-protein ligase UBR1 isoform X1 | -1.14 | 7.8E-03 | K10625 |
| XP_015134282 | pyruvate kinase PKM isoform X1 | -1.14 | 3.7E-03 | N/A |
| XP_015151487 | SH2B adapter protein 2 | -1.14 | 2.0E-02 | K07193 |
| XP_024998174 | junctophilin-2 isoform X1 | -1.14 | 1.9E-02 | K19530 |
| XP_040512595 | zinc finger protein 316-like | -1.14 | 7.9E-03 | N/A |
| XP_001235321 | T-box transcription factor TBX3 isoform X1 | -1.14 | 1.3E-02 | K10176 |
| XP_040510275 | protein kinase C theta type | -1.14 | 5.6E-03 | K18052 |
| XP_040512782 | fructose-bisphosphate aldolase A | -1.13 | 5.9E-03 | N/A |
| NP_001384338 | myosin, heavy chain 1E, skeletal muscle isoform X1 | -1.13 | 7.2E-03 | N/A |
| XP_004945480 | SEC14-like protein 5 | -1.13 | 2.6E-02 | N/A |
| XP_046785444 | myosin, heavy chain 1G, skeletal muscle ( human myosin, heavy chain 1, skeletal muscle, adult) isoform X1 | -1.13 | 9.4E-03 | N/A |
| XP_046785003 | protein phosphatase 1 regulatory subunit 26 | -1.13 | 6.2E-03 | K17565 |
| XP_015154831 | microtubule-associated protein tau isoform X13 | -1.13 | 1.9E-03 | K04380 |
| XP_046771160 | cAMP-specific 3',5'-cyclic phosphodiesterase 7B isoform X3 | -1.13 | 5.8E-03 | K18436 |
| NP_001034694 | 5'-AMP-activated protein kinase catalytic subunit alpha-2 isoform X1 | -1.13 | 1.3E-02 | K07198 |
| NP_990615 | L-lactate dehydrogenase A chain isoform X1 | -1.13 | 4.1E-02 | N/A |
| XP_040509123 | microtubule-associated protein tau isoform X13 | -1.13 | 3.3E-03 | K04380 |
| XP_001233314 | regulator of G-protein signaling 4 isoform X3 | -1.13 | 1.0E-02 | N/A |
| XP_046785444 | myosin, heavy chain 1G, skeletal muscle ( human myosin, heavy chain 1, skeletal muscle, adult) isoform X1 | -1.12 | 1.7E-02 | N/A |
| NP_001384338 | myosin, heavy chain 1G, skeletal muscle ( human myosin, heavy chain 1, skeletal muscle, adult) isoform X1 | -1.12 | 1.5E-03 | N/A |
| XP_040514061 | tetratricopeptide repeat protein 38 | -1.12 | 1.6E-02 | N/A |
| XP_040537337 | F-box only protein 31 isoform X1 | -1.12 | 1.5E-02 | K10308 |

**Table S3** Cont.

| NCBI Accession number | Gene description | log2FoldChange | P-value | KEGG Orthology ID |
|---|---|---|---|---|
| XP_040526464 | vascular endothelial growth factor receptor kdr-like isoform X3 | -1.12 | 7.4E-03 | K05096 |
| NP_990699 | translation factor GUF1, mitochondrial isoform X2 | -1.12 | 6.1E-04 | K03234 |
| XP_046785444 | myosin, heavy chain 1G, skeletal muscle ( human myosin, heavy chain 1, skeletal muscle, adult) isoform X1 | -1.12 | 1.4E-02 | N/A |
| XP_015141960 | signal peptide, CUB and EGF-like domain-containing protein 2 isoform X1 | -1.12 | 3.0E-02 | K24706 |
| NP_001376362 | PDZ and LIM domain protein 5 isoform X8 | -1.11 | 9.6E-03 | N/A |
| XP_040512782 | fructose-bisphosphate aldolase A | -1.11 | 1.7E-02 | K01623 |
| XP_015149958 | transcription factor E4F1 isoform X1 | -1.11 | 9.4E-03 | K22401 |
| NP_001385203 | actin, aortic smooth muscle isoform X1 | -1.11 | 6.6E-03 | K10354 |
| XP_417013 | Krueppel-like factor 5 | -1.11 | 3.7E-02 | K09206 |
| XP_040552862 | tau-tubulin kinase 1 isoform X4 | -1.11 | 2.4E-03 | K08815 |
| NP_989792 | rho-related GTP-binding protein RhoQ isoform X1 | -1.11 | 1.9E-03 | N/A |
| XP_025004076 | RNA-binding protein 33 isoform X2 | -1.11 | 4.9E-02 | N/A |
| NP_001384338 | myosin, heavy chain 1G, skeletal muscle ( human myosin, heavy chain 1, skeletal muscle, adult) isoform X1 | -1.11 | 1.3E-02 | N/A |
| XP_046785444 | myosin, heavy chain 1G, skeletal muscle ( human myosin, heavy chain 1, skeletal muscle, adult) isoform X1 | -1.11 | 9.0E-03 | N/A |
| NP_001383860 | ectonucleoside triphosphate diphosphohydrolase 8 isoform X1 | -1.11 | 1.0E-02 | K01511 |
| XP_004935978 | chloride intracellular channel protein 5 isoform X2 | -1.11 | 7.0E-03 | K05025 |
| XP_003642396 | dual specificity mitogen-activated protein kinase kinase 6 isoform X1 | -1.11 | 1.0E-02 | K04433 |
| XP_015143216 | tyrosine-protein phosphatase non-receptor type 21 isoform X1 | -1.10 | 1.6E-02 | K18025 |
| XP_040542390 | UPF0450 protein C17orf58 homolog isoform X2 | -1.10 | 3.6E-03 | N/A |
| XP_040502338 | kelch repeat and BTB domain-containing protein 12 isoform X1 | -1.10 | 1.9E-02 | K21912 |
| XP_040558238 | ankyrin-3 isoform X12 | -1.10 | 2.4E-02 | K10380 |
| XP_015151566 | serine/threonine-protein kinase TAO1 isoform X1 | -1.10 | 1.9E-02 | K04429 |
| XP_015140933 | NEDD4-binding protein 2 isoform X2 | -1.10 | 1.7E-02 | K15720 |
| NP_001376260 | protein O-GlcNAcase isoform X9 | -1.10 | 1.5E-02 | K15719 |
| NP_001013414 | myosin, heavy chain 1G, skeletal muscle ( human myosin, heavy chain 1, skeletal muscle, adult) isoform X1 | -1.10 | 2.4E-02 | N/A |
| XP_040560337 | nebulin isoform X22 | -1.10 | 2.6E-02 | K18267 |
| NP_990450 | alpha-enolase isoform X3 | -1.10 | 3.2E-02 | K01689 |
| NP_001033782 | phosphoglucomutase-1 isoform X1 | -1.10 | 2.7E-03 | K01835 |
| XP_040524619 | dystonin isoform X32 | -1.10 | 1.3E-02 | K10382 |
| NP_001384338 | myosin, heavy chain 1G, skeletal muscle ( human myosin, heavy chain 1, skeletal muscle, adult) isoform X1 | -1.10 | 4.7E-03 | K24220 |
| NP_001012553 | eukaryotic translation initiation factor 3 subunit L | -1.09 | 3.8E-03 | N/A |
| XP_046785444 | myosin, heavy chain 1G, skeletal muscle ( human myosin, heavy chain 1, skeletal muscle, adult) isoform X1 | -1.09 | 9.2E-03 | N/A |
| XP_046799790 | protein TANC1 isoform X4 | -1.09 | 1.4E-02 | N/A |
| XP_015134833 | syntaxin-binding protein 1 isoform X2 | -1.09 | 9.4E-04 | K15292 |
| NP_990615 | L-lactate dehydrogenase A chain isoform X1 | -1.09 | 4.2E-02 | N/A |
| XP_040533019 | nebulin isoform X4 | -1.09 | 4.5E-02 | K18267 |
| XP_414455 | plexin-B2 isoform X1 | -1.09 | 5.7E-03 | K06822 |
| XP_046777675 | titin isoform X2 | -1.09 | 1.9E-02 | K12567 |
| NP_001033782 | phosphoglucomutase-1 isoform X1 | -1.09 | 6.7E-04 | N/A |
| XP_040512782 | fructose-bisphosphate aldolase A | -1.09 | 8.2E-03 | K01623 |
| XP_040541036 | oxysterol-binding protein 2 isoform X1 | -1.09 | 1.5E-02 | K20462 |
| XP_001232578 | glucose-fructose oxidoreductase domain-containing protein 1 isoform X2 | -1.09 | 1.5E-02 | N/A |
| XP_040544294 | uncharacterized protein C20orf85 homolog | -1.09 | 2.7E-04 | N/A |
| NP_990850 | sarcoplasmic/endoplasmic reticulum calcium ATPase 2 isoform X1 | -1.09 | 4.6E-02 | N/A |
| NP_001305361 | carnosine N-methyltransferase 2 isoform X1 | -1.08 | 1.1E-02 | K22437 |
| XP_015154822 | microtubule-associated protein tau isoform X1 | -1.08 | 1.4E-03 | K04380 |
| XP_416045 | anoctamin-6 isoform X1 | -1.08 | 1.4E-02 | K19500 |
| NP_001026205 | glycogen phosphorylase, liver form isoform X1 | -1.08 | 2.0E-02 | N/A |
| NP_001027570 | elongation factor 1-alpha 2 isoform X1 | -1.08 | 2.5E-02 | K03231 |
| XP_422671 | serine/threonine-protein kinase PAK 2 | -1.08 | 6.7E-03 | K04410 |
| NP_990615 | L-lactate dehydrogenase A chain isoform X1 | -1.08 | 1.5E-02 | K00016 |
| NP_989792 | cell division control protein 42 homolog isoform X2 | -1.08 | 1.9E-03 | N/A |
| XP_025964749 | ankyrin repeat domain-containing protein 10 isoform X1 | -1.08 | 1.7E-02 | N/A |
| XP_015142274 | serine/threonine-protein phosphatase 6 regulatory subunit 3 isoform X1 | -1.08 | 1.1E-02 | K15501 |
| XP_015142631 | retroelement silencing factor 1 isoform X2 | -1.08 | 1.9E-02 | N/A |
| NP_001292573 | ubiquitin-protein ligase E3B isoform X1 | -1.08 | 1.6E-02 | K10588 |
| NP_001273190 | LIM domain-binding protein 3 isoform X1 | -1.07 | 2.4E-02 | N/A |
| XP_004942418 | protein artemis isoform X1 | -1.07 | 9.2E-03 | N/A |
| NP_001384338 | myosin, heavy chain 1E, skeletal muscle isoform X1 | -1.07 | 6.9E-03 | K24220 |
| NP_001384338 | myosin, heavy chain 1E, skeletal muscle isoform X1 | -1.07 | 9.8E-03 | N/A |
| XP_046778710 | GPI-anchor transamidase isoform X3 | -1.07 | 3.2E-03 | K05290 |
| XP_015146511 | phosphoglucomutase-1 isoform X1 | -1.07 | 9.0E-03 | N/A |
| NP_001013414 | myosin, heavy chain 1G, skeletal muscle ( human myosin, heavy chain 1, skeletal muscle, adult) isoform X1 | -1.07 | 9.2E-03 | N/A |

**Table S3** Cont.

| NCBI Accession number | Gene description | log2FoldChange | P-value | KEGG Orthology ID |
|---|---|---|---|---|
| NP_001385203 | actin, alpha skeletal muscle B | -1.07 | 1.9E-03 | N/A |
| XP_046785444 | myosin, heavy chain 1E, skeletal muscle isoform X1 | -1.07 | 2.1E-03 | N/A |
| NP_001384338 | myosin, heavy chain 1G, skeletal muscle ( human myosin, heavy chain 1, skeletal muscle, adult) isoform X1 | -1.07 | 5.8E-03 | K24220 |
| XP_414586 | coiled-coil domain-containing protein 69 isoform X2 | -1.07 | 1.2E-02 | N/A |
| XP_004935669 | pleckstrin homology domain-containing family G member 1 isoform X1 | -1.07 | 3.1E-02 | K23859 |
| XP_040508947 | homeodomain-interacting protein kinase 1 isoform X1 | -1.07 | 1.1E-02 | K08826 |
| XP_040542493 | BAH and coiled-coil domain-containing protein 1 | -1.07 | 6.9E-03 | N/A |
| XP_040504575 | MHC BF1 class I isoform X1 | -1.06 | 2.3E-02 | N/A |
| NP_001376561 | glycerol-3-phosphate dehydrogenase 1-like protein isoform X1 | -1.06 | 8.7E-03 | N/A |
| XP_419118 | inositol monophosphatase 1 isoform X1 | -1.06 | 2.2E-03 | K01092 |
| XP_025003384 | collagen alpha-1(XV) chain isoform X5 | -1.06 | 3.3E-03 | K08135 |
| NP_990615 | L-lactate dehydrogenase A chain isoform X1 | -1.06 | 1.6E-02 | K00016 |
| NP_001376358 | PDZ and LIM domain protein 5 isoform X4 | -1.06 | 1.5E-02 | N/A |
| NP_001026251 | arginine/serine-rich protein PNISR isoform X1 | -1.06 | 9.3E-04 | K13170 |
| XP_040559750 | coiled-coil domain-containing protein 141 isoform X1 | -1.06 | 1.4E-02 | N/A |
| XP_046777663 | nebulin isoform X39 | -1.06 | 2.7E-02 | K18267 |
| NP_001006128 | glucose-6-phosphate isomerase | -1.06 | 5.7E-03 | N/A |
| XP_040526352 | uncharacterized protein KIAA0232 homolog isoform X4 | -1.06 | 7.3E-03 | N/A |
| XP_040518978 | histone-lysine N-methyltransferase 2C isoform X11 | -1.06 | 1.3E-02 | K09188 |
| XP_040512954 | neuroblast differentiation-associated protein AHNAK isoform X1 | -1.06 | 4.3E-02 | K23934 |
| NP_001384338 | myosin, heavy chain 1E, skeletal muscle isoform X1 | -1.06 | 1.1E-02 | N/A |
| NP_990482 | glial fibrillary acidic protein | -1.06 | 4.4E-02 | K10378 |
| NP_001306233 | myosin, heavy chain 1E, skeletal muscle isoform X1 | -1.06 | 3.2E-02 | N/A |
| XP_040527126 | uncharacterized protein C4orf54 homolog | -1.06 | 1.0E-02 | N/A |
| NP_001384173 | N-alpha-acetyltransferase 60 | -1.06 | 9.4E-03 | K20793 |
| NP_001384338 | myosin, heavy chain 1G, skeletal muscle ( human myosin, heavy chain 1, skeletal muscle, adult) isoform X1 | -1.06 | 6.0E-03 | N/A |
| XP_015137447 | ankyrin repeat and IBR domain-containing protein 1 isoform X1 | -1.06 | 9.8E-03 | K11967 |
| XP_046794490 | alpha-actinin-2 isoform X1 | -1.06 | 2.4E-02 | N/A |
| XP_046794490 | alpha-actinin-2 isoform X1 | -1.05 | 3.0E-02 | K21073 |
| NP_990450 | alpha-enolase isoform X3 | -1.05 | 4.3E-02 | N/A |
| NP_001384338 | myosin, heavy chain 1E, skeletal muscle isoform X1 | -1.05 | 1.2E-02 | N/A |
| NP_001006347 | OTU domain-containing protein 3 | -1.05 | 7.7E-03 | K18342 |
| XP_046785444 | myosin, heavy chain 1G, skeletal muscle ( human myosin, heavy chain 1, skeletal muscle, adult) isoform X1 | -1.05 | 1.6E-02 | N/A |
| NP_001384338 | myosin, heavy chain 1G, skeletal muscle ( human myosin, heavy chain 1, skeletal muscle, adult) isoform X1 | -1.05 | 1.1E-02 | N/A |
| XP_046785444 | myosin, heavy chain 1G, skeletal muscle ( human myosin, heavy chain 1, skeletal muscle, adult) isoform X1 | -1.05 | 9.2E-03 | N/A |
| YP_009555268 | NADH dehydrogenase subunit 3 (mitochondrion) | 1.05 | 3.6E-02 | N/A |
| XP_040562735 | death-associated protein kinase 2 isoform X5 | 1.05 | 2.1E-03 | K08803 |
| NP_001384159 | neurogenic differentiation factor 2 | 1.06 | 1.7E-02 | K09084 |
| NP_001185673 | myosin regulatory light chain 2B, cardiac muscle isoform isoform X1 | 1.06 | 2.9E-03 | N/A |
| XP_040524192 | CAD protein isoform X1 | 1.06 | 2.1E-03 | K11540 |
| NP_990850 | sarcoplasmic/endoplasmic reticulum calcium ATPase 2 isoform X1 | 1.06 | 4.0E-02 | N/A |
| NP_989483 | 60S ribosomal protein L6 | 1.06 | 2.6E-02 | K02934 |
| XP_040507059 | period circadian protein homolog 3 isoform X8 | 1.06 | 6.5E-03 | N/A |
| XP_046793905 | cleavage and polyadenylation specificity factor subunit 1 isoform X1 | 1.07 | 3.2E-03 | N/A |
| XP_040521260 | X-linked retinitis pigmentosa GTPase regulator isoform X2 | 1.07 | 2.0E-02 | N/A |
| NP_001161236 | heat shock factor protein 2 isoform X2 | 1.07 | 6.3E-03 | K09415 |
| XP_023797542 | LWamide neuropeptides isoform X2 | 1.07 | 9.3E-03 | N/A |
| XP_418406 | pyrroline-5-carboxylate reductase 3 isoform X1 | 1.07 | 1.4E-03 | K00286 |
| XP_003641100 | protein Mpv17 isoform X10 | 1.07 | 7.0E-03 | N/A |
| XP_046800897 | uncharacterized protein LOC124416949 | 1.07 | 2.2E-04 | N/A |
| XP_025011115 | dnaJ homolog subfamily A member 3, mitochondrial isoform X1 | 1.08 | 8.0E-03 | N/A |
| XP_040515784 | C-type lectin domain family 2 member D isoform X2 | 1.08 | 1.4E-02 | N/A |
| XP_040527827 | rho-related BTB domain-containing protein 2 isoform X1 | 1.08 | 3.4E-03 | N/A |
| XP_046762151 | IgGFc-binding protein | 1.08 | 5.9E-03 | N/A |
| XP_015142944 | serine/threonine-protein kinase VRK1 isoform X3 | 1.09 | 5.2E-03 | N/A |
| XP_040541845 | cytoplasmic dynein 1 intermediate chain 2 isoform X3 | 1.09 | 8.2E-04 | K22868 |
| XP_046797401 | troponin T, fast skeletal muscle isoforms isoform X32 | 1.09 | 1.1E-02 | K12046 |
| NP_990838 | creatine kinase B-type isoform X1 | 1.09 | 3.1E-02 | N/A |
| XP_046757725 | surfeit locus protein 6 isoform X1 | 1.09 | 1.6E-02 | N/A |
| NP_990847 | calponin-2 isoform X1 | 1.09 | 2.7E-03 | K04405 |
| XP_025010451 | coiled-coil domain-containing glutamate-rich protein 1-like | 1.09 | 2.9E-03 | N/A |
| XP_024999849 | pyruvate dehydrogenase kinase, isozyme 1 isoform X1 | 1.09 | 4.0E-03 | N/A |
| YP_009555262 | NADH dehydrogenase subunit 2 (mitochondrion) | 1.09 | 1.6E-02 | N/A |
| XP_015141316 | adenosine deaminase 2 isoform X2 | 1.10 | 7.3E-03 | K19572 |
| XP_015145042 | zinc finger protein 385B isoform X1 | 1.10 | 3.6E-02 | N/A |
| NP_990265 | gelsolin isoform X5 | 1.10 | 2.2E-03 | N/A |

**Table S3** Cont.

| NCBI Accession number | Gene description | log2FoldChange | P-value | KEGG Orthology ID |
|---|---|---|---|---|
| XP_040559305 | myosin light chain 1, skeletal muscle isoform isoform X1 | 1.10 | 8.0E-03 | N/A |
| YP_009555264 | cytochrome c oxidase subunit II (mitochondrion) | 1.11 | 1.7E-02 | N/A |
| NP_001070702 | TSC22 domain family protein 3 isoform X1 | 1.11 | 3.2E-03 | N/A |
| XP_038031345 | transmembrane protein 107-like | 1.11 | 4.6E-03 | K22764 |
| NP_001384338 | myosin, heavy chain 1G, skeletal muscle ( human myosin, heavy chain 1, skeletal muscle, adult) isoform X1 | 1.11 | 6.7E-03 | N/A |
| XP_025011798 | adenylate kinase isoenzyme 1 isoform X2 | 1.11 | 2.0E-02 | K00939 |
| XP_424187 | ELMO domain-containing protein 3 isoform X7 | 1.11 | 2.0E-02 | K09516 |
| NP_989479 | protein S100-A4 | 1.11 | 2.2E-02 | N/A |
| NP_001269268 | gem-associated protein 4 isoform X2 | 1.11 | 3.8E-04 | K13132 |
| XP_040550995 | IgGFc-binding protein | 1.11 | 6.6E-03 | N/A |
| XP_040511996 | aminopeptidase O isoform X1 | 1.12 | 3.2E-03 | N/A |
| YP_009555261 | NADH dehydrogenase subunit 1 (mitochondrion) | 1.12 | 6.4E-03 | N/A |
| XP_040553621 | pleckstrin homology domain-containing family H member 2 isoform X4 | 1.12 | 7.1E-03 | K24020 |
| NP_001244262 | 60S ribosomal protein L9 | 1.12 | 7.8E-03 | N/A |
| NP_001383608 | glial fibrillary acidic protein | 1.13 | 2.9E-02 | N/A |
| XP_015132014 | ankyrin repeat and SOCS box protein 5 isoform X1 | 1.13 | 7.7E-04 | K10327 |
| XP_021238657 | ribonuclease H2 subunit A | 1.13 | 4.2E-02 | N/A |
| NP_001038097 | myosin light chain 1, skeletal muscle isoform isoform X1 | 1.13 | 9.0E-03 | N/A |
| NP_001185673 | myosin regulatory light chain 2B, cardiac muscle isoform isoform X1 | 1.13 | 7.5E-04 | K12758 |
| NP_990590 | angiogenin precursor | 1.13 | 3.3E-02 | N/A |
| NP_001383608 | glial fibrillary acidic protein | 1.14 | 2.4E-02 | N/A |
| YP_009555272 | cytochrome b (mitochondrion) | 1.14 | 4.0E-04 | N/A |
| XP_040393256 | keratin-associated protein 16-1-like | 1.14 | 3.1E-02 | N/A |
| NP_990063 | melanoma inhibitory activity protein 2 isoform X1 | 1.14 | 4.8E-02 | N/A |
| XP_046764561 | protein artemis isoform X1 | 1.14 | 2.7E-06 | N/A |
| NP_001026060 | retinoid-binding protein 7 | 1.14 | 1.5E-02 | N/A |
| XP_015132541 | transcription cofactor HES-6 isoform X1 | 1.14 | 2.3E-03 | N/A |
| NP_001026060 | retinoid-binding protein 7 | 1.15 | 4.7E-02 | K08752 |
| NP_996726 | cbp/p300-interacting transactivator 2 isoform X1 | 1.15 | 2.4E-03 | N/A |
| YP_009555273 | NADH dehydrogenase subunit 6 (mitochondrion) | 1.15 | 4.4E-02 | K03884 |
| XP_040541619 | complement component C8 gamma chain | 1.15 | 9.6E-03 | N/A |
| XP_046777675 | titin isoform X2 | 1.15 | 2.7E-02 | N/A |
| NP_001185673 | myosin regulatory light chain 2B, cardiac muscle isoform isoform X1 | 1.16 | 8.4E-03 | N/A |
| XP_040509563 | uncharacterized protein LOC121107733 isoform X1 | 1.16 | 2.7E-02 | N/A |
| XP_046760291 | myosin light chain 1, cardiac muscle isoform X1 | 1.16 | 5.1E-03 | N/A |
| NP_990781 | calcium-binding protein 1 isoform X2 | 1.17 | 2.2E-02 | K12042 |
| XP_040550437 | aggrecan core protein isoform X1 | 1.17 | 3.3E-02 | N/A |
| NP_001384338 | myosin, heavy chain 1G, skeletal muscle ( human myosin, heavy chain 1, skeletal muscle, adult) isoform X1 | 1.17 | 1.4E-03 | N/A |
| NP_990690 | insulin-like growth factor-binding protein 2 isoform X1 | 1.17 | 1.4E-02 | K23575 |
| XP_025001024 | aminopeptidase O isoform X2 | 1.17 | 2.8E-03 | K09606 |
| YP_009555261 | NADH dehydrogenase subunit 1 (mitochondrion) | 1.17 | 3.9E-02 | N/A |
| NP_001038097 | myosin light chain 1, skeletal muscle isoform isoform X1 | 1.17 | 5.3E-03 | N/A |
| XP_015132976 | adenosine 5'-monophosphoramidase HINT2 isoform X1 | 1.17 | 5.9E-03 | N/A |
| XP_015132563 | pituitary tumor-transforming gene 1 protein-interacting protein | 1.17 | 5.7E-04 | N/A |
| XP_015145042 | zinc finger protein 385B isoform X1 | 1.17 | 2.7E-02 | N/A |
| XP_015149473 | dihydropyrimidinase-related protein 2 isoform X1 | 1.18 | 3.7E-02 | N/A |
| XP_040511996 | aminopeptidase O isoform X1 | 1.18 | 4.5E-03 | N/A |
| XP_036255864 | elastin-like | 1.19 | 4.5E-02 | N/A |
| XP_025011798 | adenylate kinase isoenzyme 1 isoform X2 | 1.19 | 9.6E-03 | K00939 |
| NP_001278663 | adaptor-related protein complex 3, sigma 2 subunit isoform X4 | 1.19 | 2.3E-04 | N/A |
| NP_990265 | gelsolin isoform X5 | 1.19 | 2.2E-03 | N/A |
| XP_004939021 | tripartite motif-containing protein 3 isoform X1 | 1.19 | 2.2E-03 | N/A |
| XP_001231707 | leucine rich adaptor protein 1-like | 1.20 | 1.6E-03 | N/A |
| XP_040559305 | myosin light chain 1, skeletal muscle isoform isoform X1 | 1.20 | 6.4E-03 | N/A |
| XP_424530 | dual specificity protein phosphatase 13 | 1.20 | 7.9E-03 | N/A |
| NP_990850 | sarcoplasmic/endoplasmic reticulum calcium ATPase 2 isoform X1 | 1.20 | 3.1E-03 | N/A |
| NP_001264847 | 5'-deoxynucleotidase HDDC2 | 1.21 | 5.6E-03 | K07023 |
| NP_001264404 | translocon-associated protein subunit gamma | 1.21 | 4.4E-03 | N/A |
| XP_032297331 | thioredoxin-like protein 1 isoform X2 | 1.21 | 2.9E-03 | N/A |
| XP_046760291 | myosin light chain 1, cardiac muscle isoform X1 | 1.21 | 1.0E-02 | N/A |
| XP_015145676 | coiled-coil domain-containing protein 148 isoform X1 | 1.22 | 4.7E-03 | N/A |
| XP_040543276 | serine/threonine-protein kinase ULK2 | 1.22 | 2.1E-03 | K08269 |
| NP_990781 | calcium-binding protein 7 | 1.22 | 3.9E-03 | N/A |
| XP_004942337 | nebulin-related-anchoring protein isoform X2 | 1.22 | 2.0E-03 | N/A |
| XP_040507693 | four and a half LIM domains protein 3 isoform X3 | 1.22 | 1.6E-02 | K24414 |
| XP_010559460 | CTD small phosphatase-like protein isoform X1 | 1.23 | 2.6E-03 | N/A |
| XP_040559305 | myosin light chain 1, skeletal muscle isoform isoform X1 | 1.23 | 8.4E-03 | N/A |
| XP_046762151 | alpha-tectorin isoform X1 | 1.23 | 3.6E-03 | N/A |
| XP_040521830 | lymphocyte antigen 96 isoform X1 | 1.23 | 2.7E-02 | N/A |

**Table S3** Cont.

| NCBI Accession number | Gene description | log2FoldChange | P-value | KEGG Orthology ID |
|---|---|---|---|---|
| XP_046760291 | myosin light chain 1, cardiac muscle isoform X1 | 1.23 | 8.1E-03 | N/A |
| NP_001006464 | transmembrane protein 258 | 1.23 | 3.0E-02 | N/A |
| XP_040529704 | acyl-coenzyme A thioesterase 1 isoform X1 | 1.24 | 9.8E-03 | K01068 |
| NP_990781 | calcium-binding protein 1 isoform X2 | 1.24 | 1.2E-02 | N/A |
| XP_031463340 | polypeptide N-acetylgalactosaminyltransferase-like 6 | 1.24 | 1.1E-02 | N/A |
| XP_010559460 | CTD small phosphatase-like protein isoform X1 | 1.24 | 4.3E-04 | N/A |
| XP_046760291 | myosin light chain 1, skeletal muscle isoform isoform X1 | 1.24 | 2.1E-02 | N/A |
| XP_015138133 | DNA repair-scaffolding protein isoform X1 | 1.25 | 1.8E-02 | K22806 |
| NP_990218 | protein Wnt-5a isoform 4 precursor | 1.25 | 1.2E-02 | K00444 |
| YP_009555264 | cytochrome c oxidase subunit II (mitochondrion) | 1.25 | 4.1E-03 | N/A |
| XP_046798503 | aldo-keto reductase family 1, member B1-like isoform X4 | 1.25 | 3.4E-02 | N/A |
| XP_004940389 | sestrin-1 isoform X9 | 1.26 | 6.7E-03 | K10141 |
| XP_040513007 | DNA-directed RNA polymerase II subunit RPB7 isoform X2 | 1.26 | 5.0E-02 | N/A |
| NP_001264438 | cytochrome c oxidase subunit VIa polypeptide 1 isoform 1 | 1.26 | 1.0E-02 | N/A |
| XP_024999633 | peptidase inhibitor 16 isoform X3 | 1.27 | 3.2E-02 | K20412 |
| NP_001264467 | protein stum homolog isoform X1 | 1.27 | 4.8E-02 | N/A |
| XP_046762151 | IgGFc-binding protein isoform X1 | 1.28 | 2.2E-03 | N/A |
| XP_015134967 | endophilin-B2 isoform X5 | 1.29 | 7.5E-03 | K21269 |
| XP_046760291 | myosin light chain 1, cardiac muscle isoform X1 | 1.29 | 6.9E-03 | N/A |
| XP_046795499 | secretogranin-1 isoform X1 | 1.29 | 1.1E-02 | N/A |
| XP_046760291 | myosin light chain 1, skeletal muscle isoform isoform X1 | 1.30 | 1.6E-02 | N/A |
| XP_046761930 | adenosine 5'-monophosphoramidase HINT2 isoform X2 | 1.31 | 8.3E-04 | N/A |
| XP_040525234 | alpha-protein kinase 1 isoform X1 | 1.31 | 6.0E-03 | K08868 |
| NP_001157123 | membrane progestin receptor alpha isoform X1 | 1.31 | 1.4E-03 | K25039 |
| XP_040521830 | lymphocyte antigen 96 isoform X1 | 1.31 | 3.8E-02 | N/A |
| NP_001385018 | fibroblast growth factor 18 isoform X1 | 1.31 | 1.1E-03 | K04358 |
| XP_004942202 | pantothenate kinase 1 isoform X3 | 1.32 | 4.7E-04 | K09680 |
| XP_015153240 | E3 ubiquitin-protein ligase TRIM63 isoform X1 | 1.32 | 3.4E-03 | N/A |
| YP_009555264 | cytochrome c oxidase subunit II (mitochondrion) | 1.33 | 3.9E-02 | N/A |
| XP_040559305 | myosin light chain 1, skeletal muscle isoform isoform X1 | 1.33 | 8.3E-03 | N/A |
| XP_015143726 | DNA damage-inducible transcript 4 protein | 1.33 | 1.9E-03 | N/A |
| XP_025002247 | homeobox protein ARX isoform X1 | 1.33 | 1.3E-02 | K09452 |
| NP_990856 | apolipoprotein A-I isoform X1 | 1.34 | 1.4E-03 | N/A |
| XP_040550924 | GRIP1-associated protein 1 | 1.35 | 4.2E-04 | N/A |
| NP_990838 | creatine kinase B-type isoform X1 | 1.35 | 2.1E-02 | N/A |
| NP_001004379 | NHP2-like protein 1 | 1.35 | 4.8E-02 | N/A |
| XP_040559305 | myosin light chain 1, skeletal muscle isoform isoform X1 | 1.35 | 1.6E-02 | N/A |
| XP_025004397 | nephrocystin-1 isoform X5 | 1.35 | 1.9E-03 | K19657 |
| XP_419796 | sestrin-1 isoform X2 | 1.35 | 2.0E-03 | K10141 |
| XP_040546718 | extracellular matrix protein 1 | 1.36 | 8.4E-03 | K23867 |
| XP_040510184 | leukocyte immunoglobulin-like receptor subfamily B member 1 isoform X2 | 1.36 | 3.9E-02 | N/A |
| XP_046797375 | troponin T, fast skeletal muscle isoforms isoform X31 | 1.36 | 4.3E-04 | K12046 |
| XP_025008312 | nebulin isoform X33 | 1.37 | 2.0E-02 | N/A |
| XP_046797375 | troponin T, fast skeletal muscle isoforms isoform X3 | 1.37 | 1.8E-02 | K12046 |
| NP_990782 | triosephosphate isomerase | 1.37 | 2.4E-02 | K01803 |
| NP_001038097 | myosin light chain 1, skeletal muscle isoform isoform X1 | 1.37 | 9.4E-03 | N/A |
| NP_990781 | caltractin isoform X4 | 1.37 | 7.5E-04 | K12042 |
| XP_003640379 | calcium/calmodulin-dependent protein kinase type 1D isoform X1 | 1.37 | 2.2E-02 | K08794 |
| XP_025006024 | uncharacterized protein LOC112532354 | 1.37 | 1.0E-02 | N/A |
| XP_046760291 | myosin light chain, embryonic isoform X2 | 1.38 | 4.1E-03 | N/A |
| NP_001026488 | neuronal acetylcholine receptor subunit alpha-6 isoform X1 | 1.38 | 1.1E-03 | K04816 |
| NP_001280103 | polyubiquitin-B isoform X1 | 1.39 | 8.9E-03 | N/A |
| XP_025002251 | homeobox protein ARX isoform X2 | 1.39 | 1.3E-02 | K09452 |
| XP_429201 | aminopeptidase Q isoform X2 | 1.39 | 2.8E-02 | K13724 |
| NP_001005431 | peptidyl-prolyl cis-trans isomerase FKBP5 isoform X1 | 1.39 | 4.4E-02 | K09571 |
| XP_015155653 | arrestin domain-containing protein 2 isoform X1 | 1.40 | 8.2E-04 | N/A |
| XP_040557674 | interleukin-1 receptor type 1 isoform X3 | 1.40 | 7.8E-03 | N/A |
| NP_001188315 | translation initiation factor IF-2-like isoform X2 | 1.40 | 3.8E-02 | N/A |
| NP_990785 | transforming growth factor beta-3 proprotein preproprotein | 1.41 | 6.2E-03 | N/A |
| NP_001384338 | myosin, heavy chain 1G, skeletal muscle ( human myosin, heavy chain 1, skeletal muscle, adult) isoform X1 | 1.41 | 1.1E-03 | N/A |
| XP_015138070 | myosin regulatory light chain 2, smooth muscle minor isoform | 1.41 | 5.0E-03 | K12757 |
| NP_001028817 | epoxide hydrolase 4 | 1.42 | 1.8E-03 | K08726 |
| XP_015133085 | periostin isoform X6 | 1.42 | 3.9E-02 | K24790 |
| NP_001305915 | fibronectin type III domain-containing protein 5 precursor | 1.42 | 6.3E-03 | K24486 |
| YP_009555270 | NADH dehydrogenase subunit 4 (mitochondrion) | 1.42 | 4.9E-02 | N/A |
| NP_001264653 | paired mesoderm homeobox protein 1 isoform X2 | 1.43 | 1.8E-02 | K09329 |
| XP_021249708 | ras association domain-containing protein 6 isoform X1 | 1.44 | 2.0E-02 | N/A |
| XP_046790065 | leukocyte immunoglobulin-like receptor subfamily B member 5 isoform X2 | 1.44 | 3.2E-02 | N/A |
| XP_001233329 | myosin regulatory light chain 2, smooth muscle minor isoform | 1.44 | 1.3E-04 | N/A |
| XP_046769491 | nephrocystin-1 isoform X5 | 1.45 | 3.5E-04 | K19657 |

**Table S3** Cont.

| NCBI Accession number | Gene description | log2FoldChange | P-value | KEGG Orthology ID |
|---|---|---|---|---|
| XP_015134677 | tubulin polymerization-promoting protein isoform X3 | 1.45 | 2.0E-02 | N/A |
| NP_990265 | gelsolin isoform X5 | 1.45 | 1.6E-03 | N/A |
| XP_040551478 | obscurin isoform X18 | 1.45 | 1.1E-03 | N/A |
| NP_001001472 | NF-kappa-B inhibitor epsilon | 1.45 | 9.1E-08 | K04734 |
| XP_030308940 | actin-binding LIM protein 1 isoform X7 | 1.46 | 5.8E-04 | N/A |
| NP_001038097 | myosin light chain 1, skeletal muscle isoform isoform X1 | 1.46 | 3.1E-03 | N/A |
| XP_040529159 | reticulon-4 receptor-like 2 isoform X1 | 1.46 | 2.0E-03 | K16661 |
| NP_001263291 | pleiotrophin isoform X1 | 1.46 | 4.6E-02 | K16642 |
| XP_015154188 | troponin T, cardiac muscle isoforms isoform X3 | 1.47 | 1.1E-02 | K12045 |
| NP_001038097 | myosin light chain 1, skeletal muscle isoform isoform X1 | 1.47 | 6.3E-03 | N/A |
| XP_046760291 | myosin light chain 1, cardiac muscle isoform X1 | 1.47 | 3.5E-02 | N/A |
| XP_003642935 | ectoderm-neural cortex protein 1 | 1.49 | 1.9E-03 | K10462 |
| NP_001007943 | ribonuclease homolog precursor | 1.49 | 1.3E-02 | K16631 |
| NP_998745 | serine/threonine-protein kinase VRK1 isoform X1 | 1.49 | 7.3E-03 | N/A |
| NP_001376430 | phosphatidylinositol-glycan biosynthesis class X protein precursor | 1.50 | 8.9E-03 | K07541 |
| NP_990521 | proto-oncogene tyrosine-protein kinase receptor Ret isoform X2 | 1.50 | 4.5E-03 | K05126 |
| NP_001384338 | myosin, heavy chain 1G, skeletal muscle ( human myosin, heavy chain 1, skeletal muscle, adult) isoform X1 | 1.51 | 2.0E-04 | N/A |
| XP_015154253 | negative elongation factor E | 1.52 | 4.7E-03 | N/A |
| XP_015153518 | neural cell adhesion molecule 1 isoform X1 | 1.52 | 1.2E-03 | K06491 |
| XP_030330632 | elastin isoform X4 | 1.53 | 1.8E-02 | N/A |
| NP_001155848 | LYR motif-containing protein 1 isoform X3 | 1.54 | 4.2E-04 | N/A |
| NP_001025748 | cytochrome c oxidase subunit 4 isoform 1, mitochondrial isoform X1 | 1.54 | 7.2E-03 | N/A |
| XP_040557300 | potassium voltage-gated channel subfamily C member 1 isoform X5 | 1.55 | 1.3E-02 | K04887 |
| XP_046797388 | troponin T, fast skeletal muscle isoforms isoform X31 | 1.55 | 3.1E-03 | K12046 |
| XP_040559305 | myosin light chain 1, skeletal muscle isoform isoform X1 | 1.56 | 2.4E-02 | N/A |
| NP_001026739 | neuronal acetylcholine receptor subunit alpha-4 precursor | 1.56 | 1.4E-03 | K04818 |
| NP_990850 | sarcoplasmic/endoplasmic reticulum calcium ATPase 2 isoform X1 | 1.56 | 3.1E-02 | N/A |
| XP_041338926 | cleavage and polyadenylation specificity factor subunit 1 | 1.56 | 4.0E-04 | N/A |
| NP_001005431 | peptidyl-prolyl cis-trans isomerase FKBP5 isoform X1 | 1.56 | 1.5E-02 | K09571 |
| NP_001383551 | collagen alpha-1(III) chain isoform X1 | 1.57 | 3.9E-02 | N/A |
| YP_009555270 | NADH dehydrogenase subunit 4 (mitochondrion) | 1.58 | 3.5E-02 | N/A |
| NP_989508 | L-lactate dehydrogenase B chain isoform X1 | 1.58 | 9.2E-04 | K00016 |
| XP_046788681 | protein S100-A4 | 1.58 | 2.2E-03 | N/A |
| NP_990850 | sarcoplasmic/endoplasmic reticulum calcium ATPase 2 isoform X1 | 1.59 | 2.8E-02 | N/A |
| XP_030904866 | troponin T, fast skeletal muscle | 1.60 | 5.8E-03 | N/A |
| XP_040504414 | acetyl-CoA carboxylase 2 isoform X1 | 1.60 | 2.0E-03 | K01946 |
| XP_015148981 | sodium-coupled neutral amino acid transporter 4 isoform X1 | 1.60 | 4.9E-03 | K13576 |
| XP_041875748 | tRNA (guanine(26)-N(2))-dimethyltransferase | 1.61 | 1.6E-03 | N/A |
| XP_003642935 | ectoderm-neural cortex protein 1 | 1.61 | 2.9E-04 | K10462 |
| XP_015154181 | nuclear receptor-interacting protein 1 | 1.62 | 2.5E-02 | N/A |
| XP_418775 | 60S ribosomal protein L14 | 1.63 | 6.4E-03 | K02875 |
| XP_040542866 | tumor protein D52 isoform X12 | 1.67 | 4.7E-03 | N/A |
| NP_989887 | homeodomain-only protein | 1.69 | 1.1E-03 | N/A |
| NP_001280103 | polyubiquitin-B isoform X1 | 1.71 | 1.7E-04 | N/A |
| NP_001384338 | myosin, heavy chain 1E, skeletal muscle isoform X1 | 1.74 | 9.4E-04 | N/A |
| XP_015133368 | insulin receptor substrate 2 isoform X3 | 1.75 | 2.8E-02 | K07187 |
| XP_040559305 | myosin light chain 1, skeletal muscle isoform isoform X1 | 1.77 | 2.8E-03 | N/A |
| NP_001107181 | myosin, heavy chain 1E, skeletal muscle isoform X1 | 1.77 | 1.7E-02 | N/A |
| XP_040524298 | scavenger receptor class A member 5 isoform X1 | 1.77 | 3.6E-04 | N/A |
| NP_989887 | homeodomain-only protein | 1.78 | 6.7E-04 | N/A |
| XP_046765712 | uncharacterized protein LOC124417654 | 1.81 | 2.4E-03 | N/A |
| NP_001289119 | small muscular protein isoform X1 | 1.82 | 1.5E-02 | N/A |
| XP_040513173 | N-alpha-acetyltransferase 20 isoform X1 | 1.82 | 2.4E-02 | K20791 |
| NP_001026060 | fatty acid-binding protein, heart | 1.83 | 1.9E-03 | N/A |
| XP_025010788 | myotilin isoform X2 | 1.84 | 1.2E-03 | K19875 |
| XP_046758011 | myosin, heavy chain 1G, skeletal muscle ( human myosin, heavy chain 1, skeletal muscle, adult) isoform X1 | 1.84 | 4.0E-04 | N/A |
| XP_040525299 | secretogranin-1 isoform X1 | 1.85 | 2.9E-05 | N/A |
| NP_001264945 | LYR motif-containing protein 2 | 1.86 | 1.0E-04 | N/A |
| XP_025010788 | myotilin isoform X2 | 1.86 | 1.0E-03 | K19875 |
| NP_990512 | stathmin-3 isoform X1 | 1.87 | 2.0E-03 | N/A |
| NP_001383551 | collagen alpha-1(III) chain isoform X1 | 1.89 | 1.0E-03 | N/A |
| NP_001038145 | granulysin precursor | 1.90 | 9.5E-04 | N/A |
| XP_015142062 | troponin T, fast skeletal muscle isoforms isoform X34 | 1.90 | 1.1E-03 | K12046 |
| XP_015143261 | nesprin-1 isoform X8 | 1.91 | 1.7E-02 | N/A |
| NP_001384338 | myosin, heavy chain 1G, skeletal muscle ( human myosin, heavy chain 1, skeletal muscle, adult) isoform X1 | 1.93 | 6.5E-04 | N/A |
| NP_001289119 | small muscular protein isoform X1 | 1.93 | 9.7E-03 | N/A |
| NP_001384338 | myosin, heavy chain 1G, skeletal muscle ( human myosin, heavy chain 1, skeletal muscle, adult) isoform X1 | 1.97 | 1.7E-03 | N/A |

**Table S3** Cont.

| NCBI Accession number | Gene description | log2FoldChange | P-value | KEGG Orthology ID |
|---|---|---|---|---|
| NP_990651 | avidin-related protein 4/5 | 1.99 | 3.5E-02 | N/A |
| XP_046797388 | troponin T, fast skeletal muscle isoforms isoform X31 | 2.01 | 3.1E-02 | N/A |
| NP_001264322 | aldo-keto reductase family 1 member D1 isoform X1 | 2.07 | 3.1E-05 | K00251 |
| NP_990598 | nucleophosmin | 2.09 | 2.0E-02 | N/A |
| NP_996868 | leukocyte cell-derived chemotaxin 1 | 2.12 | 5.5E-03 | N/A |
| NP_001032920 | tyrosine-protein phosphatase non-receptor type substrate 1 isoform X1 | 2.13 | 4.7E-02 | N/A |
| XP_031463340 | polypeptide N-acetylgalactosaminyltransferase-like 6 | 2.14 | 4.4E-03 | N/A |
| NP_990847 | calponin-2 isoform X1 | 2.17 | 8.0E-04 | N/A |
| NP_001186838 | pyruvate dehydrogenase kinase, isozyme 3 isoform X1 | 2.18 | 2.8E-05 | K00898 |
| NP_001384338 | myosin, heavy chain 1G, skeletal muscle ( human myosin, heavy chain 1, skeletal muscle, adult) isoform X1 | 2.19 | 2.4E-02 | N/A |
| XP_040559305 | myosin light chain 1, skeletal muscle isoform isoform X1 | 2.21 | 6.5E-03 | N/A |
| XP_015147511 | homer protein homolog 2 isoform X1 | 2.22 | 5.9E-04 | K15010 |
| XP_025011798 | adenylate kinase isoenzyme 1 isoform X2 | 2.22 | 4.3E-02 | K00939 |
| NP_990298 | keratocan isoform X1 | 2.22 | 1.9E-02 | K08121 |
| XP_417248 | prolyl 4-hydroxylase subunit alpha-1 isoform X1 | 2.23 | 1.4E-02 | N/A |
| NP_001384338 | myosin, heavy chain 1E, skeletal muscle isoform X1 | 2.31 | 5.1E-03 | N/A |
| NP_990598 | nucleophosmin | 2.35 | 1.2E-02 | N/A |
| NP_990729 | translationally-controlled tumor protein homolog | 2.37 | 5.1E-03 | N/A |
| XP_046784130 | Ig lambda chain V-1 region isoform X34 | 2.38 | 9.8E-03 | N/A |
| NP_990850 | sarcoplasmic/endoplasmic reticulum calcium ATPase 2 isoform X1 | 2.39 | 7.8E-04 | N/A |
| XP_040556620 | troponin T, fast skeletal muscle isoforms isoform X31 | 2.40 | 6.7E-04 | N/A |
| XP_425240 | complement C1q tumor necrosis factor-related protein 8 | 2.42 | 3.2E-02 | K19470 |
| XP_025011798 | adenylate kinase isoenzyme 1 isoform X2 | 2.42 | 3.5E-05 | N/A |
| XP_015142062 | troponin T, fast skeletal muscle isoforms isoform X34 | 2.44 | 2.3E-04 | N/A |
| XP_015142342 | smoothelin isoform X2 | 2.48 | 4.2E-05 | N/A |
| NP_990782 | triosephosphate isomerase | 2.49 | 4.9E-03 | N/A |
| XP_416113 | 40S ribosomal protein S16 | 2.52 | 6.9E-03 | N/A |
| NP_989559 | myosin, heavy chain 1E, skeletal muscle isoform X1 | 2.54 | 6.8E-04 | N/A |
| NP_001384338 | myosin, heavy chain 1G, skeletal muscle ( human myosin, heavy chain 1, skeletal muscle, adult) isoform X1 | 2.58 | 1.6E-03 | N/A |
| XP_040526936 | MAP7 domain-containing protein 3 isoform X8 | 2.59 | 2.9E-04 | K16807 |
| NP_990777 | tropomyosin beta chain isoform X10 | 2.76 | 8.3E-05 | N/A |
| XP_015140228 | collagen alpha-1(XII) chain isoform X1 | 2.79 | 1.2E-03 | K08132 |
| XP_040552767 | collagen alpha-1(XII) chain isoform X1 | 2.90 | 1.4E-03 | N/A |
| NP_001013414 | myosin, heavy chain 1G, skeletal muscle ( human myosin, heavy chain 1, skeletal muscle, adult) isoform X1 | 3.19 | 7.6E-04 | N/A |
| XP_046765984 | myosin-binding protein C, slow-type | 3.46 | 6.3E-03 | K12557 |
| XP_029897170 | myosin-binding protein C, slow-type isoform X4 | 3.51 | 1.8E-05 | N/A |
| XP_001234114 | four and a half LIM domains protein 1 isoform X2 | 3.62 | 6.3E-04 | N/A |
| NP_001013414 | myosin, heavy chain 1G, skeletal muscle ( human myosin, heavy chain 1, skeletal muscle, adult) isoform X1 | 3.87 | 4.5E-05 | N/A |
| XP_001234114 | four and a half LIM domains protein 1 isoform X2 | 3.90 | 5.1E-04 | N/A |
| XP_025011798 | adenylate kinase isoenzyme 1 isoform X2 | 3.92 | 6.1E-06 | K00939 |
| XP_015132747 | tropomyosin beta chain isoform X2 | 4.04 | 9.7E-06 | N/A |
| XP_427836 | alpha-crystallin B chain isoform X1 | 4.18 | 9.2E-06 | K09546 |
| XP_416940 | protein ADP-ribosylarginine hydrolase-like protein 1 isoform X1 | 4.18 | 2.7E-04 | N/A |
| NP_989866 | osteopontin precursor | 4.96 | 3.4E-05 | K06250 |
| XP_040531040 | 4-hydroxy-2-oxoglutarate aldolase, mitochondrial | 5.34 | 5.6E-06 | N/A |
| NP_001264340 | carbonic anhydrase 13 | 5.36 | 3.9E-03 | K01672 |
| NP_001264756 | myozenin-2 isoform X1 | 5.75 | 8.4E-06 | N/A |
| XP_025011798 | adenylate kinase isoenzyme 1 isoform X2 | 6.45 | 1.4E-06 | K00939 |
| XP_025011798 | adenylate kinase isoenzyme 1 isoform X2 | 7.92 | 2.8E-07 | K00939 |
| NP_001161224 | myoglobin isoform X1 | 7.98 | 3.4E-08 | K21892 |

N/A = not applicable

Color code presented in colume E

DETs found only in H75

DETs found in all breeds

DETs found in BR and H75

DETs found in NT and H75
