# Supplementary material for: Thermal impacts on transcriptome of Pectoralis major muscle collected from commercial broilers, Thai native chickens and its crossbreeds
Source: Anim Biosci. 2023 Oct 31;37(1):61–73. doi: 10.5713/ab.23.0195 (PMC10766454; doi:10.5713/ab.23.0195)
Supplement: Supplementary file 5 [file ab-23-0195-Supplementary-Table-4.pdf]

Table S4 KEGG pathways associated with thermal stress within commercial broilers

KEGG Pathway	Mapped protein
ko01100 Metabolic pathways (15)	ko:K00016 LDH; L-lactate dehydrogenase [EC:1.1.1.27]
	ko:K00157 AOX; aldehyde oxidase [EC:1.2.3.1]
	ko:K00507 SCD; stearoyl-CoA desaturase (Delta-9 desaturase) [EC:1.14.19.1]
	ko:K00799 GST; glutathione S-transferase [EC:2.5.1.18]
	ko:K00820 glmS; glutamine---fructose-6-phosphate transaminase (isomerizing) [EC:2.6.1.16]
	ko:K00933 E2.7.3.2; creatine kinase [EC:2.7.3.2]
	ko:K01513 ENPP1_3; ectonucleotide pyrophosphatase/phosphodiesterase family member 1/3 [EC:3.1.4.1 3.6.1.9]
	ko:K01623 ALDO; fructose-bisphosphate aldolase, class I [EC:4.1.2.13]
	ko:K01689 ENO; enolase [EC:4.2.1.11]
	ko:K01697 CBS; cystathionine beta-synthase [EC:4.2.1.22]
	ko:K01807 rpiA; ribose 5-phosphate isomerase A [EC:5.3.1.6]
	ko:K01834 PGAM; 2,3-bisphosphoglycerate-dependent phosphoglycerate mutase [EC:5.4.2.11]
	ko:K07249 ALDH1A; retinal dehydrogenase [EC:1.2.1.36]
	ko:K15789 TDH; threonine 3-dehydrogenase [EC:1.1.1.103]
	ko:K18245 CA2; carbonic anhydrase 2 [EC:4.2.1.1]
ko01110 Biosynthesis of secondary metabolites (7)	ko:K00016 LDH; L-lactate dehydrogenase [EC:1.1.1.27]
	ko:K01513 ENPP1_3; ectonucleotide pyrophosphatase/phosphodiesterase family member 1/3 [EC:3.1.4.1 3.6.1.9]
	ko:K01623 ALDO; fructose-bisphosphate aldolase, class I [EC:4.1.2.13]
	ko:K01689 ENO; enolase [EC:4.2.1.11]
	ko:K01697 CBS; cystathionine beta-synthase [EC:4.2.1.22]
	ko:K01807 rpiA; ribose 5-phosphate isomerase A [EC:5.3.1.6]
	ko:K01834 PGAM; 2,3-bisphosphoglycerate-dependent phosphoglycerate mutase [EC:5.4.2.11]
ko01120 Microbial metabolism in diverse	ko:K00016 LDH; L-lactate dehydrogenase [EC:1.1.1.27]
	ko:K00157 AOX; aldehyde oxidase [EC:1.2.3.1]
	ko:K01623 ALDO; fructose-bisphosphate aldolase, class I [EC:4.1.2.13]
	ko:K01689 ENO; enolase [EC:4.2.1.11]
	ko:K01807 rpiA; ribose 5-phosphate isomerase A [EC:5.3.1.6]
	ko:K01834 PGAM; 2,3-bisphosphoglycerate-dependent phosphoglycerate mutase [EC:5.4.2.11]

Table S4 Cont.

KEGG Pathway	Mapped protein
ko01230 Biosynthesis of amino acids (5)	ko:K01623 ALDO; fructose-bisphosphate aldolase, class I [EC:4.1.2.13]<br>ko:K01689 ENO; enolase [EC:4.2.1.11]<br>ko:K01697 CBS; cystathionine beta-synthase [EC:4.2.1.22]<br>ko:K01807 rpiA; ribose 5-phosphate isomerase A [EC:5.3.1.6]<br>ko:K01834 PGAM; 2,3-bisphosphoglycerate-dependent phosphoglycerate mutase [EC:5.4.2.11]
ko01200 Carbon metabolism (4)	ko:K01623 ALDO; fructose-bisphosphate aldolase, class I [EC:4.1.2.13]<br>ko:K01689 ENO; enolase [EC:4.2.1.11]<br>ko:K01807 rpiA; ribose 5-phosphate isomerase A [EC:5.3.1.6]<br>ko:K01834 PGAM; 2,3-bisphosphoglycerate-dependent phosphoglycerate mutase [EC:5.4.2.11]
ko00010 Glycolysis / Gluconeogenesis (4)	ko:K00016 LDH; L-lactate dehydrogenase [EC:1.1.1.27]<br>ko:K01623 ALDO; fructose-bisphosphate aldolase, class I [EC:4.1.2.13]<br>ko:K01689 ENO; enolase [EC:4.2.1.11]<br>ko:K01834 PGAM; 2,3-bisphosphoglycerate-dependent phosphoglycerate mutase [EC:5.4.2.11]
ko04066 HIF-1 signaling pathway (4)	ko:K00016 LDH; L-lactate dehydrogenase [EC:1.1.1.27]<br>ko:K01623 ALDO; fructose-bisphosphate aldolase, class I [EC:4.1.2.13]<br>ko:K01689 ENO; enolase [EC:4.2.1.11]<br>ko:K21421 NOX2; NADPH oxidase 2 [EC:1.-.-.-]
ko00680 Methane metabolism (3)	ko:K01623 ALDO; fructose-bisphosphate aldolase, class I [EC:4.1.2.13]<br>ko:K01689 ENO; enolase [EC:4.2.1.11]<br>ko:K01834 PGAM; 2,3-bisphosphoglycerate-dependent phosphoglycerate mutase [EC:5.4.2.11]
ko05414 Dilated cardiomyopathy (3)	ko:K06587 ITGA11; integrin alpha 11<br>ko:K07610 DES; desmin<br>ko:K12045 TNNT2; troponin T, cardiac muscle
ko05410 Hypertrophic cardiomyopathy (3)	ko:K06587 ITGA11; integrin alpha 11<br>ko:K07610 DES; desmin<br>ko:K12045 TNNT2; troponin T, cardiac muscle
ko04151 PI3K-Akt signaling pathway (3)	ko:K02583 NGFR; tumor necrosis factor receptor superfamily member 16<br>ko:K04659 THBS2S; thrombospondin 2/3/4/5<br>ko:K06587 ITGA11; integrin alpha 11

Table S4 Cont.

KEGG Pathway	Mapped protein
ko04145 Phagosome (3)	ko:K04659 THBS2S; thrombospondin 2/3/4/5<br>ko:K06752 MHC2; MHC class II antigen<br>ko:K21421 NOX2; NADPH oxidase 2 [EC:1.-.-.-]
ko00260 Glycine, serine and threonine metabolism (3)	ko:K01697 CBS; cystathionine beta-synthase [EC:4.2.1.22]<br>ko:K01834 PGAM; 2,3-bisphosphoglycerate-dependent phosphoglycerate mutase [EC:5.4.2.11]<br>ko:K15789 TDH; threonine 3-dehydrogenase [EC:1.1.1.103]
ko05152 Tuberculosis (2)	ko:K06505 CD74; CD74 antigen<br>ko:K06752 MHC2; MHC class II antigen
ko04512 ECM-receptor interaction (2)	ko:K04659 THBS2S; thrombospondin 2/3/4/5<br>ko:K06587 ITGA11; integrin alpha 11
ko04360 Axon guidance (2)	ko:K06850 SLIT3; slit 3<br>ko:K23096 RGMA; repulsive guidance molecule A
ko00760 Nicotinate and nicotinamide metabolism (2)	ko:K00157 AOX; aldehyde oxidase [EC:1.2.3.1]<br>ko:K01513 ENPP1_3; ectonucleotide pyrophosphatase/phosphodiesterase family member 1/3 [EC:3.1.4.1 3.6.1.9]
ko04514 Cell adhesion molecules (2)	ko:K06494 SELE; selectin, endothelial cell<br>ko:K06752 MHC2; MHC class II antigen
ko05418 Fluid shear stress and atherosclerosis (2)	ko:K00799 GST; glutathione S-transferase [EC:2.5.1.18]<br>ko:K06494 SELE; selectin, endothelial cell
ko00710 Carbon fixation	ko:K01623 ALDO; fructose-bisphosphate aldolase, class I [EC:4.1.2.13]<br>ko:K01807 rpiA; ribose 5-phosphate isomerase A [EC:5.3.1.6]
ko04640 Hematopoietic cell lineage (2)	ko:K01389 MME; neprilysin [EC:3.4.24.11]<br>ko:K06752 MHC2; MHC class II antigen
ko00270 Cysteine and methionine metabolism (2)	ko:K00016 LDH; L-lactate dehydrogenase [EC:1.1.1.27]<br>ko:K01697 CBS; cystathionine beta-synthase [EC:4.2.1.22]
ko05168 Herpes simplex virus 1 infection (2)	ko:K06505 CD74; CD74 antigen<br>ko:K06752 MHC2; MHC class II antigen

Table S4 Cont.

KEGG Pathway	Mapped protein
ko05415 Diabetic cardiomyopathy (2)	ko:K00820 glmS; glutamine---fructose-6-phosphate transaminase (isomerizing) [EC:2.6.1.16]<br>ko:K21421 NOX2; NADPH oxidase 2 [EC:1.-.-.-]
ko00030 Pentose phosphate pathway (2)	ko:K01623 ALDO; fructose-bisphosphate aldolase, class I [EC:4.1.2.13]<br>ko:K01807 rpiA; ribose 5-phosphate isomerase A [EC:5.3.1.6]
ko05144 Malaria (2)	ko:K04659 THBS2S; thrombospondin 2/3/4/5<br>ko:K06494 SELE; selectin, endothelial cell
ko05412 Arrhythmogenic	ko:K06587 ITGA11; integrin alpha 11<br>ko:K07610 DES; desmin
ko05417 Lipid and atherosclerosis (2)	ko:K06494 SELE; selectin, endothelial cell<br>ko:K21421 NOX2; NADPH oxidase 2 [EC:1.-.-.-]
ko05010 Alzheimer disease (2)	ko:K01389 MME; neprilysin [EC:3.4.24.11]<br>ko:K21421 NOX2; NADPH oxidase 2 [EC:1.-.-.-]
ko04922 Glucagon signaling pathway (2)	ko:K00016 LDH; L-lactate dehydrogenase [EC:1.1.1.27]<br>ko:K01834 PGAM; 2,3-bisphosphoglycerate-dependent phosphoglycerate mutase [EC:5.4.2.11]
ko05230 Central carbon metabolism in cancer (2)	ko:K00016 LDH; L-lactate dehydrogenase [EC:1.1.1.27]<br>ko:K01834 PGAM; 2,3-bisphosphoglycerate-dependent phosphoglycerate mutase [EC:5.4.2.11]
ko05165 Human papillomavirus infection (2)	ko:K04659 THBS2S; thrombospondin 2/3/4/5<br>ko:K06587 ITGA11; integrin alpha 11
ko04976 Bile secretion (2)	ko:K09866 AQP4; aquaporin-4<br>ko:K18245 CA2; carbonic anhydrase 2 [EC:4.2.1.1]
ko04510 Focal adhesion (2)	ko:K04659 THBS2S; thrombospondin 2/3/4/5<br>ko:K06587 ITGA11; integrin alpha 11
ko00982 Drug metabolism - cytochrome P450 (2)	ko:K00157 AOX; aldehyde oxidase [EC:1.2.3.1]<br>ko:K00799 GST; glutathione S-transferase [EC:2.5.1.18]
