# Supplementary material for: Thermal impacts on transcriptome of Pectoralis major muscle collected from commercial broilers, Thai native chickens and its crossbreeds
Source: Anim Biosci. 2023 Oct 31;37(1):61–73. doi: 10.5713/ab.23.0195 (PMC10766454; doi:10.5713/ab.23.0195)
Supplement: Supplementary file 6 [file ab-23-0195-Supplementary-Table-5.pdf]

Table S5 KEGG pathways associated with thermal stress within Thai native chickens

KEGG Pathway	Mapped protein
ko01100 Metabolic pathways (13)	ko:K00016 LDH; L-lactate dehydrogenase [EC:1.1.1.27]
	ko:K00710 GALNT; polypeptide N-acetylgalactosaminyltransferase [EC:2.4.1.41]
	ko:K00927 PGK; phosphoglycerate kinase [EC:2.7.2.3]
	ko:K00933 E2.7.3.2; creatine kinase [EC:2.7.3.2]
	ko:K00939 adk; adenylate kinase [EC:2.7.4.3]
	ko:K01061 E3.1.1.45; carboxymethylenebutenolidase [EC:3.1.1.45]
	ko:K01803 TPI; triosephosphate isomerase (TIM) [EC:5.3.1.1]
	ko:K01834 PGAM; 2,3-bisphosphoglycerate-dependent phosphoglycerate mutase [EC:5.4.2.11]
	ko:K01915 glnA; glutamine synthetase [EC:6.3.1.2]
	ko:K02126 ATPeF0A; F-type H+-transporting ATPase subunit a
	ko:K02261 COX2; cytochrome c oxidase subunit 2
	ko:K02262 COX3; cytochrome c oxidase subunit 3
	ko:K10703 HACD; very-long-chain (3R)-3-hydroxyacyl-CoA dehydratase [EC:4.2.1.134]
ko01120 Microbial metabolism in diverse	ko:K00016 LDH; L-lactate dehydrogenase [EC:1.1.1.27]
	ko:K01061 E3.1.1.45; carboxymethylenebutenolidase [EC:3.1.1.45]
	ko:K01803 TPI; triosephosphate isomerase (TIM) [EC:5.3.1.1]
	ko:K01834 PGAM; 2,3-bisphosphoglycerate-dependent phosphoglycerate mutase [EC:5.4.2.11]
	ko:K01915 glnA; glutamine synthetase [EC:6.3.1.2]
ko05171 Coronavirus disease - COVID-19 (6)	ko:K01330 C1R; complement component 1, r subcomponent [EC:3.4.21.41]
	ko:K02880 RP-L17e; large subunit ribosomal protein L17e
	ko:K02969 RP-S20e; small subunit ribosomal protein S20e
	ko:K02971 RP-S21e; small subunit ribosomal protein S21e
	ko:K03917 F13A1; coagulation factor XIII A1 polypeptide [EC:2.3.2.13]
	ko:K03996 C7; complement component 7

Table S5 Cont.

KEGG Pathway	Mapped protein
ko01110 Biosynthesis of secondary metabolites (6)	ko:K00016 LDH; L-lactate dehydrogenase [EC:1.1.1.27]<br>ko:K00927 PGK; phosphoglycerate kinase [EC:2.7.2.3]<br>ko:K00939 adk; adenylate kinase [EC:2.7.4.3]<br>ko:K01803 TPI; triosephosphate isomerase (TIM) [EC:5.3.1.1]<br>ko:K01834 PGAM; 2,3-bisphosphoglycerate-dependent phosphoglycerate mutase [EC:5.4.2.11]<br>ko:K10703 HACD; very-long-chain (3R)-3-hydroxyacyl-CoA dehydratase [EC:4.2.1.134]
ko04510 Focal adhesion (5)	ko:K05449 VEGFC_D; vascular endothelial growth factor C/D<br>ko:K05735 PAK6; p21-activated kinase 6 [EC:2.7.11.1]<br>ko:K06240 LAMA3_5; laminin, alpha 3/5<br>ko:K06250 SPP1; secreted phosphoprotein 1<br>ko:K12755 MYL9; myosin regulatory light chain 9
ko04810 Regulation of actin cytoskeleton (5)	ko:K03996 C7; complement component 7<br>ko:K04358 FGF; fibroblast growth factor<br>ko:K05735 PAK6; p21-activated kinase 6 [EC:2.7.11.1]<br>ko:K10352 MYH9s; myosin heavy chain 9/10/11/14<br>ko:K12755 MYL9; myosin regulatory light chain 9
ko05012 Parkinson disease (4)	ko:K02126 ATPeF0A; F-type H+-transporting ATPase subunit a<br>ko:K02261 COX2; cytochrome c oxidase subunit 2<br>ko:K02262 COX3; cytochrome c oxidase subunit 3<br>ko:K04551 UBB; ubiquitin B
ko05022 Pathways of neurodegeneration - multiple diseases (4)	ko:K02126 ATPeF0A; F-type H+-transporting ATPase subunit a<br>ko:K02261 COX2; cytochrome c oxidase subunit 2<br>ko:K02262 COX3; cytochrome c oxidase subunit 3<br>ko:K04551 UBB; ubiquitin B
ko04151 PI3K-Akt signaling pathway (4)	ko:K04358 FGF; fibroblast growth factor<br>ko:K05449 VEGFC_D; vascular endothelial growth factor C/D<br>ko:K06240 LAMA3_5; laminin, alpha 3/5<br>ko:K06250 SPP1; secreted phosphoprotein 1

Table S5 Cont.

KEGG Pathway	Mapped protein
ko01230 Biosynthesis of amino acids (4)	ko:K00927 PGK; phosphoglycerate kinase [EC:2.7.2.3]<br>ko:K01803 TPI; triosephosphate isomerase (TIM) [EC:5.3.1.1]<br>ko:K01834 PGAM; 2,3-bisphosphoglycerate-dependent phosphoglycerate mutase [EC:5.4.2.11]<br>ko:K01915 glnA; glutamine synthetase [EC:6.3.1.2]
ko00010 Glycolysis / Gluconeogenesis (4)	ko:K00016 LDH; L-lactate dehydrogenase [EC:1.1.1.27]<br>ko:K00927 PGK; phosphoglycerate kinase [EC:2.7.2.3]<br>ko:K01803 TPI; triosephosphate isomerase (TIM) [EC:5.3.1.1]<br>ko:K01834 PGAM; 2,3-bisphosphoglycerate-dependent phosphoglycerate mutase [EC:5.4.2.11]
ko05020 Prion disease (4)	ko:K02126 ATPeF0A; F-type H+-transporting ATPase subunit a<br>ko:K02261 COX2; cytochrome c oxidase subunit 2<br>ko:K02262 COX3; cytochrome c oxidase subunit 3<br>ko:K03996 C7; complement component 7
ko05016 Huntington disease (3)	ko:K02126 ATPeF0A; F-type H+-transporting ATPase subunit a<br>ko:K02261 COX2; cytochrome c oxidase subunit 2<br>ko:K02262 COX3; cytochrome c oxidase subunit 3
ko04066 HIF-1 signaling pathway (3)	ko:K00016 LDH; L-lactate dehydrogenase [EC:1.1.1.27]<br>ko:K00927 PGK; phosphoglycerate kinase [EC:2.7.2.3]<br>ko:K14736 TF; transferrin
ko05200 Pathways in cancer (3)	ko:K04358 FGF; fibroblast growth factor<br>ko:K05449 VEGFC_D; vascular endothelial growth factor C/D<br>ko:K06240 LAMA3_5; laminin, alpha 3/5
ko00190 Oxidative phosphorylation (3)	ko:K02126 ATPeF0A; F-type H+-transporting ATPase subunit a<br>ko:K02261 COX2; cytochrome c oxidase subunit 2<br>ko:K02262 COX3; cytochrome c oxidase subunit 3
ko01200 Carbon metabolism (3)	ko:K00927 PGK; phosphoglycerate kinase [EC:2.7.2.3]<br>ko:K01803 TPI; triosephosphate isomerase (TIM) [EC:5.3.1.1]<br>ko:K01834 PGAM; 2,3-bisphosphoglycerate-dependent phosphoglycerate mutase [EC:5.4.2.11]
ko05014 Amyotrophic lateral sclerosis (3)	ko:K02126 ATPeF0A; F-type H+-transporting ATPase subunit a<br>ko:K02261 COX2; cytochrome c oxidase subunit 2<br>ko:K02262 COX3; cytochrome c oxidase subunit 3

Table S5 Cont.

KEGG Pathway	Mapped protein
ko04014 Ras signaling pathway (3)	ko:K04358 FGF; fibroblast growth factor<br>ko:K05449 VEGFC_D; vascular endothelial growth factor C/D<br>ko:K05735 PAK6; p21-activated kinase 6 [EC:2.7.11.1]
ko03010 Ribosome (3)	ko:K02880 RP-L17e; large subunit ribosomal protein L17e<br>ko:K02969 RP-S20e; small subunit ribosomal protein S20e<br>ko:K02971 RP-S21e; small subunit ribosomal protein S21e
ko04714 Thermogenesis (3)	ko:K02126 ATPeF0A; F-type H+-transporting ATPase subunit a<br>ko:K02261 COX2; cytochrome c oxidase subunit 2<br>ko:K02262 COX3; cytochrome c oxidase subunit 3
ko04270 Vascular smooth muscle contraction (3)	ko:K10352 MYH9s; myosin heavy chain 9/10/11/14<br>ko:K12315 ACTG2; actin, gamma-enteric smooth muscle<br>ko:K12755 MYL9; myosin regulatory light chain 9
ko04610 Complement and coagulation cascades (3)	ko:K01330 C1R; complement component 1, r subcomponent [EC:3.4.21.41]<br>ko:K03917 F13A1; coagulation factor XIII A1 polypeptide [EC:2.3.2.13]<br>ko:K03996 C7; complement component 7
ko05208 Chemical carcinogenesis - reactive oxygen species (3)	ko:K02126 ATPeF0A; F-type H+-transporting ATPase subunit a<br>ko:K02261 COX2; cytochrome c oxidase subunit 2<br>ko:K02262 COX3; cytochrome c oxidase subunit 3
ko04360 Axon guidance (3)	ko:K05735 PAK6; p21-activated kinase 6 [EC:2.7.11.1]<br>ko:K06845 NTN4; netrin 4<br>ko:K12755 MYL9; myosin regulatory light chain 9
ko04020 Calcium signaling pathway (3)	ko:K04211 NTSR1; neurotensin receptor 1<br>ko:K04358 FGF; fibroblast growth factor<br>ko:K05449 VEGFC_D; vascular endothelial growth factor C/D
ko05415 Diabetic cardiomyopathy (3)	ko:K02126 ATPeF0A; F-type H+-transporting ATPase subunit a<br>ko:K02261 COX2; cytochrome c oxidase subunit 2<br>ko:K02262 COX3; cytochrome c oxidase subunit 3
ko05010 Alzheimer disease (3)	ko:K02126 ATPeF0A; F-type H+-transporting ATPase subunit a<br>ko:K02261 COX2; cytochrome c oxidase subunit 2<br>ko:K02262 COX3; cytochrome c oxidase subunit 3

Table S5 Cont.

KEGG Pathway	Mapped protein
ko05131 Shigellosis (2)	ko:K04551 UBB; ubiquitin B<br>ko:K12755 MYL9; myosin regulatory light chain 9
ko04922 Glucagon signaling pathway (2)	ko:K00016 LDH; L-lactate dehydrogenase [EC:1.1.1.27]<br>ko:K01834 PGAM; 2,3-bisphosphoglycerate-dependent phosphoglycerate mutase [EC:5.4.2.11]
ko00710 Carbon fixation in photosynthetic organisms (2)	ko:K00927 PGK; phosphoglycerate kinase [EC:2.7.2.3]<br>ko:K01803 TPI; triosephosphate isomerase (TIM) [EC:5.3.1.1]
ko05322 Systemic lupus erythematosus (2)	ko:K01330 C1R; complement component 1, r subcomponent [EC:3.4.21.41]<br>ko:K03996 C7; complement component 7
ko05165 Human papillomavirus infection (2)	ko:K06240 LAMA3_5; laminin, alpha 3/5<br>ko:K06250 SPP1; secreted phosphoprotein 1
ko04974 Protein digestion and absorption (2)	ko:K08132 COL12A; collagen type XII alpha<br>ko:K14211 ELN; elastin
ko04530 Tight junction (2)	ko:K10352 MYH9s; myosin heavy chain 9/10/11/14<br>ko:K12755 MYL9; myosin regulatory light chain 9
ko04512 ECM-receptor interaction (2)	ko:K06240 LAMA3_5; laminin, alpha 3/5<br>ko:K06250 SPP1; secreted phosphoprotein 1
ko04010 MAPK signaling pathway (2)	ko:K04358 FGF; fibroblast growth factor<br>ko:K05449 VEGFC_D; vascular endothelial growth factor C/D
ko05230 Central carbon metabolism in cancer (2)	ko:K00016 LDH; L-lactate dehydrogenase [EC:1.1.1.27]<br>ko:K01834 PGAM; 2,3-bisphosphoglycerate-dependent phosphoglycerate mutase [EC:5.4.2.11]
ko04260 Cardiac muscle contraction (2)	ko:K02261 COX2; cytochrome c oxidase subunit 2<br>ko:K02262 COX3; cytochrome c oxidase subunit 3
ko04015 Rap1 signaling pathway (2)	ko:K04358 FGF; fibroblast growth factor<br>ko:K05449 VEGFC_D; vascular endothelial growth factor C/D
ko04932 Non-alcoholic fatty liver disease (2)	ko:K02261 COX2; cytochrome c oxidase subunit 2<br>ko:K02262 COX3; cytochrome c oxidase subunit 3
