# Supplementary material for: Thermal impacts on transcriptome of Pectoralis major muscle collected from commercial broilers, Thai native chickens and its crossbreeds
Source: Anim Biosci. 2023 Oct 31;37(1):61–73. doi: 10.5713/ab.23.0195 (PMC10766454; doi:10.5713/ab.23.0195)
Supplement: Supplementary file 7 [file ab-23-0195-Supplementary-Table-6.pdf]

Table S6 KEGG pathways associated with thermal stress within crossbred H75

KEGG Pathway	Mapped protein
ko01100 Metabolic pathways (35)	ko:K00016 LDH; L-lactate dehydrogenase [EC:1.1.1.27]
	ko:K00251 AKR1D1; 3-oxo-5-beta-steroid 4-dehydrogenase [EC:1.3.1.3]
	ko:K00286 proC; pyrroline-5-carboxylate reductase [EC:1.5.1.2]
	ko:K00613 GATM; glycine amidinotransferase [EC:2.1.4.1]
	ko:K00850 pfkA; 6-phosphofructokinase 1 [EC:2.7.1.11]
	ko:K00873 PK; pyruvate kinase [EC:2.7.1.40]
	ko:K00933 E2.7.3.2; creatine kinase [EC:2.7.3.2]
	ko:K00939 adk; adenylate kinase [EC:2.7.4.3]
	ko:K01068 ACOT1_2_4; acyl-coenzyme A thioesterase 1/2/4 [EC:3.1.2.2]
	ko:K01092 E3.1.3.25; myo-inositol-1(or 4)-monophosphatase [EC:3.1.3.25]
	ko:K01230 MAN1A_C; mannosyl-oligosaccharide alpha-1,2-mannosidase [EC:3.2.1.113]
	ko:K01511 ENTPD5_6; ectonucleoside triphosphate diphosphohydrolase 5/6 [EC:3.6.1.6]
	ko:K01623 ALDO; fructose-bisphosphate aldolase, class I [EC:4.1.2.13]
	ko:K01672 CA; carbonic anhydrase [EC:4.2.1.1]
	ko:K01689 ENO; enolase [EC:4.2.1.11]
	ko:K01803 TPI; triosephosphate isomerase (TIM) [EC:5.3.1.1]
	ko:K01835 pgm; phosphoglucomutase [EC:5.4.2.2]
	ko:K01837 BPGM; bisphosphoglycerate/phosphoglycerate mutase [EC:5.4.2.4 5.4.2.11]
	ko:K01939 purA; adenylosuccinate synthase [EC:6.3.4.4]
	ko:K01946 ACACB; acetyl-CoA carboxylase / biotin carboxylase 2 [EC:6.4.1.2 6.3.4.14 2.1.3.15]
	ko:K03884 ND6; NADH-ubiquinone oxidoreductase chain 6 [EC:7.1.1.2]
	ko:K05290 PIGK; GPI-anchor transamidase subunit K
	ko:K07023 YGK1; 5'-deoxynucleotidase [EC:3.1.3.89]
	ko:K07249 ALDH1A; retinal dehydrogenase [EC:1.2.1.36]
	ko:K07541 PIGX; GPI mannosyltransferase 1 subunit X
	ko:K08726 EPHX2; soluble epoxide hydrolase / lipid-phosphate phosphatase [EC:3.3.2.10 3.1.3.76]
	ko:K08730 PTDSS2; phosphatidylserine synthase 2 [EC:2.7.8.29]
	ko:K09188 MLL3; [histone H3]-lysine4 N-trimethyltransferase MLL3 [EC:2.1.1.354]

Table S6 Cont.

KEGG Pathway	Mapped protein
ko01100 Metabolic pathways (cont.)	ko:K09680 PANK1_2_3; type II pantothenate kinase [EC:2.7.1.33]<br>ko:K11412 SIRT2; NAD+-dependent protein deacetylase sirtuin 2 [EC:2.3.1.286]<br>ko:K11540 CAD; carbamoyl-phosphate synthase / aspartate carbamoyltransferase / dihydroorotase [EC:6.3.5.5 2.1.3.2 3.5.2.3]<br>ko:K18436 PDE7; high affinity cAMP-specific 3',5'-cyclic phosphodiesterase 7 [EC:3.1.4.53]<br>ko:K19572 CECR1; adenosine deaminase CECR1 [EC:3.5.4.4]<br>ko:K20967 MOCS1; GTP 3',8-cyclase / cyclic pyranopterin monophosphate synthase [EC:4.1.99.22 4.6.1.17]<br>ko:K22437 E2.1.1.22; carnosine N-methyltransferase [EC:2.1.1.22]
ko01110 Biosynthesis of secondary metabolites (14)	ko:K00016 LDH; L-lactate dehydrogenase [EC:1.1.1.27]<br>ko:K00286 proC; pyrroline-5-carboxylate reductase [EC:1.5.1.2]<br>ko:K00850 pfkA; 6-phosphofructokinase 1 [EC:2.7.1.11]<br>ko:K00873 PK; pyruvate kinase [EC:2.7.1.40]<br>ko:K00939 adk; adenylate kinase [EC:2.7.4.3]<br>ko:K01068 ACOT1_2_4; acyl-coenzyme A thioesterase 1/2/4 [EC:3.1.2.2]<br>ko:K01092 E3.1.3.25; myo-inositol-1(or 4)-monophosphatase [EC:3.1.3.25]<br>ko:K01623 ALDO; fructose-bisphosphate aldolase, class I [EC:4.1.2.13]<br>ko:K01689 ENO; enolase [EC:4.2.1.11]<br>ko:K01803 TPI; triosephosphate isomerase (TIM) [EC:5.3.1.1]<br>ko:K01835 pgm; phosphoglucomutase [EC:5.4.2.2]<br>ko:K01837 BPGM; bisphosphoglycerate/phosphoglycerate mutase [EC:5.4.2.4 5.4.2.11]<br>ko:K01946 ACACB; acetyl-CoA carboxylase / biotin carboxylase 2 [EC:6.4.1.2 6.3.4.14 2.1.3.15]<br>ko:K08730 PTDSS2; phosphatidylserine synthase 2 [EC:2.7.8.29]
ko04010 MAPK signaling pathway (10)	ko:K04346 GNA12; guanine nucleotide-binding protein subunit alpha-12<br>ko:K04358 FGF; fibroblast growth factor<br>ko:K04380 MAPT; microtubule-associated protein tau<br>ko:K04405 ECSIT; evolutionarily conserved signaling intermediate in Toll pathway<br>ko:K04410 PAK2; p21-activated kinase 2 [EC:2.7.11.1]<br>ko:K04429 TAO; thousand and one amino acid protein kinase [EC:2.7.11.1]<br>ko:K04431 MAP2K7; mitogen-activated protein kinase kinase 7 [EC:2.7.12.2]<br>ko:K04433 MAP2K6; mitogen-activated protein kinase kinase 6 [EC:2.7.12.2]

Table S6 Cont.

KEGG Pathway	Mapped protein
ko04010 MAPK signaling pathway (cont.)	ko:K05089 PDGFRB; platelet-derived growth factor receptor beta [EC:2.7.10.1]<br>ko:K05096 FLT1; FMS-like tyrosine kinase 1 [EC:2.7.10.1]
ko04140 Autophagy - animal (8)	ko:K02649 PIK3R1_2_3; phosphoinositide-3-kinase regulatory subunit alpha/beta/delta<br>ko:K07187 IRS2; insulin receptor substrate 2<br>ko:K07198 PRKAA; 5'-AMP-activated protein kinase, catalytic alpha subunit [EC:2.7.11.11]<br>ko:K08269 ULK2; serine/threonine-protein kinase ULK2 [EC:2.7.11.1]<br>ko:K08803 DAPK; death-associated protein kinase [EC:2.7.11.1]<br>ko:K18052 PRKCQ; novel protein kinase C theta type [EC:2.7.11.13]<br>ko:K20402 DEPTOR; DEP domain-containing mTOR-interacting protein<br>ko:K21249 UVRAG; UV radiation resistance-associated gene protein
ko00010 Glycolysis / Gluconeogenesis (8)	ko:K00016 LDH; L-lactate dehydrogenase [EC:1.1.1.27]<br>ko:K00850 pfkA; 6-phosphofructokinase 1 [EC:2.7.1.11]<br>ko:K00873 PK; pyruvate kinase [EC:2.7.1.40]<br>ko:K01623 ALDO; fructose-bisphosphate aldolase, class I [EC:4.1.2.13]<br>ko:K01689 ENO; enolase [EC:4.2.1.11]<br>ko:K01803 TPI; triosephosphate isomerase (TIM) [EC:5.3.1.1]<br>ko:K01835 pgm; phosphoglucomutase [EC:5.4.2.2]<br>ko:K01837 BPGM; bisphosphoglycerate/phosphoglycerate mutase [EC:5.4.2.4 5.4.2.11]
ko04810 Regulation of actin cytoskeleton (8)	ko:K02649 PIK3R1_2_3; phosphoinositide-3-kinase regulatory subunit alpha/beta/delta<br>ko:K04346 GNA12; guanine nucleotide-binding protein subunit alpha-12<br>ko:K04358 FGF; fibroblast growth factor<br>ko:K04410 PAK2; p21-activated kinase 2 [EC:2.7.11.1]<br>ko:K05089 PDGFRB; platelet-derived growth factor receptor beta [EC:2.7.10.1]<br>ko:K05766 SSH; protein phosphatase slingshot [EC:3.1.3.16 3.1.3.48]<br>ko:K12757 MYL12; myosin regulatory light chain 12<br>ko:K12758 MYLPF; fast skeletal myosin light chain 2
ko05417 Lipid and atherosclerosis (7)	ko:K02649 PIK3R1_2_3; phosphoinositide-3-kinase regulatory subunit alpha/beta/delta<br>ko:K04079 HSP90A; molecular chaperone HtpG<br>ko:K04431 MAP2K7; mitogen-activated protein kinase kinase 7 [EC:2.7.12.2]<br>ko:K04433 MAP2K6; mitogen-activated protein kinase kinase 6 [EC:2.7.12.2]

Table S6 Cont.

KEGG Pathway	Mapped protein
ko05417 Lipid and atherosclerosis (cont.)	ko:K04734 NFKBIA; NF-kappa-B inhibitor alpha<br>ko:K06494 SELE; selectin, endothelial cell<br>ko:K06496 SELP; selectin, platelet
ko04931 Insulin resistance (7)	ko:K01946 ACACB; acetyl-CoA carboxylase / biotin carboxylase 2 [EC:6.4.1.2 6.3.4.14 2.1.3.15]<br>ko:K02649 PIK3R1_2_3; phosphoinositide-3-kinase regulatory subunit alpha/beta/delta<br>ko:K04734 NFKBIA; NF-kappa-B inhibitor alpha<br>ko:K07187 IRS2; insulin receptor substrate 2<br>ko:K07198 PRKAA; 5'-AMP-activated protein kinase, catalytic alpha subunit [EC:2.7.11.11]<br>ko:K15719 NCOAT; protein O-GlcNAcase / histone acetyltransferase [EC:3.2.1.169 2.3.1.48]<br>ko:K18052 PRKCQ; novel protein kinase C theta type [EC:2.7.11.13]
ko04510 Focal adhesion (7)	ko:K02649 PIK3R1_2_3; phosphoinositide-3-kinase regulatory subunit alpha/beta/delta<br>ko:K04410 PAK2; p21-activated kinase 2 [EC:2.7.11.1]<br>ko:K05089 PDGFRB; platelet-derived growth factor receptor beta [EC:2.7.10.1]<br>ko:K05096 FLT1; FMS-like tyrosine kinase 1 [EC:2.7.10.1]<br>ko:K06250 SPP1; secreted phosphoprotein 1<br>ko:K12757 MYL12; myosin regulatory light chain 12<br>ko:K12758 MYLPF; fast skeletal myosin light chain 2
ko04151 PI3K-Akt signaling pathway (7)	ko:K02649 PIK3R1_2_3; phosphoinositide-3-kinase regulatory subunit alpha/beta/delta<br>ko:K04079 HSP90A; molecular chaperone HtpG<br>ko:K04358 FGF; fibroblast growth factor<br>ko:K05089 PDGFRB; platelet-derived growth factor receptor beta [EC:2.7.10.1]<br>ko:K05096 FLT1; FMS-like tyrosine kinase 1 [EC:2.7.10.1]<br>ko:K06250 SPP1; secreted phosphoprotein 1<br>ko:K07198 PRKAA; 5'-AMP-activated protein kinase, catalytic alpha subunit [EC:2.7.11.11]
ko00230 Purine metabolism (7)	ko:K00939 adk; adenylate kinase [EC:2.7.4.3]<br>ko:K01511 ENTPD5_6; ectonucleoside triphosphate diphosphohydrolase 5/6 [EC:3.6.1.6]<br>ko:K01835 pgm; phosphoglucomutase [EC:5.4.2.2]<br>ko:K01939 purA; adenylosuccinate synthase [EC:6.3.4.4]<br>ko:K07023 YGK1; 5'-deoxynucleotidase [EC:3.1.3.89]<br>ko:K18436 PDE7; high affinity cAMP-specific 3',5'-cyclic phosphodiesterase 7 [EC:3.1.4.53]

Table S6 Cont.

KEGG Pathway	Mapped protein
ko00230 Purine metabolism (cont.)	ko:K19572 CECR1; adenosine deaminase CECR1 [EC:3.5.4.4]
ko04066 HIF-1 signaling pathway (6)	ko:K00016 LDH; L-lactate dehydrogenase [EC:1.1.1.27]<br>ko:K00850 pfkA; 6-phosphofructokinase 1 [EC:2.7.1.11]<br>ko:K01623 ALDO; fructose-bisphosphate aldolase, class I [EC:4.1.2.13]<br>ko:K01689 ENO; enolase [EC:4.2.1.11]<br>ko:K02649 PIK3R1_2_3; phosphoinositide-3-kinase regulatory subunit alpha/beta/delta<br>ko:K05096 FLT1; FMS-like tyrosine kinase 1 [EC:2.7.10.1]
ko04014 Ras signaling pathway (6)	ko:K02649 PIK3R1_2_3; phosphoinositide-3-kinase regulatory subunit alpha/beta/delta<br>ko:K02678 ETS1; C-ets-1<br>ko:K04358 FGF; fibroblast growth factor<br>ko:K04410 PAK2; p21-activated kinase 2 [EC:2.7.11.1]<br>ko:K05089 PDGFRB; platelet-derived growth factor receptor beta [EC:2.7.10.1]<br>ko:K05096 FLT1; FMS-like tyrosine kinase 1 [EC:2.7.10.1]
ko05230 Central carbon metabolism in cancer (6)	ko:K00016 LDH; L-lactate dehydrogenase [EC:1.1.1.27]<br>ko:K00850 pfkA; 6-phosphofructokinase 1 [EC:2.7.1.11]<br>ko:K00873 PK; pyruvate kinase [EC:2.7.1.40]<br>ko:K02649 PIK3R1_2_3; phosphoinositide-3-kinase regulatory subunit alpha/beta/delta<br>ko:K05089 PDGFRB; platelet-derived growth factor receptor beta [EC:2.7.10.1]<br>ko:K05126 RET; proto-oncogene tyrosine-protein kinase Ret [EC:2.7.10.1]
ko01230 Biosynthesis of amino acids (6)	ko:K00286 proC; pyrroline-5-carboxylate reductase [EC:1.5.1.2]<br>ko:K00850 pfkA; 6-phosphofructokinase 1 [EC:2.7.1.11]<br>ko:K00873 PK; pyruvate kinase [EC:2.7.1.40]<br>ko:K01623 ALDO; fructose-bisphosphate aldolase, class I [EC:4.1.2.13]<br>ko:K01689 ENO; enolase [EC:4.2.1.11]<br>ko:K01803 TPI; triosephosphate isomerase (TIM) [EC:5.3.1.1]
ko04360 Axon guidance (6)	ko:K00444 WNT5; wingless-type MMTV integration site family, member 5<br>ko:K02649 PIK3R1_2_3; phosphoinositide-3-kinase regulatory subunit alpha/beta/delta<br>ko:K04410 PAK2; p21-activated kinase 2 [EC:2.7.11.1]<br>ko:K05766 SSH; protein phosphatase slingshot [EC:3.1.3.16 3.1.3.48]<br>ko:K06842 SEMA6; semaphorin 6

Table S6 Cont.

KEGG Pathway	Mapped protein
ko04360 Axon guidance (cont.)	ko:K12757 MYL12; myosin regulatory light chain 12
ko04152 AMPK signaling pathway (6)	ko:K00850 pfkA; 6-phosphofructokinase 1 [EC:2.7.1.11]<br>ko:K01946 ACACB; acetyl-CoA carboxylase / biotin carboxylase 2 [EC:6.4.1.2 6.3.4.14 2.1.3.15]<br>ko:K02649 PIK3R1_2_3; phosphoinositide-3-kinase regulatory subunit alpha/beta/delta<br>ko:K03234 EEF2; elongation factor 2<br>ko:K07187 IRS2; insulin receptor substrate 2<br>ko:K07198 PRKAA; 5'-AMP-activated protein kinase, catalytic alpha subunit [EC:2.7.11.11]
ko04380 Osteoclast differentiation (6)	ko:K02649 PIK3R1_2_3; phosphoinositide-3-kinase regulatory subunit alpha/beta/delta<br>ko:K04431 MAP2K7; mitogen-activated protein kinase kinase 7 [EC:2.7.12.2]<br>ko:K04433 MAP2K6; mitogen-activated protein kinase kinase 6 [EC:2.7.12.2]<br>ko:K04734 NFKBIA; NF-kappa-B inhibitor alpha<br>ko:K09030 FOSL2; fos-like antigen 2<br>ko:K14380 FHL2; four and a half LIM domains protein 2
ko05022 Pathways of neurodegeneration - multiple diseases (6)	ko:K00444 WNT5; wingless-type MMTV integration site family, member 5<br>ko:K03884 ND6; NADH-ubiquinone oxidoreductase chain 6 [EC:7.1.1.2]<br>ko:K04380 MAPT; microtubule-associated protein tau<br>ko:K04431 MAP2K7; mitogen-activated protein kinase kinase 7 [EC:2.7.12.2]<br>ko:K04433 MAP2K6; mitogen-activated protein kinase kinase 6 [EC:2.7.12.2]<br>ko:K08269 ULK2; serine/threonine-protein kinase ULK2 [EC:2.7.11.1]
ko05418 Fluid shear stress and atherosclerosis (6)	ko:K02649 PIK3R1_2_3; phosphoinositide-3-kinase regulatory subunit alpha/beta/delta<br>ko:K04079 HSP90A; molecular chaperone HtpG<br>ko:K04431 MAP2K7; mitogen-activated protein kinase kinase 7 [EC:2.7.12.2]<br>ko:K04433 MAP2K6; mitogen-activated protein kinase kinase 6 [EC:2.7.12.2]<br>ko:K06494 SELE; selectin, endothelial cell<br>ko:K07198 PRKAA; 5'-AMP-activated protein kinase, catalytic alpha subunit [EC:2.7.11.11]
ko04020 Calcium signaling pathway (6)	ko:K04358 FGF; fibroblast growth factor<br>ko:K05089 PDGFRB; platelet-derived growth factor receptor beta [EC:2.7.10.1]<br>ko:K05096 FLT1; FMS-like tyrosine kinase 1 [EC:2.7.10.1]<br>ko:K05126 RET; proto-oncogene tyrosine-protein kinase Ret [EC:2.7.10.1]<br>ko:K08794 CAMK1; calcium/calmodulin-dependent protein kinase I [EC:2.7.11.17]

Table S6 Cont.

KEGG Pathway	Mapped protein
ko04020 Calcium signaling pathway (cont.)	ko:K12042 TNNC2; troponin C, skeletal muscle
ko05207 Chemical carcinogenesis - receptor activation (6)	ko:K02649 PIK3R1_2_3; phosphoinositide-3-kinase regulatory subunit alpha/beta/delta<br>ko:K04079 HSP90A; molecular chaperone HtpG<br>ko:K04358 FGF; fibroblast growth factor<br>ko:K08726 EPHX2; soluble epoxide hydrolase / lipid-phosphate phosphatase [EC:3.3.2.10 3.1.3.76]<br>ko:K09206 KLF5; krueppel-like factor 5<br>ko:K25039 PAQR7; membrane progestin receptor alpha
ko04660 T cell receptor signaling pathway (5)	ko:K02649 PIK3R1_2_3; phosphoinositide-3-kinase regulatory subunit alpha/beta/delta<br>ko:K04410 PAK2; p21-activated kinase 2 [EC:2.7.11.1]<br>ko:K04431 MAP2K7; mitogen-activated protein kinase kinase 7 [EC:2.7.12.2]<br>ko:K04734 NFKBIA; NF-kappa-B inhibitor alpha<br>ko:K18052 PRKCQ; novel protein kinase C theta type [EC:2.7.11.13]
ko01240 Biosynthesis of cofactors (5)	ko:K00939 adk; adenylate kinase [EC:2.7.4.3]<br>ko:K01939 purA; adenylosuccinate synthase [EC:6.3.4.4]<br>ko:K09680 PANK1_2_3; type II pantothenate kinase [EC:2.7.1.33]<br>ko:K11540 CAD; carbamoyl-phosphate synthase / aspartate carbamoyltransferase / dihydroorotase [EC:6.3.5.5 2.1.3.2 3.5.2.3]<br>ko:K20967 MOCS1; GTP 3',8-cyclase / cyclic pyranopterin monophosphate synthase [EC:4.1.99.22 4.6.1.17]
ko04910 Insulin signaling pathway (5)	ko:K01946 ACACB; acetyl-CoA carboxylase / biotin carboxylase 2 [EC:6.4.1.2 6.3.4.14 2.1.3.15]<br>ko:K02649 PIK3R1_2_3; phosphoinositide-3-kinase regulatory subunit alpha/beta/delta<br>ko:K07187 IRS2; insulin receptor substrate 2<br>ko:K07193 SH2B; SH2B adapter protein 2<br>ko:K07198 PRKAA; 5'-AMP-activated protein kinase, catalytic alpha subunit [EC:2.7.11.11]
ko05170 Human immunodeficiency virus 1 infection (5)	ko:K02649 PIK3R1_2_3; phosphoinositide-3-kinase regulatory subunit alpha/beta/delta<br>ko:K04410 PAK2; p21-activated kinase 2 [EC:2.7.11.1]<br>ko:K04431 MAP2K7; mitogen-activated protein kinase kinase 7 [EC:2.7.12.2]<br>ko:K04433 MAP2K6; mitogen-activated protein kinase kinase 6 [EC:2.7.12.2]<br>ko:K04734 NFKBIA; NF-kappa-B inhibitor alpha

Table S6 Cont.

KEGG Pathway	Mapped protein
ko01200 Carbon metabolism (5)	ko:K00850 pfkA; 6-phosphofructokinase 1 [EC:2.7.1.11]<br>ko:K00873 PK; pyruvate kinase [EC:2.7.1.40]<br>ko:K01623 ALDO; fructose-bisphosphate aldolase, class I [EC:4.1.2.13]<br>ko:K01689 ENO; enolase [EC:4.2.1.11]<br>ko:K01803 TPI; triosephosphate isomerase (TIM) [EC:5.3.1.1]
ko05161 Hepatitis B (5)	ko:K02649 PIK3R1_2_3; phosphoinositide-3-kinase regulatory subunit alpha/beta/delta<br>ko:K04431 MAP2K7; mitogen-activated protein kinase kinase 7 [EC:2.7.12.2]<br>ko:K04433 MAP2K6; mitogen-activated protein kinase kinase 6 [EC:2.7.12.2]<br>ko:K04734 NFKBIA; NF-kappa-B inhibitor alpha<br>ko:K11594 DDX3X; ATP-dependent RNA helicase DDX3X [EC:3.6.4.13]
ko04150 mTOR signaling pathway (5)	ko:K00444 WNT5; wingless-type MMTV integration site family, member 5<br>ko:K02649 PIK3R1_2_3; phosphoinositide-3-kinase regulatory subunit alpha/beta/delta<br>ko:K07198 PRKAA; 5'-AMP-activated protein kinase, catalytic alpha subunit [EC:2.7.11.11]<br>ko:K08269 ULK2; serine/threonine-protein kinase ULK2 [EC:2.7.11.1]<br>ko:K20402 DEPTOR; DEP domain-containing mTOR-interacting protein
ko04620 Toll-like receptor signaling pathway (5)	ko:K02649 PIK3R1_2_3; phosphoinositide-3-kinase regulatory subunit alpha/beta/delta<br>ko:K04431 MAP2K7; mitogen-activated protein kinase kinase 7 [EC:2.7.12.2]<br>ko:K04433 MAP2K6; mitogen-activated protein kinase kinase 6 [EC:2.7.12.2]<br>ko:K04734 NFKBIA; NF-kappa-B inhibitor alpha<br>ko:K06250 SPP1; secreted phosphoprotein 1
ko05131 Shigellosis (5)	ko:K02649 PIK3R1_2_3; phosphoinositide-3-kinase regulatory subunit alpha/beta/delta<br>ko:K04734 NFKBIA; NF-kappa-B inhibitor alpha<br>ko:K12757 MYL12; myosin regulatory light chain 12<br>ko:K12758 MYLPF; fast skeletal myosin light chain 2<br>ko:K18052 PRKCQ; novel protein kinase C theta type [EC:2.7.11.13]
ko05208 Chemical carcinogenesis - reactive oxygen species (5)	ko:K02649 PIK3R1_2_3; phosphoinositide-3-kinase regulatory subunit alpha/beta/delta<br>ko:K03884 ND6; NADH-ubiquinone oxidoreductase chain 6 [EC:7.1.1.2]<br>ko:K04431 MAP2K7; mitogen-activated protein kinase kinase 7 [EC:2.7.12.2]<br>ko:K04734 NFKBIA; NF-kappa-B inhibitor alpha<br>ko:K08726 EPHX2; soluble epoxide hydrolase / lipid-phosphate phosphatase [EC:3.3.2.10 3.1.3.76]

Table S6 Cont.

KEGG Pathway	Mapped protein
ko04668 TNF signaling pathway (5)	ko:K02649 PIK3R1_2_3; phosphoinositide-3-kinase regulatory subunit alpha/beta/delta<br>ko:K04431 MAP2K7; mitogen-activated protein kinase kinase 7 [EC:2.7.12.2]<br>ko:K04433 MAP2K6; mitogen-activated protein kinase kinase 6 [EC:2.7.12.2]<br>ko:K04734 NFKBIA; NF-kappa-B inhibitor alpha<br>ko:K06494 SELE; selectin, endothelial cell
ko04936 Alcoholic liver disease (5)	ko:K01946 ACACB; acetyl-CoA carboxylase / biotin carboxylase 2 [EC:6.4.1.2 6.3.4.14 2.1.3.15]<br>ko:K04431 MAP2K7; mitogen-activated protein kinase kinase 7 [EC:2.7.12.2]<br>ko:K04433 MAP2K6; mitogen-activated protein kinase kinase 6 [EC:2.7.12.2]<br>ko:K04734 NFKBIA; NF-kappa-B inhibitor alpha<br>ko:K07198 PRKAA; 5'-AMP-activated protein kinase, catalytic alpha subunit [EC:2.7.11.11]
ko04920 Adipocytokine signaling pathway (5)	ko:K01946 ACACB; acetyl-CoA carboxylase / biotin carboxylase 2 [EC:6.4.1.2 6.3.4.14 2.1.3.15]<br>ko:K04734 NFKBIA; NF-kappa-B inhibitor alpha<br>ko:K07187 IRS2; insulin receptor substrate 2<br>ko:K07198 PRKAA; 5'-AMP-activated protein kinase, catalytic alpha subunit [EC:2.7.11.11]<br>ko:K18052 PRKCQ; novel protein kinase C theta type [EC:2.7.11.13]
ko04015 Rap1 signaling pathway (5)	ko:K02649 PIK3R1_2_3; phosphoinositide-3-kinase regulatory subunit alpha/beta/delta<br>ko:K04358 FGF; fibroblast growth factor<br>ko:K04433 MAP2K6; mitogen-activated protein kinase kinase 6 [EC:2.7.12.2]<br>ko:K05089 PDGFRB; platelet-derived growth factor receptor beta [EC:2.7.10.1]<br>ko:K05096 FLT1; FMS-like tyrosine kinase 1 [EC:2.7.10.1]
ko04922 Glucagon signaling pathway (5)	ko:K00016 LDH; L-lactate dehydrogenase [EC:1.1.1.27]<br>ko:K00850 pfkA; 6-phosphofructokinase 1 [EC:2.7.1.11]<br>ko:K00873 PK; pyruvate kinase [EC:2.7.1.40]<br>ko:K01946 ACACB; acetyl-CoA carboxylase / biotin carboxylase 2 [EC:6.4.1.2 6.3.4.14 2.1.3.15]<br>ko:K07198 PRKAA; 5'-AMP-activated protein kinase, catalytic alpha subunit [EC:2.7.11.11]
ko05165 Human papillomavirus infection (5)	ko:K00444 WNT5; wingless-type MMTV integration site family, member 5<br>ko:K00873 PK; pyruvate kinase [EC:2.7.1.40]<br>ko:K02649 PIK3R1_2_3; phosphoinositide-3-kinase regulatory subunit alpha/beta/delta<br>ko:K05089 PDGFRB; platelet-derived growth factor receptor beta [EC:2.7.10.1]<br>ko:K06250 SPP1; secreted phosphoprotein 1

Table S6 Cont.

KEGG Pathway	Mapped protein
ko01232 Nucleotide metabolism (5)	ko:K00939 adk; adenylate kinase [EC:2.7.4.3]<br>ko:K01511 ENTPD5_6; ectonucleoside triphosphate diphosphohydrolase 5/6 [EC:3.6.1.6]<br>ko:K01939 purA; adenylosuccinate synthase [EC:6.3.4.4]<br>ko:K07023 YGK1; 5'-deoxynucleotidase [EC:3.1.3.89]<br>ko:K19572 CECR1; adenosine deaminase CECR1 [EC:3.5.4.4]
ko05206 MicroRNAs in cancer (4)	ko:K02649 PIK3R1_2_3; phosphoinositide-3-kinase regulatory subunit alpha/beta/delta<br>ko:K05089 PDGFRB; platelet-derived growth factor receptor beta [EC:2.7.10.1]<br>ko:K07187 IRS2; insulin receptor substrate 2<br>ko:K23551 FSCN1; fascin 1
ko05215 Prostate cancer (4)	ko:K02649 PIK3R1_2_3; phosphoinositide-3-kinase regulatory subunit alpha/beta/delta<br>ko:K04079 HSP90A; molecular chaperone HtpG<br>ko:K04734 NFKBIA; NF-kappa-B inhibitor alpha<br>ko:K05089 PDGFRB; platelet-derived growth factor receptor beta [EC:2.7.10.1]
ko05169 Epstein-Barr virus infection (4)	ko:K02649 PIK3R1_2_3; phosphoinositide-3-kinase regulatory subunit alpha/beta/delta<br>ko:K04431 MAP2K7; mitogen-activated protein kinase kinase 7 [EC:2.7.12.2]<br>ko:K04433 MAP2K6; mitogen-activated protein kinase kinase 6 [EC:2.7.12.2]<br>ko:K04734 NFKBIA; NF-kappa-B inhibitor alpha
ko05163 Human cytomegalovirus infection (4)	ko:K02649 PIK3R1_2_3; phosphoinositide-3-kinase regulatory subunit alpha/beta/delta<br>ko:K04346 GNA12; guanine nucleotide-binding protein subunit alpha-12<br>ko:K04433 MAP2K6; mitogen-activated protein kinase kinase 6 [EC:2.7.12.2]<br>ko:K04734 NFKBIA; NF-kappa-B inhibitor alpha
ko04211 Longevity regulating pathway (4)	ko:K02649 PIK3R1_2_3; phosphoinositide-3-kinase regulatory subunit alpha/beta/delta<br>ko:K07187 IRS2; insulin receptor substrate 2<br>ko:K07198 PRKAA; 5'-AMP-activated protein kinase, catalytic alpha subunit [EC:2.7.11.11]<br>ko:K10141 SESN1_3; sestrin 1/3
ko05410 Hypertrophic cardiomyopathy (4)	ko:K07198 PRKAA; 5'-AMP-activated protein kinase, catalytic alpha subunit [EC:2.7.11.11]<br>ko:K12045 TNNT2; troponin T, cardiac muscle<br>ko:K12314 ACTC1; actin, alpha cardiac muscle<br>ko:K12567 TTN; titin [EC:2.7.11.1]

Table S6 Cont.

KEGG Pathway	Mapped protein
ko04068 FoxO signaling pathway (4)	ko:K02649 PIK3R1_2_3; phosphoinositide-3-kinase regulatory subunit alpha/beta/delta<br>ko:K07187 IRS2; insulin receptor substrate 2<br>ko:K07198 PRKAA; 5'-AMP-activated protein kinase, catalytic alpha subunit [EC:2.7.11.11]<br>ko:K15010 HOMER; homer
ko04218 Cellular senescence (4)	ko:K02649 PIK3R1_2_3; phosphoinositide-3-kinase regulatory subunit alpha/beta/delta<br>ko:K02678 ETS1; C-ets-1<br>ko:K04433 MAP2K6; mitogen-activated protein kinase kinase 6 [EC:2.7.12.2]<br>ko:K08826 HIPK; homeodomain interacting protein kinase [EC:2.7.11.1]
ko05014 Amyotrophic lateral sclerosis (4)	ko:K03884 ND6; NADH-ubiquinone oxidoreductase chain 6 [EC:7.1.1.2]<br>ko:K04433 MAP2K6; mitogen-activated protein kinase kinase 6 [EC:2.7.12.2]<br>ko:K08269 ULK2; serine/threonine-protein kinase ULK2 [EC:2.7.11.1]<br>ko:K16631 ANG; angiogenin [EC:3.1.27.-]
ko04722 Neurotrophin signaling pathway (4)	ko:K02649 PIK3R1_2_3; phosphoinositide-3-kinase regulatory subunit alpha/beta/delta<br>ko:K04431 MAP2K7; mitogen-activated protein kinase kinase 7 [EC:2.7.12.2]<br>ko:K04734 NFKBIA; NF-kappa-B inhibitor alpha<br>ko:K07193 SH2B; SH2B adapter protein 2
ko05135 Yersinia infection (4)	ko:K02649 PIK3R1_2_3; phosphoinositide-3-kinase regulatory subunit alpha/beta/delta<br>ko:K04431 MAP2K7; mitogen-activated protein kinase kinase 7 [EC:2.7.12.2]<br>ko:K04433 MAP2K6; mitogen-activated protein kinase kinase 6 [EC:2.7.12.2]<br>ko:K04734 NFKBIA; NF-kappa-B inhibitor alpha
ko05167 Kaposi sarcoma-associated herpesvirus infection (4)	ko:K02649 PIK3R1_2_3; phosphoinositide-3-kinase regulatory subunit alpha/beta/delta<br>ko:K04431 MAP2K7; mitogen-activated protein kinase kinase 7 [EC:2.7.12.2]<br>ko:K04433 MAP2K6; mitogen-activated protein kinase kinase 6 [EC:2.7.12.2]<br>ko:K04734 NFKBIA; NF-kappa-B inhibitor alpha
ko05203 Viral carcinogenesis (4)	ko:K00873 PK; pyruvate kinase [EC:2.7.1.40]<br>ko:K02649 PIK3R1_2_3; phosphoinositide-3-kinase regulatory subunit alpha/beta/delta<br>ko:K04734 NFKBIA; NF-kappa-B inhibitor alpha<br>ko:K11594 DDX3X; ATP-dependent RNA helicase DDX3X [EC:3.6.4.13]

Table S6 Cont.

KEGG Pathway	Mapped protein
ko04670 Leukocyte transendothelial migration (4)	ko:K02649 PIK3R1_2_3; phosphoinositide-3-kinase regulatory subunit alpha/beta/delta<br>ko:K07873 RHOH; Ras homolog gene family, member H<br>ko:K12757 MYL12; myosin regulatory light chain 12<br>ko:K12758 MYLPF; fast skeletal myosin light chain 2
ko05235 PD-L1 expression and PD-1 checkpoint pathway in cancer (4)	ko:K02649 PIK3R1_2_3; phosphoinositide-3-kinase regulatory subunit alpha/beta/delta<br>ko:K04433 MAP2K6; mitogen-activated protein kinase kinase 6 [EC:2.7.12.2]<br>ko:K04734 NFKBIA; NF-kappa-B inhibitor alpha<br>ko:K18052 PRKCQ; novel protein kinase C theta type [EC:2.7.11.13]
ko04935 Growth hormone synthesis, secretion and action (3)	ko:K02649 PIK3R1_2_3; phosphoinositide-3-kinase regulatory subunit alpha/beta/delta<br>ko:K04433 MAP2K6; mitogen-activated protein kinase kinase 6 [EC:2.7.12.2]<br>ko:K07187 IRS2; insulin receptor substrate 2
ko04750 Inflammatory mediator regulation of TRP channels (3)	ko:K02649 PIK3R1_2_3; phosphoinositide-3-kinase regulatory subunit alpha/beta/delta<br>ko:K04433 MAP2K6; mitogen-activated protein kinase kinase 6 [EC:2.7.12.2]<br>ko:K18052 PRKCQ; novel protein kinase C theta type [EC:2.7.11.13]
ko05016 Huntington disease (3)	ko:K03884 ND6; NADH-ubiquinone oxidoreductase chain 6 [EC:7.1.1.2]<br>ko:K04431 MAP2K7; mitogen-activated protein kinase kinase 7 [EC:2.7.12.2]<br>ko:K08269 ULK2; serine/threonine-protein kinase ULK2 [EC:2.7.11.1]
ko04624 Toll and Imd signaling pathway (3)	ko:K04431 MAP2K7; mitogen-activated protein kinase kinase 7 [EC:2.7.12.2]<br>ko:K04734 NFKBIA; NF-kappa-B inhibitor alpha<br>ko:K10380 ANK; ankyrin
ko05415 Diabetic cardiomyopathy (3)	ko:K00898 PDK2_3_4; pyruvate dehydrogenase kinase 2/3/4 [EC:2.7.11.2]<br>ko:K02649 PIK3R1_2_3; phosphoinositide-3-kinase regulatory subunit alpha/beta/delta<br>ko:K03884 ND6; NADH-ubiquinone oxidoreductase chain 6 [EC:7.1.1.2]
ko00051 Fructose and mannose metabolism (3)	ko:K00850 pfkA; 6-phosphofructokinase 1 [EC:2.7.1.11]<br>ko:K01623 ALDO; fructose-bisphosphate aldolase, class I [EC:4.1.2.13]<br>ko:K01803 TPI; triosephosphate isomerase (TIM) [EC:5.3.1.1]
ko00680 Methane metabolism (3)	ko:K00850 pfkA; 6-phosphofructokinase 1 [EC:2.7.1.11]<br>ko:K01623 ALDO; fructose-bisphosphate aldolase, class I [EC:4.1.2.13]<br>ko:K01689 ENO; enolase [EC:4.2.1.11]

Table S6 Cont.

KEGG Pathway	Mapped protein
ko04659 Th17 cell differentiation (3)	ko:K04079 HSP90A; molecular chaperone HtpG<br>ko:K04734 NFKBIA; NF-kappa-B inhibitor alpha<br>ko:K18052 PRKCQ; novel protein kinase C theta type [EC:2.7.11.13]
ko05211 Renal cell carcinoma (3)	ko:K02649 PIK3R1_2_3; phosphoinositide-3-kinase regulatory subunit alpha/beta/delta<br>ko:K02678 ETS1; C-ets-1<br>ko:K04410 PAK2; p21-activated kinase 2 [EC:2.7.11.1]
ko00030 Pentose phosphate pathway (3)	ko:K00850 pfkA; 6-phosphofructokinase 1 [EC:2.7.1.11]<br>ko:K01623 ALDO; fructose-bisphosphate aldolase, class I [EC:4.1.2.13]<br>ko:K01835 pgm; phosphoglucomutase [EC:5.4.2.2]
ko04213 Longevity regulating pathway - multiple species (3)	ko:K02649 PIK3R1_2_3; phosphoinositide-3-kinase regulatory subunit alpha/beta/delta<br>ko:K07187 IRS2; insulin receptor substrate 2<br>ko:K07198 PRKAA; 5'-AMP-activated protein kinase, catalytic alpha subunit [EC:2.7.11.11]
ko04932 Non-alcoholic fatty liver disease (3)	ko:K02649 PIK3R1_2_3; phosphoinositide-3-kinase regulatory subunit alpha/beta/delta<br>ko:K07187 IRS2; insulin receptor substrate 2<br>ko:K07198 PRKAA; 5'-AMP-activated protein kinase, catalytic alpha subunit [EC:2.7.11.11]
ko05130 Pathogenic Escherichia coli infection (3)	ko:K04346 GNA12; guanine nucleotide-binding protein subunit alpha-12<br>ko:K04410 PAK2; p21-activated kinase 2 [EC:2.7.11.1]<br>ko:K04734 NFKBIA; NF-kappa-B inhibitor alpha
ko04141 Protein processing in endoplasmic reticulum (3)	ko:K01230 MAN1A_C; mannosyl-oligosaccharide alpha-1,2-mannosidase [EC:3.2.1.113]<br>ko:K04079 HSP90A; molecular chaperone HtpG<br>ko:K04431 MAP2K7; mitogen-activated protein kinase kinase 7 [EC:2.7.12.2]
ko05214 Glioma (3)	ko:K02649 PIK3R1_2_3; phosphoinositide-3-kinase regulatory subunit alpha/beta/delta<br>ko:K05089 PDGFRB; platelet-derived growth factor receptor beta [EC:2.7.10.1]<br>ko:K08794 CAMK1; calcium/calmodulin-dependent protein kinase I [EC:2.7.11.17]
ko04064 NF-kappa B signaling pathway (3)	ko:K04734 NFKBIA; NF-kappa-B inhibitor alpha<br>ko:K05163 EDA2R; tumor necrosis factor receptor superfamily member 27<br>ko:K18052 PRKCQ; novel protein kinase C theta type [EC:2.7.11.13]
ko05414 Dilated cardiomyopathy (3)	ko:K12045 TNNT2; troponin T, cardiac muscle<br>ko:K12314 ACTC1; actin, alpha cardiac muscle<br>ko:K12567 TTN; titin [EC:2.7.11.1]

Table S6 Cont.

KEGG Pathway	Mapped protein
ko04724 Glutamatergic synapse (3)	ko:K13576 SLC38A3; solute carrier family 38 (sodium-coupled neutral amino acid transporter), member 3<br>ko:K14207 SLC38A2; solute carrier family 38 (sodium-coupled neutral amino acid transporter), member 2<br>ko:K15010 HOMER; homer
ko04530 Tight junction (3)	ko:K04431 MAP2K7; mitogen-activated protein kinase kinase 7 [EC:2.7.12.2]<br>ko:K07198 PRKAA; 5'-AMP-activated protein kinase, catalytic alpha subunit [EC:2.7.11.11]<br>ko:K12757 MYL12; myosin regulatory light chain 12
ko00240 Pyrimidine metabolism (3)	ko:K01511 ENTPD5_6; ectonucleoside triphosphate diphosphohydrolase 5/6 [EC:3.6.1.6]<br>ko:K07023 YGK1; 5'-deoxynucleotidase [EC:3.1.3.89]<br>ko:K11540 CAD; carbamoyl-phosphate synthase / aspartate carbamoyltransferase / dihydroorotase [EC:6.3.5.5 2.1.3.2 3.5.2.3]
ko05218 Melanoma (3)	ko:K02649 PIK3R1_2_3; phosphoinositide-3-kinase regulatory subunit alpha/beta/delta<br>ko:K04358 FGF; fibroblast growth factor<br>ko:K05089 PDGFRB; platelet-derived growth factor receptor beta [EC:2.7.10.1]
ko04921 Oxytocin signaling pathway (3)	ko:K03234 EEF2; elongation factor 2<br>ko:K07198 PRKAA; 5'-AMP-activated protein kinase, catalytic alpha subunit [EC:2.7.11.11]<br>ko:K08794 CAMK1; calcium/calmodulin-dependent protein kinase I [EC:2.7.11.17]
ko04080 Neuroactive ligand-receptor interaction (3)	ko:K04816 CHRND; nicotinic acetylcholine receptor delta<br>ko:K04818 CHRNG; nicotinic acetylcholine receptor gamma<br>ko:K05771 NR3C1; glucocorticoid receptor
ko05166 Human T-cell leukemia virus 1 infection (3)	ko:K02649 PIK3R1_2_3; phosphoinositide-3-kinase regulatory subunit alpha/beta/delta<br>ko:K02678 ETS1; C-ets-1<br>ko:K04734 NFKBIA; NF-kappa-B inhibitor alpha
ko04926 Relaxin signaling pathway (3)	ko:K02649 PIK3R1_2_3; phosphoinositide-3-kinase regulatory subunit alpha/beta/delta<br>ko:K04431 MAP2K7; mitogen-activated protein kinase kinase 7 [EC:2.7.12.2]<br>ko:K04734 NFKBIA; NF-kappa-B inhibitor alpha
ko05226 Gastric cancer (3)	ko:K00444 WNT5; wingless-type MMTV integration site family, member 5<br>ko:K02649 PIK3R1_2_3; phosphoinositide-3-kinase regulatory subunit alpha/beta/delta<br>ko:K04358 FGF; fibroblast growth factor

Table S6 Cont.

KEGG Pathway	Mapped protein
ko04664 Fc epsilon RI signaling pathway (3)	ko:K02649 PIK3R1_2_3; phosphoinositide-3-kinase regulatory subunit alpha/beta/delta<br>ko:K04431 MAP2K7; mitogen-activated protein kinase kinase 7 [EC:2.7.12.2]<br>ko:K04433 MAP2K6; mitogen-activated protein kinase kinase 6 [EC:2.7.12.2]
ko00330 Arginine and proline metabolism (3)	ko:K00286 proC; pyrroline-5-carboxylate reductase [EC:1.5.1.2]<br>ko:K00613 GATM; glycine amidinotransferase [EC:2.1.4.1]<br>ko:K00933 E2.7.3.2; creatine kinase [EC:2.7.3.2]
ko00620 Pyruvate metabolism (3)	ko:K00016 LDH; L-lactate dehydrogenase [EC:1.1.1.27]<br>ko:K00873 PK; pyruvate kinase [EC:2.7.1.40]<br>ko:K01946 ACACB; acetyl-CoA carboxylase / biotin carboxylase 2 [EC:6.4.1.2 6.3.4.14 2.1.3.15]
ko04013 MAPK signaling pathway - fly (3)	ko:K02678 ETS1; C-ets-1<br>ko:K04431 MAP2K7; mitogen-activated protein kinase kinase 7 [EC:2.7.12.2]<br>ko:K10176 TBX2; T-box protein 2
ko04974 Protein digestion and absorption (3)	ko:K08132 COL12A; collagen type XII alpha<br>ko:K08135 COL15A; collagen type XV alpha<br>ko:K14207 SLC38A2; solute carrier family 38 (sodium-coupled neutral amino acid transporter), member 2
ko04514 Cell adhesion molecules (3)	ko:K06491 NCAM; neural cell adhesion molecule<br>ko:K06494 SELE; selectin, endothelial cell<br>ko:K06496 SELP; selectin, platelet
ko04930 Type II diabetes mellitus (3)	ko:K00873 PK; pyruvate kinase [EC:2.7.1.40]<br>ko:K02649 PIK3R1_2_3; phosphoinositide-3-kinase regulatory subunit alpha/beta/delta<br>ko:K07187 IRS2; insulin receptor substrate 2
ko03010 Ribosome (3)	ko:K02875 RP-L14e; large subunit ribosomal protein L14e<br>ko:K02880 RP-L17e; large subunit ribosomal protein L17e<br>ko:K02934 RP-L6e; large subunit ribosomal protein L6e
ko04915 Estrogen signaling pathway (3)	ko:K02649 PIK3R1_2_3; phosphoinositide-3-kinase regulatory subunit alpha/beta/delta<br>ko:K04079 HSP90A; molecular chaperone HtpG<br>ko:K09571 FKBP4_5; FK506-binding protein 4/5 [EC:5.2.1.8]

Table S6 Cont.

KEGG Pathway	Mapped protein
ko04012 ErbB signaling pathway (3)	ko:K02649 PIK3R1_2_3; phosphoinositide-3-kinase regulatory subunit alpha/beta/delta<br>ko:K04410 PAK2; p21-activated kinase 2 [EC:2.7.11.1]<br>ko:K04431 MAP2K7; mitogen-activated protein kinase kinase 7 [EC:2.7.12.2]
ko04361 Axon regeneration (3)	ko:K04346 GNA12; guanine nucleotide-binding protein subunit alpha-12<br>ko:K04431 MAP2K7; mitogen-activated protein kinase kinase 7 [EC:2.7.12.2]<br>ko:K07198 PRKAA; 5'-AMP-activated protein kinase, catalytic alpha subunit [EC:2.7.11.11]
ko04072 Phospholipase D signaling pathway (3)	ko:K02649 PIK3R1_2_3; phosphoinositide-3-kinase regulatory subunit alpha/beta/delta<br>ko:K04346 GNA12; guanine nucleotide-binding protein subunit alpha-12<br>ko:K05089 PDGFRB; platelet-derived growth factor receptor beta [EC:2.7.10.1]
ko04929 GnRH secretion (2)	ko:K02649 PIK3R1_2_3; phosphoinositide-3-kinase regulatory subunit alpha/beta/delta<br>ko:K06250 SPP1; secreted phosphoprotein 1
ko04662 B cell receptor signaling pathway (2)	ko:K02649 PIK3R1_2_3; phosphoinositide-3-kinase regulatory subunit alpha/beta/delta<br>ko:K04734 NFKBIA; NF-kappa-B inhibitor alpha
ko04071 Sphingolipid signaling pathway (2)	ko:K02649 PIK3R1_2_3; phosphoinositide-3-kinase regulatory subunit alpha/beta/delta<br>ko:K04346 GNA12; guanine nucleotide-binding protein subunit alpha-12
ko04371 Apelin signaling pathway (2)	ko:K06250 SPP1; secreted phosphoprotein 1<br>ko:K07198 PRKAA; 5'-AMP-activated protein kinase, catalytic alpha subunit [EC:2.7.11.11]
ko04611 Platelet activation (2)	ko:K02649 PIK3R1_2_3; phosphoinositide-3-kinase regulatory subunit alpha/beta/delta<br>ko:K12757 MYL12; myosin regulatory light chain 12
ko04261 Adrenergic signaling in cardiomyocytes (2)	ko:K12045 TNNT2; troponin T, cardiac muscle<br>ko:K12314 ACTC1; actin, alpha cardiac muscle
ko00830 Retinol metabolism (2)	ko:K07249 ALDH1A; retinal dehydrogenase [EC:1.2.1.36]<br>ko:K09516 RETSAT; all-trans-retinol 13,14-reductase [EC:1.3.99.23]
ko03013 Nucleocytoplasmic transport (2)	ko:K03231 EEF1A; elongation factor 1-alpha<br>ko:K14289 XPO5; exportin-5
ko04710 Circadian rhythm (2)	ko:K02295 CRY; cryptochrome<br>ko:K07198 PRKAA; 5'-AMP-activated protein kinase, catalytic alpha subunit [EC:2.7.11.11]
ko00250 Alanine, aspartate and glutamate metabolism (2)	ko:K01939 purA; adenylosuccinate synthase [EC:6.3.4.4]<br>ko:K11540 CAD; carbamoyl-phosphate synthase / aspartate carbamoyltransferase / dihydroorotase [EC:6.3.5.5 2.1.3.2 3.5.2.3]

Table S6 Cont.

KEGG Pathway	Mapped protein
ko04625 C-type lectin receptor signaling pathway (2)	ko:K02649 PIK3R1_2_3; phosphoinositide-3-kinase regulatory subunit alpha/beta/delta<br>ko:K04734 NFKBIA; NF-kappa-B inhibitor alpha
ko05220 Chronic myeloid leukemia (2)	ko:K02649 PIK3R1_2_3; phosphoinositide-3-kinase regulatory subunit alpha/beta/delta<br>ko:K04734 NFKBIA; NF-kappa-B inhibitor alpha
ko04270 Vascular smooth muscle contraction (2)	ko:K04346 GNA12; guanine nucleotide-binding protein subunit alpha-12<br>ko:K18052 PRKCQ; novel protein kinase C theta type [EC:2.7.11.13]
ko04210 Apoptosis (2)	ko:K02649 PIK3R1_2_3; phosphoinositide-3-kinase regulatory subunit alpha/beta/delta<br>ko:K04734 NFKBIA; NF-kappa-B inhibitor alpha
ko04657 IL-17 signaling pathway (2)	ko:K04079 HSP90A; molecular chaperone HtpG<br>ko:K04734 NFKBIA; NF-kappa-B inhibitor alpha
ko00640 Propanoate metabolism (2)	ko:K00016 LDH; L-lactate dehydrogenase [EC:1.1.1.27]<br>ko:K01946 ACACB; acetyl-CoA carboxylase / biotin carboxylase 2 [EC:6.4.1.2 6.3.4.14 2.1.3.15]
ko05164 Influenza A (2)	ko:K02649 PIK3R1_2_3; phosphoinositide-3-kinase regulatory subunit alpha/beta/delta<br>ko:K04734 NFKBIA; NF-kappa-B inhibitor alpha
ko04630 JAK-STAT signaling pathway (2)	ko:K02649 PIK3R1_2_3; phosphoinositide-3-kinase regulatory subunit alpha/beta/delta<br>ko:K05089 PDGFRB; platelet-derived growth factor receptor beta [EC:2.7.10.1]
ko04062 Chemokine signaling pathway (2)	ko:K02649 PIK3R1_2_3; phosphoinositide-3-kinase regulatory subunit alpha/beta/delta<br>ko:K04734 NFKBIA; NF-kappa-B inhibitor alpha
ko03018 RNA degradation (2)	ko:K00850 pfkA; 6-phosphofructokinase 1 [EC:2.7.1.11]<br>ko:K01689 ENO; enolase [EC:4.2.1.11]
ko04727 GABAergic synapse (2)	ko:K13576 SLC38A3; solute carrier family 38 (sodium-coupled neutral amino acid transporter), member 3<br>ko:K14207 SLC38A2; solute carrier family 38 (sodium-coupled neutral amino acid transporter), member 2
ko00563 Glycosylphosphatidylinositol (GPI)-anchor biosynthesis (2)	ko:K05290 PIGK; GPI-anchor transamidase subunit K<br>ko:K07541 PIGX; GPI mannosyltransferase 1 subunit X
ko04024 cAMP signaling pathway (2)	ko:K02649 PIK3R1_2_3; phosphoinositide-3-kinase regulatory subunit alpha/beta/delta<br>ko:K04734 NFKBIA; NF-kappa-B inhibitor alpha
ko05202 Transcriptional misregulation in cancer (2)	ko:K05096 FLT1; FMS-like tyrosine kinase 1 [EC:2.7.10.1]<br>ko:K15184 AFF1; AF4/FMR2 family member 1

Table S6 Cont.

KEGG Pathway	Mapped protein
ko04022 cGMP-PKG signaling pathway (2)	ko:K04346 GNA12; guanine nucleotide-binding protein subunit alpha-12<br>ko:K07187 IRS2; insulin receptor substrate 2
ko04512 ECM-receptor interaction (2)	ko:K06250 SPP1; secreted phosphoprotein 1<br>ko:K06254 AGRN; agrin
ko04621 NOD-like receptor signaling pathway (2)	ko:K04079 HSP90A; molecular chaperone HtpG<br>ko:K04734 NFKBIA; NF-kappa-B inhibitor alpha
ko04070 Phosphatidylinositol signaling system (2)	ko:K01092 E3.1.3.25; myo-inositol-1(or 4)-monophosphatase [EC:3.1.3.25]<br>ko:K02649 PIK3R1_2_3; phosphoinositide-3-kinase regulatory subunit alpha/beta/delta
ko01521 EGFR tyrosine kinase inhibitor resistance (2)	ko:K02649 PIK3R1_2_3; phosphoinositide-3-kinase regulatory subunit alpha/beta/delta<br>ko:K05089 PDGFRB; platelet-derived growth factor receptor beta [EC:2.7.10.1]
ko00260 Glycine, serine and threonine metabolism (2)	ko:K00613 GATM; glycine amidinotransferase [EC:2.1.4.1]<br>ko:K01837 BPGM; bisphosphoglycerate/phosphoglycerate mutase [EC:5.4.2.4 5.4.2.11]
ko00562 Inositol phosphate metabolism (2)	ko:K01092 E3.1.3.25; myo-inositol-1(or 4)-monophosphatase [EC:3.1.3.25]<br>ko:K01803 TPI; triosephosphate isomerase (TIM) [EC:5.3.1.1]
ko00052 Galactose metabolism (2)	ko:K00850 pfkA; 6-phosphofructokinase 1 [EC:2.7.1.11]<br>ko:K01835 pgm; phosphoglucomutase [EC:5.4.2.2]
ko04919 Thyroid hormone signaling pathway (2)	ko:K00850 pfkA; 6-phosphofructokinase 1 [EC:2.7.1.11]<br>ko:K02649 PIK3R1_2_3; phosphoinositide-3-kinase regulatory subunit alpha/beta/delta
ko04923 Regulation of lipolysis in adipocytes (2)	ko:K02649 PIK3R1_2_3; phosphoinositide-3-kinase regulatory subunit alpha/beta/delta<br>ko:K07187 IRS2; insulin receptor substrate 2
ko04714 Thermogenesis (2)	ko:K03884 ND6; NADH-ubiquinone oxidoreductase chain 6 [EC:7.1.1.2]<br>ko:K07198 PRKAA; 5'-AMP-activated protein kinase, catalytic alpha subunit [EC:2.7.11.11]
